# Strong Magnetic Exchange Interactions and Delocalized Mn–O States Enable High-Voltage Capacity in the Na-Ion Cathode P2–Na_0.67_[Mg_0.28_Mn_0.72_]O_2_

**DOI:** 10.1021/acs.chemmater.4c01320

**Published:** 2024-09-23

**Authors:** Euan N. Bassey, Howie Nguyen, Teresa Insinna, Jeongjae Lee, Anne-Laure Barra, Giannantonio Cibin, Peter Bencok, Raphaële J. Clément, Clare P. Grey

**Affiliations:** † Yusuf Hamied Department of Chemistry, University of Cambridge, Lensfield Road, Cambridge CB2 1EW, United Kingdom; ‡ Materials Department and Materials Research Laboratory, University of California, Santa Barbara, California 93106-5050, United States; § School of Earth and Environmental Sciences, Seoul National University, Seoul 08826, Korea; ∥ Laboratoire National des Champs Magnétiques Intenses, CNRS, Univ. Grenoble-Alpes, 38042 Grenoble Cedex 9, France; ⊥ Université Grenoble Alpes, 621 Av. Centrale, 38400 Saint-Martin-d’Hères, France; # Diamond Light Source, Harwell Science and Innovation Campus, Didcot OX11 0DE, United Kingdom

## Abstract

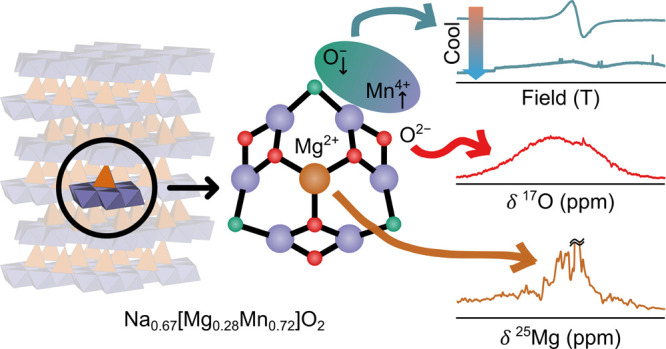

The increased capacity offered by oxygen-redox active cathode materials for rechargeable lithium- and sodium-ion batteries (LIBs and NIBs, respectively) offers a pathway to the next generation of high-gravimetric-capacity cathodes for use in devices, transportation and on the grid. Many of these materials, however, are plagued with voltage fade, voltage hysteresis and O_2_ loss, the origins of which can be traced back to changes in their electronic and chemical structures on cycling. Developing a detailed understanding of these changes is critical to mitigating these cathodes’ poor performance. In this work, we present an analysis of the redox mechanism of P2–Na_0.67_[Mg_0.28_Mn_0.72_]O_2_, a layered NIB cathode whose high capacity has previously been attributed to trapped O_2_ molecules. We examine a variety of charge compensation scenarios, calculate their corresponding densities of states and spectroscopic properties, and systematically compare the results to experimental data: ^25^Mg and ^17^O nuclear magnetic resonance (NMR) spectroscopy, *operando* X-band and *ex situ* high-frequency electron paramagnetic resonance (EPR), *ex situ* magnetometry, and O and Mn *K*-edge X-ray Absorption Spectroscopy (XAS) and X-ray Absorption Near Edge Spectroscopy (XANES). *Via* a process of elimination, we suggest that the mechanism for O redox in this material is dominated by a process that involves the formation of strongly antiferromagnetic, delocalized Mn–O states which form after Mg^2+^ migration at high voltages. Our results primarily rely on noninvasive techniques that are vital to understanding the electronic structure of metastable cycled cathode samples.

## Introduction

The total energy stored in rechargeable lithium- and sodium-ion batteries (LIBs and NIBs) is at present limited by the cathode material.^1−6^ Among the vast phase space for LIB and NIB cathode materials, layered transition metal oxide compounds, Li*TM*O_2_ and Na_*x*_*TM*O_2_ (*TM* = transition metal; 0 < *x* ≤ 1), whose two-dimensional Li and Na layers offer fast ionic diffusion (fast charge–discharge rates), are perhaps the most successful and commercially viable.^7,8^ The amount of charge stored (the capacity) at the cathode is limited by the number of redox-active centers and the number of accessible oxidation states for each of these centers. Conventionally, only the *TM* species participate in redox processes during charge and discharge,^8 ,9^ owing to their ability to readily donate and accept electrons into/from their valence *d* orbitals.

In recent years, interest in harnessing redox processes involving the oxide anion, O^2–^, has grown:^9−13^ if electrons from O^2–^ can be reversibly released and recovered during charge and discharge, the gravimetric capacity of *TM* oxide cathodes would increase significantly (assuming the structures contain sufficient Li/Na ions that can be concomitantly and reversibly extracted), enabling higher energy densities for LIBs and NIBs. Indeed, several authors have reported so-called oxygen redox, enabling capacities that far exceed “conventional” cathodes relying on *TM*-only redox.^10,11,14−18^

The premise on which oxygen redox generally relies is the generation of nonbonding lone pairs on oxygen, typically generated *via* ionic interactions with (invariably electrochemically inactive) metal cations in the TMO_2_ layer.^14,15,19−24^ In LIB cathodes, this has been most successfully achieved in “lithium-rich” systems, where the Li:*TM* ratio exceeds one and the excess Li^+^ reside in the *TM*O_2_ layers, giving rise to the desired ionic interactions.^17,25−28^ In NIB cathodes, “excess” Na^+^ materials containing the more commercially appealing 3*dTM*s are generally not possible owing to the large size of Na^+^: the strains induced by inserting Na^+^ into the *TM*O_2_ layer are often too great^29^ and the as-synthesized material forms Na oxides in addition to a stoichiometric or substoichiometric cathode material.^30^ Instead, authors have successfully doped other metal cations, for example, Li^+^, Mg^2+^, Zn^2+^ and even vacancies into the *TM*O_2_ layer to achieve these ionic interactions.^14−16,19−24^

While the generation of high-energy, redox-accessible oxygen-based lone-pair(-like) electrons is widely accepted, the stabilization of oxidized oxygen species—and therefore the charge compensation mechanism—is still under debate within the battery and solid-state chemistry communities. Several stabilization mechanisms, including localized O holes,^31−33^ formation of peroxo-like (O_2_)^*n*−^ species,^34−36^ trapped molecular O_2_,^15,37,38^*TM* migration induced O^2–^ oxidation^17,18,39^ and π-redox,^40^ have been proposed, with no clear consensus, in part because there is likely no single mechanism that operates in all these different systems. Furthermore, when examining oxygen redox, a single or perhaps two techniques are often used to deduce a mechanism, and the mechanisms proposed may differ from those obtained by other research groups using different methods. The reader is directed toward refs (9,10,41) for a comprehensive examination of each of these charge compensation mechanisms.

Oxygen redox is of particular interest to NIB cathodes, where a sustainable, low-cost ethos drives the development and optimization of these systems. Among the NIB cathodes known to exhibit oxygen redox, one of the most intriguing materials is Na_0.67_[Mg_0.28_Mn_0.72_]O_2_ (henceforth NMMO), first reported by Yabuuchi *et al*.,^19^ whose first-charge plateau is accompanied by a large voltage hysteresis, but with few phase changes during cycling, making it an attractive potential candidate for long-term cycling applications.

**Figure 1:**
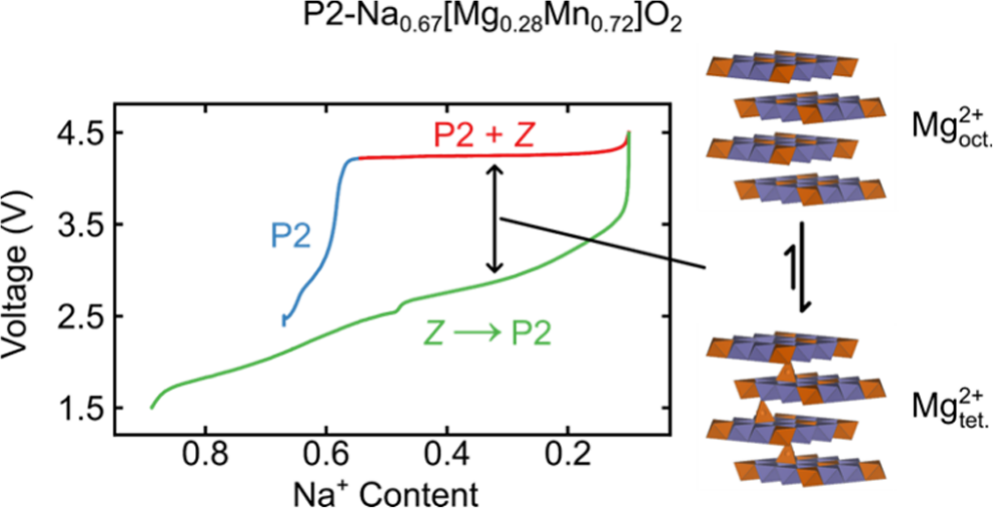
Summary of the structural changes of Na_0.67_[Mg_0.28_Mn_0.72_]O_2_ during the first charge–discharge cycle, highlighting the contribution of Mg^2+^ migration to the observed voltage hysteresis.

Subsequent studies by Maitra *et al*.,^14^ House *et al*.^22^ and by us^42,43^ described the structural changes that occur during the first charge–discharge cycle of NMMO: an initial single-phase desodiation process was seen, which was followed by a high-voltage two-phase reaction process between P2 and a *Z*-phase (an intergrowth of P- and O-type layers with no long-range order of the layers). Experimental data was consistent with Mg^2+^ migration from octahedral sites in the TMO_2_ layers to tetrahedral sites in the vacant O-type Na^+^ layers at the end of charge,^22,42^ and it was shown^42^ that this migration was both kinetically and thermodynamically feasible using *ab initio* transition state searches (Figure 1). While Boivin *et al*. subsequently proposed a charge compensation scheme involving Mg^2+^ migration and concomitant O_2_ trapping, their work did not consider additional charge compensation schemes reported in the literature and relied heavily on *ex situ* resonant inelastic X-ray scattering (RIXS), a highly invasive technique for interrogating electronic and structural changes, performed using high-energy X-rays while under high vacuum.^22^

In this study, we screen possible charge compensation scenarios in NMMO, combining computations and experiments, and then compare and contextualize our results to mechanisms proposed in the literature. Novel ^25^Mg nuclear magnetic resonance (NMR) measurements confirm that Mg^2+^ migration takes place at high voltages in NMMO. The consequences of this migration on the electronic structure and charge compensation process are evaluated using *ab initio* calculations of the density of states, *ex situ*^17^O NMR, bulk magnetic susceptibility measurements, *operando* X-band and *ex situ* high-frequency electron paramagnetic resonance (EPR) spectroscopy, Mn *K*-edge X-ray absorption near edge spectroscopy (XANES) and O *K*-edge X-ray absorption spectroscopy (XAS). This complements our previous studies on the Na^+^ dynamics^43^ and structures of the pristine and cycled material.^42^ We note that we are not presenting ^23^Na NMR here; this was covered in our previous work.^42,43^*Via* a simple process of elimination,^44^ we reach a mechanistic description which faithfully describes all data obtained (including that obtained by previous work^14,19,22^); our results provide a truly holistic examination of the charge compensation mechanism in NMMO and highlight the benefits and pitfalls of each technique used to probe these fascinating and elusive mechanisms.

## Experimental Section

### Synthesis

A detailed synthetic route and sample preparation scheme is detailed in the Supporting Information (SI). Na_0.67_[Mg_0.28_Mn_0.72_]O_2_ was synthesized *via* a high-temperature solid-state reaction as described previously.^14,19,22^ Stoichiometric quantities of Na_2_CO_3_, MgO, and Mn_2_O_3_ were ball milled together (400 rpm, 2 h total), pressed into a pellet and heated to 1073 K (10 K min^–1^) for 10 h under flowing O_2_ (<30 mL min^–1^), followed by a natural cooling process. The pellet was ground in an Ar-filled glovebox (H_2_O and O_2_ < 1 ppm), reheated to 973 K (10 K min^–1^) and then immediately quenched to room temperature. For ^25^Mg enrichment, the same synthetic procedure was carried out, using ^25^MgO as the starting reagent. For ^17^O enrichment, approximately 150 mg of as-synthesized Na_0.67_[Mg_0.28_Mn_0.72_]O_2_ was taken, packed into an alumina crucible and then loaded into a quartz tube which was subsequently evacuated and then refilled with ^17^O_2_ gas (Nukem, 70 atom % enrichment) to a pressure of approximately 1.1 bar. The tube was then sealed and reheated to 973 K for 24 h, before being quenched to room temperature and immediately transferred to an Ar-filled glovebox.

Cathodes of Na_0.67_[Mg_0.28_Mn_0.72_]O_2_ were prepared by dispersing a mixture of the pristine powder, carbon super P (TIMCAL) and Kynar poly(vinylidene difluoride) (PVDF, Akerma) in *N*-methyl-2-pyrrolidone (NMP; Sigma-Aldrich, anhydrous, 99.5%); the mass ratio of these powders was 8:1:1. The slurry was blade-coated at a wet film thickness of 500 μm on an aluminum foil current collector and the films dried at ambient temperature under dynamic vacuum for at least 12 h. Circular electrodes (13 mm diameter) were punched out and then dried at 393 K for 12 h under dynamic vacuum; the active material loading was 1.5–9.5 mg cm^–2^; the former used for X-ray diffraction (XRD), XAS, and XANES measurements, and the latter for NMR, EPR and bulk magnetic susceptibility measurements.

For all experiments, a 1.0 M NaPF_6_ (Acros Organics, 98.5+%; dried at 393 K for 12 h under dynamic vacuum) in propylene carbonate (PC; Solvionic, <50 ppm of H_2_O) electrolyte was used unless otherwise stated. All cells in this work were half-cells, using a Na metal disc as the anode; these disks were punched out from Na metal (Sigma-Aldrich, 99.0%, 13 mm diameter). All electrochemical tests were carried out using NMMO/Na metal half-cells. Each cell was assembled from a stack of one cathode, one glass fiber separator (Whatman, GF/B, 0.68 mm thick, 16 mm diameter, 1.0 μm pore size) soaked with 150 μL electrolyte and one Na metal disc.

Electrochemical measurements on these half cells were performed using either a BioLogic MPG2 potentiostat/galvanostat instrument running EC-Lab software (^17^O and ^25^Mg NMR and high-frequency EPR experiments), or an Arbin potentiostat/galvanostat (Mn *K*-edge XANES, O *K*-edge XAS and bulk magnetic susceptibility measurements). The half cells were galvanostatically charged at a rate of 10 mA g^–1^ (corresponding to approximately *C*/19, for a theoretical *C* rate determined from the time elapsed and current applied, assuming that *x* in Na_*x*_[Mg_0.28_Mn_0.72_]O_2_ runs between 0 and 1 and that no parasitic reactions take place during cycling). This slow cycling rate was chosen to minimize effects of concentration gradients in the active material and to avoid the high overpotentials often seen at high voltages.

### *Ex Situ* Sample Preparation

Cathodes of NMMO were cycled up to a given cutoff voltage and then allowed to relax for at least 1 h (see SI, Table S1 for a list of cutoff voltages and open circuit voltages), before being extracted from the cell inside an Ar-filled glovebox and either scraped off the Al foil backing (for NMR, EPR, synchrotron X-ray diffraction (SXRD), and bulk magnetic susceptibility measurements) or peeled off the Al foil intact (for XANES and XAS measurements).

### Solid-State ^25^Mg Nuclear Magnetic Resonance Spectroscopy

^25^Mg-enriched *ex situ* cycled cathodes and pristine powders were packed into 4 mm diameter ZrO_2_ magic angle spinning (MAS) rotors in an Ar-filled glovebox, with inert poly(tetrafluoroethylene) (PTFE) tape packed at either end to ensure the sample was centered in the rotor. No rotor spent longer than 5 min outside of the glovebox before being inserted into the magnet under a protective atmosphere of flushing nitrogen gas. ^25^Mg NMR spectra were referenced to solid MgO at 26.0 ppm as an external reference. NMR spectra were acquired on a Bruker Avance III (16.4 T) using a Bruker 4 mm MAS probe; spectra were recorded static, with an effective  pulse length of 2.21 μs (which corresponds to , to account for the strong quadrupolar interaction of ^25^Mg and excite the central and satellite transitions equally—*i.e.*, the linear regime^45^). Hahn-echo pulse sequences (90°−*τ*–180°−*τ*–acquire) at different receiver frequency offsets were used to record individual slices in a variable-offset cumulative spectrum (VOCS). The recycle delay (10 ms) was set such that the bulk, paramagnetically shifted signal was recorded quantitatively. A background spectrum (acquired *via* VOCS using the same number of scans, recycle delay and receiver offset frequencies) was also obtained and subtracted from the observed spectra. Spectra were fit using dmfit,^46^ using the Int2QUAD model to account for both the quadrupolar and dipolar hyperfine interactions (CSA was used as a surrogate for the dipolar hyperfine interaction).

### Solid-State ^17^O Nuclear Magnetic Resonance Spectroscopy

^17^O-enriched pristine powder and *ex situ* cycled cathodes were packed into 1.3 mm diameter ZrO_2_ MAS rotors in an Ar-filled glovebox; no rotor spent longer than 5 min outside of the glovebox before being inserted into the magnet under a protective atmosphere of flushing nitrogen gas. ^17^O NMR spectra were referenced to liquid H_2_^17^O (0 ppm). NMR spectra were acquired on a Bruker Avance III (11.7 T) using a Bruker 1.3 mm MAS probe. A MAS frequency of 60 kHz was used, with an effective  pulse length of 0.66 μs for ^17^O to account for the strong quadrupolar interaction of and excite the central and satellite transitions equally.^45^ A rotor-synchronized Hahn-echo pulse sequence (90°−*τ*–180°−*τ*–acquire) was used. Spectra were scaled according to the mass of the sample and number of residuals recorded. The recycle delay (5 ms; at least 5*T*_1_) was set such that the bulk, paramagnetically shifted signal was recorded quantitatively, while the diamagnetic signal due to electrolyte decomposition products was suppressed. Projection magic angle turning phase-adjusted sideband separation (pjMATPASS) experiments were also recorded to separate the isotropic resonances from the overlapping spinning sideband manifold.^47,48^ Spectra were fit using dmfit,^46^ using the Int2QUAD model to account for both the quadrupolar and dipolar hyperfine interactions (CSA was used as a surrogate for the dipolar hyperfine interaction).

### Synchrotron X-ray Diffraction

*Ex situ* diffraction patterns of both the pristine material and cycled cathodes were recorded at beamline I11 at the Diamond Light Source,^49,50^ with a wavelength of 0.82652 Å over a range 2*θ* = 0 to 150°. Samples were loaded into borosilicate glass capillaries (outer diameter 0.5 mm) inside an Ar-filled glovebox and sealed using two-component epoxy resin. Rietveld refinements of the diffraction patterns were carried out using the TOPAS Academic 6.0 software package.^51^

### *Ab Initio* Density of States Calculations and Chemical Shift Calculations

To simplify calculations, a model system, O2–Na_0_[Mg_1/3_Mn_2/3_]O_2_, was constructed, using the lattice parameters and atomic coordinates obtained from *ex situ* synchrotron X-ray diffraction at the end of charge. For most calculations, all Mg^2+^ centers were placed in the tetrahedral sites in the vacant Na^+^ layers; additional calculations in which only a fraction of the Mg^2+^ centers or none of the Mg^2+^ centers were displaced were also carried out and may be found in the SI (Section 2, Tables S2–S6 and Figures S1–S3). Throughout the calculations, (2 × 2 × 1) supercells were constructed to account for both inter- and intralayer exchange interactions between the paramagnetic centers, as well as to capture the ordering seen in the pristine material. The initial charge distributions are detailed in the Results section.

The density of states was calculated for a series of charge compensation schemes, all based on the Na_0_[Mg_1/3_Mn_2/3_]O_2_ model system described in the results, using both the VASP^52−54^ and CRYSTAL codes.^55^ In VASP, the projector-augmented wave method (PAW)^56,57^ was employed, with a spin-polarized Perdew–Burke–Ernzerhof exchange correlation functional and Hubbard *U* model^58,59^ applied within the rotationally invariant formalism proposed by Liechstenstein *et al*.,^60^ to correct for the known deficiencies of pure functionals for highly localized 3*d* states.^61^ The plane-wave energy cutoff was set to 520 eV, and an effective Hubbard *U* parameter for Mn, *U*_eff_ = *U* – *J* = 3.9 eV, where *U* and *J* are the effective on-site Coulomb and exchange parameters (*J* = 1 eV), respectively, was chosen, in line with previous work on the parent material, Na_*x*_MnO_2_.^35,62^ SCF cycles were converged with an energy tolerance of 10^–8^ eV and the Brillouin zone was samples with a Monkhorst–Pack^63^ γ-centered *k*-mesh of density <0.025 Å^–1^.

Periodic spin-polarized density functional theory (DFT) calculations of the density of states and hyperfine and quadrupole-induced shifts were performed in CRYSTAL.^55^ Hyperfine parameters were calculated with B3LYP^64,65^ and a modified B3LYP hybrid functional containing 20 and 35% Hartree–Fock exchange, referred to as Hyb20 and Hyb35, respectively. These weights were chosen based on the success of these functionals in calculating the properties of TM compounds and have been previously reported to provide an upper and lower bound on experimental shifts.^66−68^

The calculations employed two basis sets: a smaller basis set for geometry optimizations (denoted BS-I) and a more extended set for the single-point hyperfine calculations (BS-II). Geometry optimizations were carried out on a ferromagnetic configuration, with an energy tolerance of 10^–5^ Hartree, a root-mean-square force gradient tolerance of 3 × 10^–4^ Hartree and integral thresholds of 10^–8^, 10^–8^, 10^–8^, 10^–8^, and 10^–16^ for the Coulomb overlap, Coulomb penetration, exchange overlap and *g* and *n* series exchange penetration, respectively. The BS-I sets were taken—without modification—from solid-state studies by Catti *et al*.,^69−72^ while the BS-II sets comprised bases from the Ahlrichs set for metal ions^73^ and IGLO-III basis set for O.^74^ Single-point calculations to obtain the density of states were performed using integral thresholds of 10^–7^, 10^–7^, 10^–7^, 10^–7^, and 10^–17^ for the Coulomb overlap, Coulomb penetration, exchange overlap and *g* and *n* series exchange penetration, respectively, while the energy tolerance was set to 10^–8^ Hartree. These calculations were carried out using Monkhorst–Pack^63^ γ-centered *k*-mesh with density <0.025 Å^–1^. Note that approximate oxidation states were obtained from the converged spin densities of each atom in the structure. Additional computational details, including the number of Gaussian primitives and the contraction scheme used for each basis set are provided in the SI (Section 1).

### Bulk Magnetic Susceptibility Measurements

The bulk magnetic susceptibility measurements were carried out on powder samples and *ex situ* cycled cathodes (approximately 3–15 mg) using a Quantum Design Magnetic Property Measurement System 3 (MPMS) superconducting quantum interference device (SQUID) magnetometer. The zero-field cooled (ZFC) and field-cooled (FC) susceptibilities were measured in a field of 0.1 T over a temperature range 2–300 K. As *M*(*H*) is linear in this field range, the small-field approximation to the susceptibility, , was assumed to be valid. The data for each sample were corrected for diamagnetism of the sample using Pascal’s constants.^75^

### *Operando* Electron Paramagnetic Resonance Spectroscopy

*Operando* EPR measurements were carried out on an in-house quartz cell^76^ containing the following stack: an Al foil current collector (5 mm diameter), a cathode disc of NMMO (5 mm diameter), a glass fiber separator (Whatman, GF/B, 0.68 mm thick, 5 mm diameter, 1.0 μm pore size) soaked in 8 μL of electrolyte (1.0 M NaPF_6_ in a 1:1:1 volumetric mixture of ethylene carbonate, diethylene carbonate and dimethyl carbonate), a Na metal foil disc (5 mm diameter) and a Cu foil current collector. A ruby reference was glued to the outside of the cell using two-component epoxy. The cell was cycled at a rate of 10 mA g^–1^ between 1.5 and 4.5 V *vs* Na^0/+^. The cell was placed in a high-*Q* cavity (Bruker 4119HS-LC), and spectra were recorded continuously during cycling, with 420 s between each spectral slice; these slices comprised four individual spectra, to improve signal-to-noise. A microwave power of 6.325 mW was used, with a modulation amplitude of 4 G.

### High-Frequency Electron Paramagnetic Resonance Spectroscopy

The high-frequency EPR (HF-EPR) spectra of pristine powders and *ex situ* cycled cathodes were recorded on a double-pass transmission EPR spectrometer built at the high magnetic field laboratory in Grenoble, France.^77^ The frequencies were varied from 255 to 383 GHz using a 127 GHz frequency source and its multipliers (Virginia Diodes), while detection was achieved using a bolometer (QMC instruments). Temperatures were recorded using a variable-temperature insert (Cryogenic) at 5, 50, and 150 K. The EPR spectra were fitted to a powder pattern line shape with axial or isotropic *g* tensors using the EasySpin toolbox for MATLAB.^78^ Note that the fitted *g*-factors presented are adjusted from those generated by EasySpin, owing to the nonlinear observed data being fit in a linear fashion; see SI, Section 9, Figure S23 for further details.

### Mn *K*-Edge X-ray Absorption Near Edge Spectroscopy

*Ex situ* XANES was performed at beamline B18 at the Diamond Light Source. Mn *K*-edge data were recorded at ambient temperature in transmission mode above and below the absorption edge (6539 eV). Samples were loaded into an in-house (Diamond) transfer chamber with transparent polyimide (Kapton) film windows. Three spectral scans were recorded for each sample; no changes between the first and last measurements were observed. Alignment, background removal and fitting of the XANES data was carried out in the Athena software package.^79,80^

### O *K*-edge X-ray Absorption Spectroscopy

*Ex situ* XAS was performed at beamline I10 at the Diamond Light Source. O *K*-edge data were recorded at ambient temperature in both fluorescence yield (FY) and total electron yield (TEY) modes above and below the absorption edge of 543 eV. Samples were loaded onto an in-house (Diamond) transfer module using a conductive epoxy resin. Four spectral scans were recorded for each sample; no drift in the beam energy was observed between the first and last measurement. Alignment, background removal and fitting of the data was carried out in the PyMCA software package.^81^

## Results

### Electrochemistry

**Figure 2:**
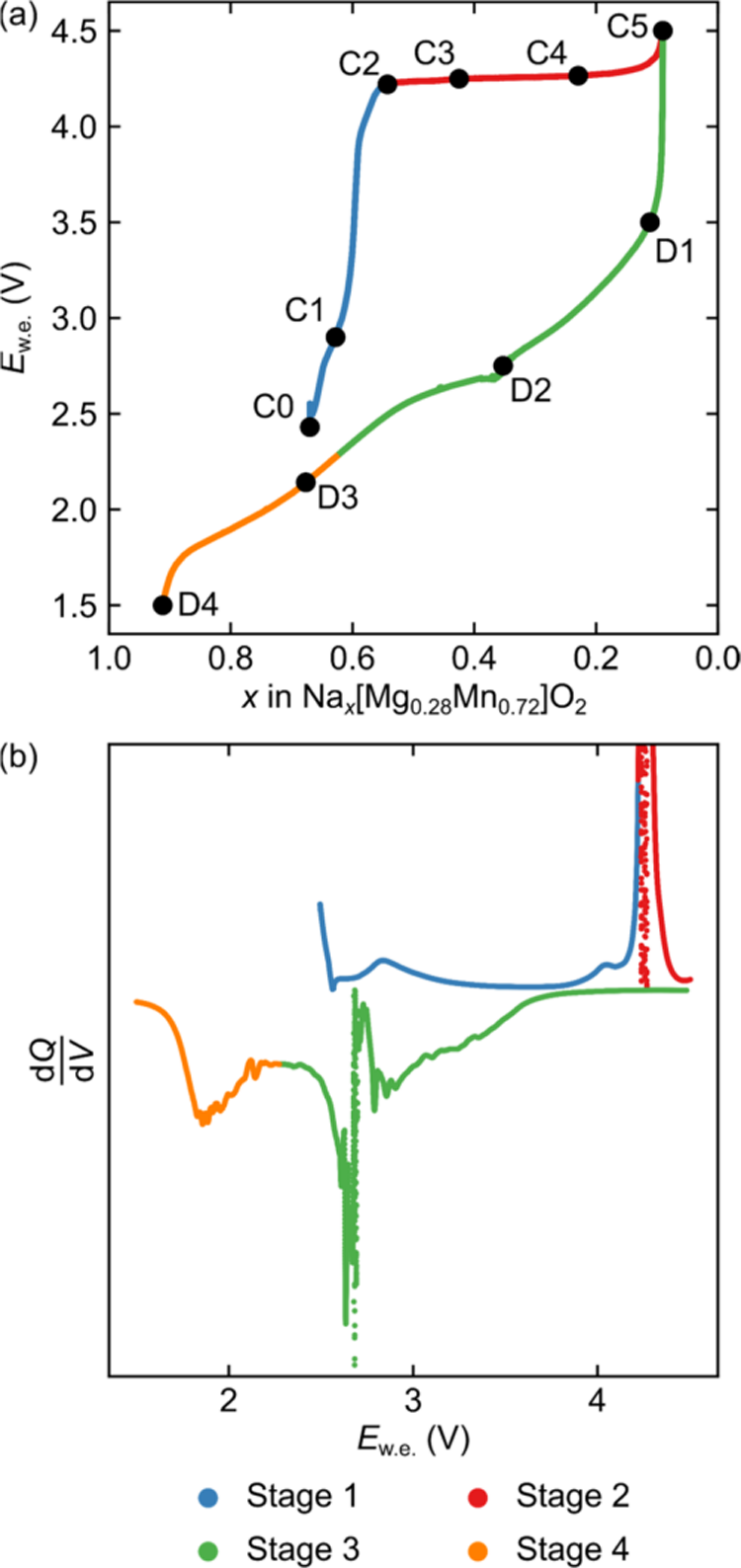
Electrochemical data for the first charge–discharge cycle of NMMO. (a) shows the voltage profile with *ex situ* sampling points labeled, while (b) shows the differential capacity-voltage profile. The four stages of the charge–discharge cycle are also highlighted.

The electrochemical performance of NMMO in a half cell versus Na metal has been presented in our previous study.^42^ To summarize, an initial increase in voltage is seen (Stage 1 in Figure 2), corresponding to single-phase Na^+^ extraction; this is followed by a long voltage plateau (Stage 2), arising from the two-phase reaction between the P2 and Z-phases of NMMO (Stages 3 and 4) and then a sloping region throughout discharge, corresponding to reinsertion of Na^+^ in which O-type layers gradually transform into P-type; this manifests as a single, continuous phase transformation but likely corresponds to a series of overlapping processes. In addition, a large voltage hysteresis is observed (Figure 2a), which has been ascribed to Mg^2+^ migration from octahedral sites in the TMO_2_ layers to tetrahedral sites in the vacant O-type layers of Z-phase NMMO on charge.^42^

The differential voltage-capacity  curve over the first charge–discharge cycle reveals the four distinct regions readily and may also be used to delineate the different redox regimes (Figure 2b). Boivin *et al*. assigned stages 1 and 4 to Mn oxidation and reduction, respectively, since the pristine material has an average Mn oxidation state of 3.85+, while stages 2 and 3 were assigned to O oxidation and reduction with stabilization *via* the formation of molecular oxygen, O_2_.^22^ The points labeled on the charge–discharge curve (C0 through to C5 for charge and D1 to D4 for discharge) are the points at which *ex situ* measurements were carried out in the current study; the corresponding compositions and average Mn oxidation states (assuming Mn-only redox) are given below in Table 1.

**Table 1:** Compositions and Average Mn Oxidation States (Assuming Pure Mn Redox) of *Ex Situ* Cycled Cathodes, Determined from the Time Elapsed since the Start of Cycling and the Current Applied, Assuming that *x* in Na_*x*_[Mg_0.28_Mn_0.72_]O_2_ Runs between 0 and 1, that *x* in the Pristine Material is 0.67, and that No Parasitic Reactions Take Place During Cycling

sample	*x* in Na_*x*_[Mg_0.28_Mn_0.72_]O_2_	oxidation state of Mn for Mn-only redox
C0	0.67	+3.85
C1	0.62	+3.92
C2	0.54	+4.03
C3	0.42	+4.20
C4	0.40	+4.22
C5	0.10	+4.64
D1	0.13	+4.60
D2	0.38	+4.25
D3	0.66	+3.86
D4	0.89	+3.54

### Possible Charge Compensation Schemes

To assist the interpretation of the *ex situ* and *operando* data acquired for NMMO, we consider four possible charge compensation schemes and compare *ab initio* calculated quantities (density of states, DOS, and NMR shifts) against observations.

The charge compensation schemes are (A) localized holes on O (*i.e.*, O^–^);^33^ (B) Mn^5+^ formation;^82^ (C) delocalized or π-like redox; (D) trapped O_2_ and peroxo-like ((O_2_)^*n*−^) species formation,^15,22,34,35,37,38,40,83−88^ While a final scenario, the formation of Mn^7+^, has also been hypothesized,^89,90^ its formation likely requires the formation of an intermediate species, such as Mn^5+^ (from (B)), or disproportionation from Mn^4+^ to, for example, Mn^2+^ and Mn^7+^. The calculation of Mn^5+^-based states should capture any tendency to form Mn^7+^*via* a Mn^5+^ intermediate. We note also that, of the species, Mn^*n*+^ (*n* = 2, 4, and 5) and Mg, the latter is more likely to prefer tetrahedral sites and have a much lower barrier to migration, as shown previously.^42^

**Figure 3:**
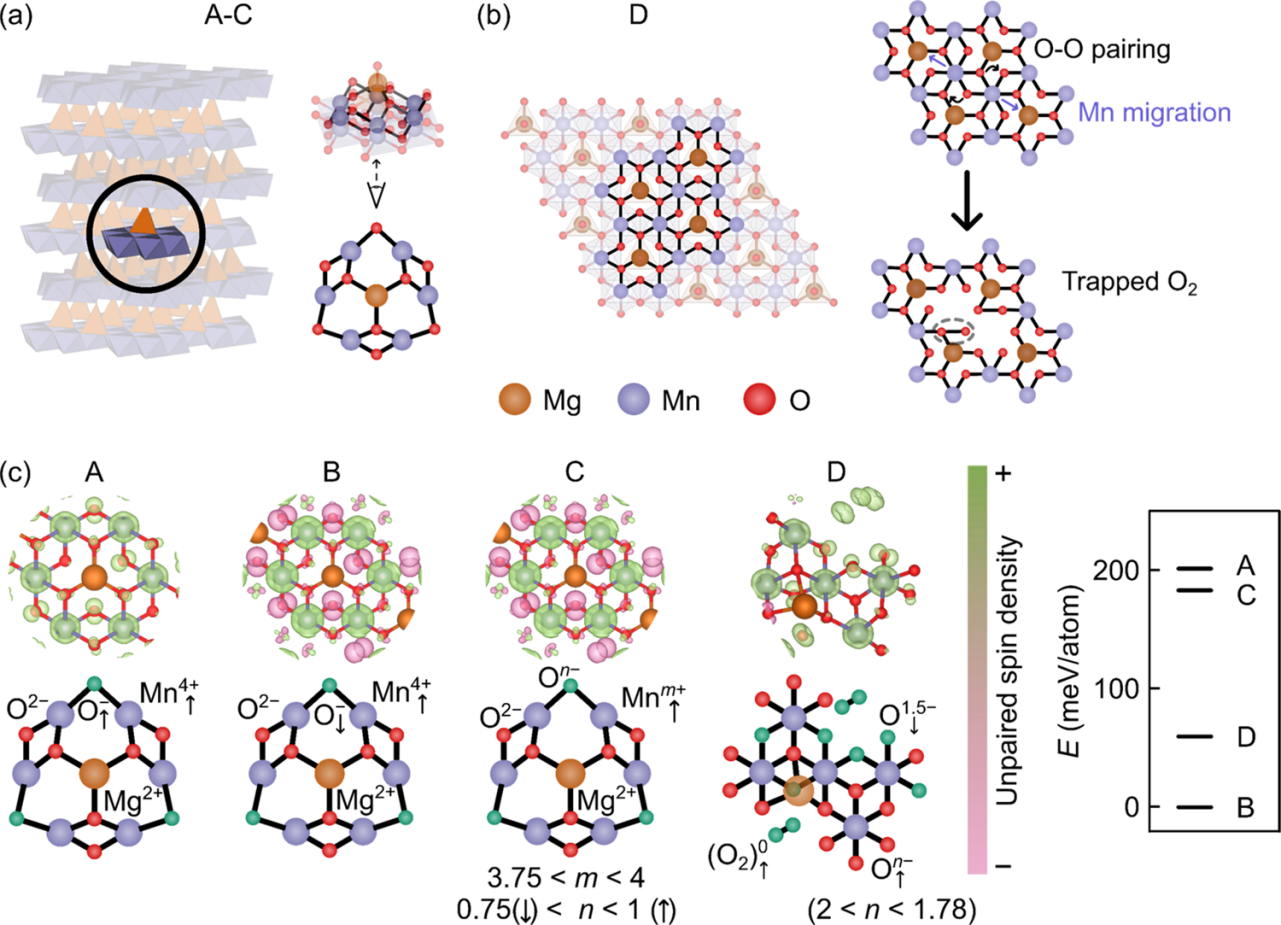
Schematic charge compensation schemes and calculated spin density maps after relaxation explored for NMMO. (a) shows the structure of Mg-migrated O2-NMMO (left), used to describe schemes A to C with the MgMn_6_O_9_ cluster highlighted (right). In (b), the rearrangement on the Mn/Mg and O sublattices for scheme D is shown. (c) shows the spin density maps (left) and relative energies (right) of each of the charge compensation schema A to D. The up and down arrows next to atomic labels indicate whether a spin was polarized up or down.

In each case, the models contain no Na^+^ since the O2-like phase present at the end of charge is fully desodiated (residual Na^+^ seen at C5 by ^23^Na NMR occupies the P-type layers^42^), all Mg^2+^ occupies tetrahedral environments in the vacant Na^+^ layer (Figure 3a), and all paramagnetic centers are ferromagnetically aligned. In D, a rearrangement within the TM layer takes place, whereby two Mn centers move into vacancies generated by Mg^2+^ migration (Figure 3b), and four O species pair up to give O_2_-like species weakly bound to Mn center(s). Note that, in some cases, the spin orientations and amount of unpaired spin density at each nuclear position evolved from the original input to the final converged system, as detailed in Table S2 (Figure 3). Additional calculations on the antiferromagnetic case are described later and in the SI (Figure S2).

We recognize that our structural models represent the ideal (honeycomb-ordered) material, and deviations could lead to the formation of other local structures or changes in the relative stabilities of different oxygen configurations, perhaps leading to identification of other modes of oxidation as minor contributions, as discussed later.

For the sake of completeness, a final scenario, in which Mg^2+^ remains in the TMO_2_ layer of O2-NMMO, was also simulated. There, only one charge compensation scheme was generated, where charge is shared over both Mn and O (*i.e.*, Mn^3.5+^ and O^*n*–^ species, *n* ∼ 1.5, formed); this is denoted as state E. This was approximately 400 meV/atom higher in energy than all other states and as such is not considered further in our discussion. Note that, in the presentation of results below, the oxidation states of the metal centers are determined from their spin densities and Bader charges.

The final (relaxed) structures and compositions, for each compensation mechanism, are listed in the SI (Section 2) and are depicted in Figure 3c. Briefly, scenarios A, B, and C are related, whereby the charge is compensated by oxidation of Mn and O to yield O– (A and B; A with the spins on Mn^4+^ and O– parallel and B with these spins antiparallel) or to yield more delocalized, mixed oxidation states (C; where Mn is in either the +4 or the +3.75 oxidation state and O is in the −1, −0.75, or −2 (formal) oxidation state; Figure 3c). We note that, while the spin density profiles of B and C are similar in Figure 3c, they differ by the oxidation states of each O and Mn center.

The charge in D is compensated by the formation of O_2_ and by partial oxidation to form hole-like states on nearby O that are part of the regular oxide framework; Mn remains in the +4 oxidation state.

Note that B, where Mn^5+^ was given as the input, always relaxed to give Mn^4+^ and O^–^ with antiparallel spins. This state is the lowest in total energy, but state D is only slightly higher in energy (Figure 3c, right). We note also that states A and C lie higher in energy than B and D, suggesting that these are less likely to form. Yet, while D is low in energy, the barrier for Mn^4+^ to migrate between octahedral sites in the TM layers migration is likely to be high, due to the instability of the tetrahedral Mn^4+^ transition state, and this state may not be observed experimentally.

### Density of States Calculations

**Figure 4:**
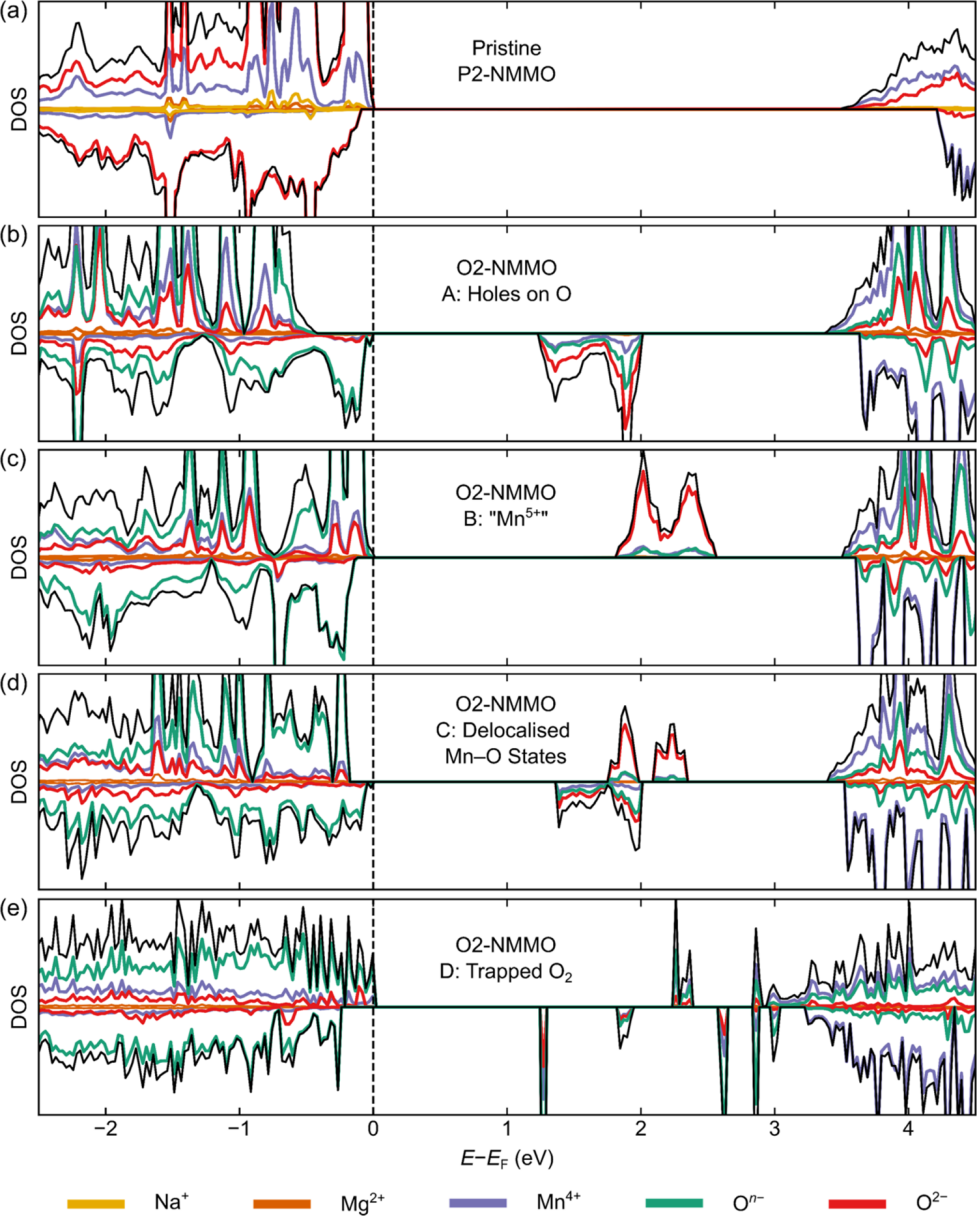
*Ab initio* density of state (DOS) plots for each of the O2-NMMO charge compensation schemes: (a) shows the pristine DOS, while (b) to (e) show the DOSs for schemes A to D, respectively. DOSs were calculated using a hybrid DFT functional with 20% Hartree–Fock exchange. The upward- and downward-facing DOSs correspond to the spin up and down bands, respectively.

To understand the electronic structures associated with each of the schemes A to E, we carried out *ab initio* density of states (DOS) calculations and the results, shown in Figure 4, are compared to the DOS of the pristine NMMO model (P2–Na_2/3_[Mg_1/3_Mn_2/3_]O_2_) from our earlier work.^42^ The DOS for pristine NMMO is as expected: a set of states below the Fermi level, *E*_F_, dominated by contributions from O and a set of states above *E*_F_ dominated by contributions from Mn (Figure 4). Since in this pristine model all Mn are in the +4 oxidation state, the model is a good approximation of the TM oxidation limit (*i.e.*, the point beyond which the anomalously large capacity is extracted). Given that O dominates the states just below *E*_F_, O species are expected to contribute to the charge compensation mechanism (to some extent) past that point.

Turning to the end of charge stoichiometry (Na_0_[Mg_1/3_Mn_2/3_]O_2_), under all schemes (A to D) the highest occupied states in NMMO are dominated by the O centers with unpaired electron density, further suggesting that O dominates the redox properties of NMMO at high states of charge. The lowest unoccupied states vary across the schemes: in A, B, and C, these states are relatively broad in bandwidth and dominated by O^2–^, with approximately equal contributions from Mn^4+^ and paramagnetic O^*n*–^ species; in D, these states are sharp and dominated by paramagnetic O in O_2_-like pairs; and in E, dominated by paramagnetic O^*n*–^, with metallic-like behavior (*i.e.*, a nonzero DOS at *E*_F_; this is attributed to thermal smearing in the calculations, rather than a genuine effect).

In scheme B, where O^*n*–^ -like species are present at the end of charge, the magnetic exchange coupling constant is approximately −197 K (determined from a magnetic cluster expansion; see SI Section 2). We anticipate that this coupling will not be directly experimentally observed, as only bulk magnetic measurements are made here; it should, however, be captured in the Weiss constant (see bulk magnetic susceptibility results).

Since B and D are well separated from states A, C, and E in energy, we will only continue to focus on these first two states for the remainder of the study for the sake of brevity and clarity. An analysis of these latter states in the context of the experimental data is presented in the SI, but we note that none of A, C, or E are consistent with all pieces of the experimental data presented below.

### Mg^2+^ Migration Investigation

*Ex situ* synchrotron X-ray diffraction (SXRD) patterns and ^25^Mg NMR spectra were obtained on pristine NMMO, and on NMMO samples collected at the end of charge (labeled point C5 in Figure 2a) and on discharge (point D3) to investigate the role of Mg^2+^ migration in the electrochemical processes.

**Figure 5:**
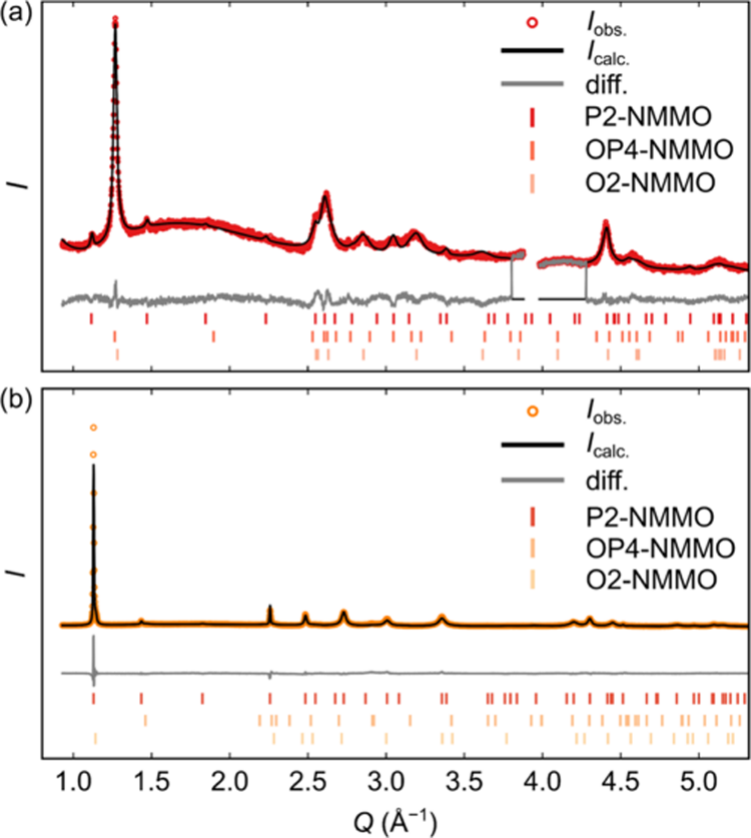
*Ex situ* Rietveld refinements to the synchrotron X-ray diffraction patterns collected at (a) the end of charge, *R*_w.p._ 14.72% and (b) at D3 on discharge, *R*_w.p._ 7.29%.

Rietveld refinements of *ex situ* SXRD patterns revealed that only the P2 phase is present at points C0 (pristine) and D3 (discharged), while C5 (end of first charge) contains only the OP4 and O2 phases, consistent with ref (42) (Figure 5). In C5, the O2 and OP4 phases that comprise the *Z*-phase (proposed in ref (42)) contain Mg^2+^ centers that have migrated into the vacant Na^+^ layer. While the reflections in the diffraction patterns are broad and an accurate refinement of the site occupancies is challenging, the results are nevertheless consistent with Mg^2+^ migration taking place in NMMO at high states of charge, as demonstrated in our prior work.^42^

**Figure 6:**
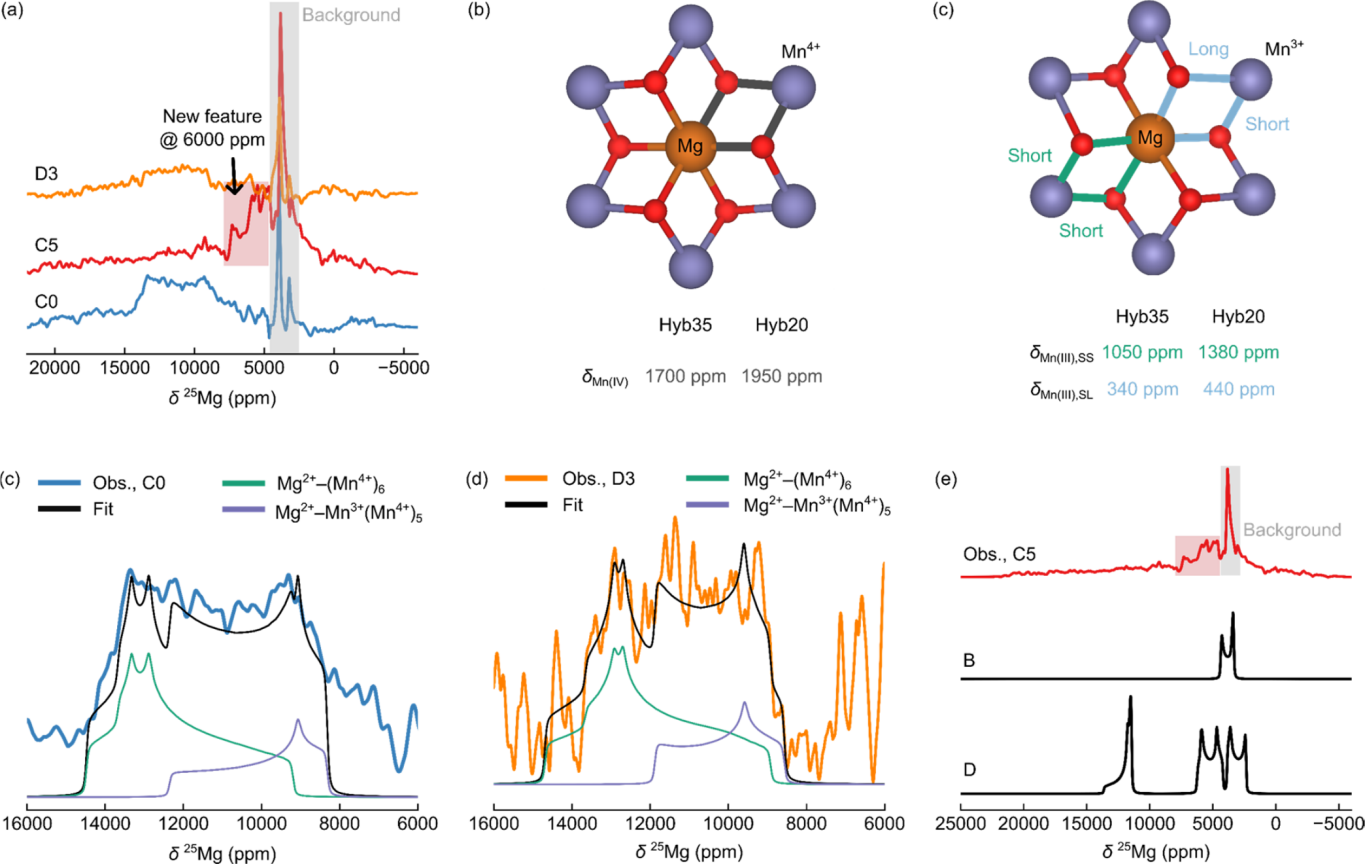
*Ex situ* static ^25^Mg Hahn-Echo VOCS data recorded at 16.4 T for pristine NMMO; the total acquisition time for each sample was 1 week, even with ^25^Mg-enriched samples. In (a), the spectra at each state of charge are shown, scaled by mass and number of scans. Resonances from the probe background are highlighted in gray. The hyperfine bond pathways are highlighted in (b), from Mn^4+^ to Mg^2+^ (gray) and (c) from Mn^3+^ to Mg^2+^*via* two Jahn–Teller (JT) shortened Mn–O bonds (green) and one short and one long Mn–O bond (blue). The fits and to the spectra at C0 and D3 are shown in (c) and (d), respectively. A comparison between the observed spectrum at C5 and simulated spectra for charge compensation schemes B and D (outlined in the text) is shown in (e).

^25^Mg NMR is challenging owing to the low natural abundance, low sensitivity and large quadrupole moment of the NMR-active ^25^Mg isotope. These challenges are exacerbated by the strong hyperfine interactions^74^ experienced by Mg^2+^ in NMMO, due to close proximity to open-shell Mn species (approximately 2.5 Å between Mn and Mg). To mitigate some of these difficulties, 100% ^25^Mg-enriched NMMO was synthesized, from which composite cathodes were prepared and cycled in half-cells. Owing to experimental constraints on NMR probes (no probe that could spin samples and tune down to the low frequencies required for ^25^Mg was available at the time of measurement) and on acquisition times, static Hahn-echo VOCS spectra were acquired (Figure 6).

Pristine NMMO exhibits a broad resonance spanning 6500–14 000 ppm (Figure 6a). Two sharp features are also observed and correspond to background resonances from the probe (due to the presence of Mg in the Macor stator; see SI, Section 3 and Figure S6). The width of the observed spectrum makes data interpretation challenging, as several models can reasonably fit the data.

The simplest model that can be used to fit the spectrum contains two sites described by a combination of quadrupolar interaction parameters and chemical shift anisotropy (the latter is a surrogate for the dipolar component of the hyperfine interaction). The fitted parameters, provided in Table 2, indicate that the two sites have isotropic shifts of 12 740 and 10 070 ppm for the pristine cathode (Figure 6a).

**Table 2:** Fitted NMR Parameters of the Simple Two-Site Model (Quadrupolar and Chemical Shift Anisotropy, CSA, Interactions Included) for the *Ex Situ*^25^Mg NMR Spectra of NMMO at C0 and D3^a^

	C0 (pristine)		D3	
	Site 1	Site 2	Site 1	Site 2
δ_iso_ (ppm)	12 740	10 070	12 250	10 070
Δδ_hyp_ (ppm)	2480	2610	2250	2050
η_hyp_	0.30	0.10	0.39	0.08
*C*_Q_ (MHz)	12.6	5.3	8.9	4.2
η_Q_	0.15	0.10	0.19	0.10
α (deg)	0*	0*	0*	0*
β (deg)	45*	45*	45*	45*
γ (deg)	26*	26*	26*	26*

a Values listed include: the isotropic chemical shift, δ_iso_, the dipolar hyperfine shift anisotropy, Δδ_hyp_, the asymmetry of the dipolar hyperfine tensor, η_hyp_, the quadrupolar coupling constant, *C*_Q_, the asymmetry of the electric field gradient tensor, η_Q_ and the Euler angles, α, β, and γ, between the dipolar hyperfine and quadrupolar tensors. Note that asterisks denote quantities which were fixed during fitting.

*Ab initio* calculations of the hyperfine and quadrupolar shifts of ^25^Mg environments in NMMO indicate that the 12 740 ppm resonance likely corresponds to Mg^2+^ centers with six Mn^4+^ nearest neighbors (with each Mn^4+^ contributing between 1700 and 1950 ppm to the hyperfine shift; as shown in Figure 6b,c), while the 10 070 ppm resonance corresponds to Mg^2+^ with five Mn^4+^ neighbors and one Mn^3+^ neighbor. We note that additional sites, for example due to Mg^2+^ centers with Mg^2+^ nearest neighbors, may also be present and contribute to the lower-frequency portion (intensity between 6500 and 8000 ppm) of the broad resonance observed experimentally. While a fit to a single environment can be performed (Figure S9) that is of similar quality, the extracted values are less physically reasonable compared to those extracted from the two-site model in Figure 6a. Furthermore, we anticipate that Mg^2+^ will experience multiple local environments, born out in our previous study where EPR revealed the presence of a Mn^4+^-only and a mixed Mn^3+^/Mn^4+^ environment. Our two-site model presented in Figure 6a is, therefore, the fewest number of sites that we might reasonably expect.

At the end of charge, a single resonance is observed around 5000 ppm, and no signal intensity remains between 6500 and 14 000 ppm, suggesting that the local environments of Mg have changed (Figure 6a,c). The width of this resonance and its proximity to the probe background make assignment difficult, with many models able to produce fits of similar quality.

The calculated NMR parameters for pristine NMMO and charge compensation schemes B and D are shown in Table 3. We suggest that scheme B is more consistent with the observed spectrum than D (Figure 6c), though some of the features in the spectrum of D may correspond to fast-relaxing species that cannot be observed. This is because the line width and shape of the resonance in B is more similar to the observed (experimental) spectrum than D; the distribution of resonances in D is also quite different from observation. All other schemes lead to shifts that are either too high or too low (Figure S8, Table S6). We note that the ^25^Mg shifts obtained for scheme E, where Mg^2+^ remains in the TMO_2_ layers, are far greater than those observed experimentally (Table S6, Figure S8), providing additional evidence for Mg^2+^ migration into the Na layers at high states of charge.

**Table 3:** Calculated ^25^Mg NMR Parameters for Pristine NMMO and NMMO under Charge Compensation States B and D, Using Two Levels of Hybrid Functionals, One with 35% Hartree–Fock Exchange (Hyb35) and One with 20% (Hyb20)^a^

	system	pristine	pristine	B	D	D	D
	number of Mn^4+^ n.n.	6	5	8	6	10	8
	number of Mn^3+^ n.n.	0	1	-	-	-	-
	number of O^*n*–^ n.n.	-	-	4 (*n* = 1)	-	-	-
	number of O_2_ n.n.	-	-	0	1	0	0
Hyb35	δ_hyp.,iso_ (ppm)	10 200	9310	3700	11 310	5410	3020
	Δδ_hyp_ (ppm)	1340	1427	–395	1459	–786	–844
	η_hyp_	0.08	0.1	0.27	0.08	0.12	0.28
	*C*_Q_ (MHz)	8.33	7.34	6.02	6.27	6.97	4.59
	δ_QIS_ (ppm)	–361	–265	–155	–193	–319	–100
	η_Q_	0.8	0.67	0.09	0.65	0.74	0.48
Hyb20	δ_hyp.,iso_ (ppm)	11 700	10 810	4450	13 360	6050	3620
	Δδ_hyp_ (ppm)	1337	1398	–395	1488	–777	–827
	η_hyp_	0.08	0.1	0.26	0.09	0.17	0.29
	*C*_Q_ (MHz)	8.05	7.3	5.84	6.38	6.96	4.66
	δ_QIS_ (ppm)	–332	–262	–147	–197	–317	–101
	η_Q_	0.77	0.66	0.08	0.63	0.76	0.47

a Calculations are performed assuming a field strength of 16.4 T and an experimental temperature of 300 K. Quantities listed include: the isotropic hyperfine shift, δ_hyp,iso_, the dipolar hyperfine shift anisotropy, Δδ_hyp_, the asymmetry of the dipolar hyperfine tensor, η_hyp_, the quadrupolar coupling constant, *C*_Q_, the second-order quadrupole-induced shift, δ_QIS_, and the asymmetry of the electric field gradient tensor, η_Q_.

The calculated shifts which most closely match those seen experimentally correspond to the following local environments: either Mg^2+^ bound to eight Mn^4+^ (or Mn^3.75+^) nearest neighbors and six “O^*n*–^” nest-nearest neighbors (B, *n* ∼ 1) or Mg^2+^ centers which sit furthest away from the trapped O_2_ (D). On the basis of this and of the relative energies of the different schemes, B is still the most likely mechanism.

The ^25^Mg NMR spectrum at D3 is similar to that for pristine NMMO; the simple two-site model used to fit the pristine NMMO spectrum can also be applied here, as expected from a (partially) reversible charge–discharge process (Figure 6d, Table 2). However, it is not possible to eliminate the possibility of residual Mg^2+^ in the Na layers, as indicated from ^23^Na NMR and XRD in our previous work.^42^ We note that the value of *C*_Q_ obtained for Mg in site 1 differs in the D3 and pristine spectral fits, which likely stems from a change in the covalency of the Mg–O bonds or a change to the position of Mg within its octahedral coordination shell in the TM layers, as *C*_Q_ is particularly sensitive to the local distribution of charges around the Mg nucleus.^91^ It may also arise from errors in fitting these spectra which do not have high signal-to-noise and are also poorly constrained due to overlapping resonances.

### *Ex Situ*^17^O NMR Spectroscopy

**Figure 7:**
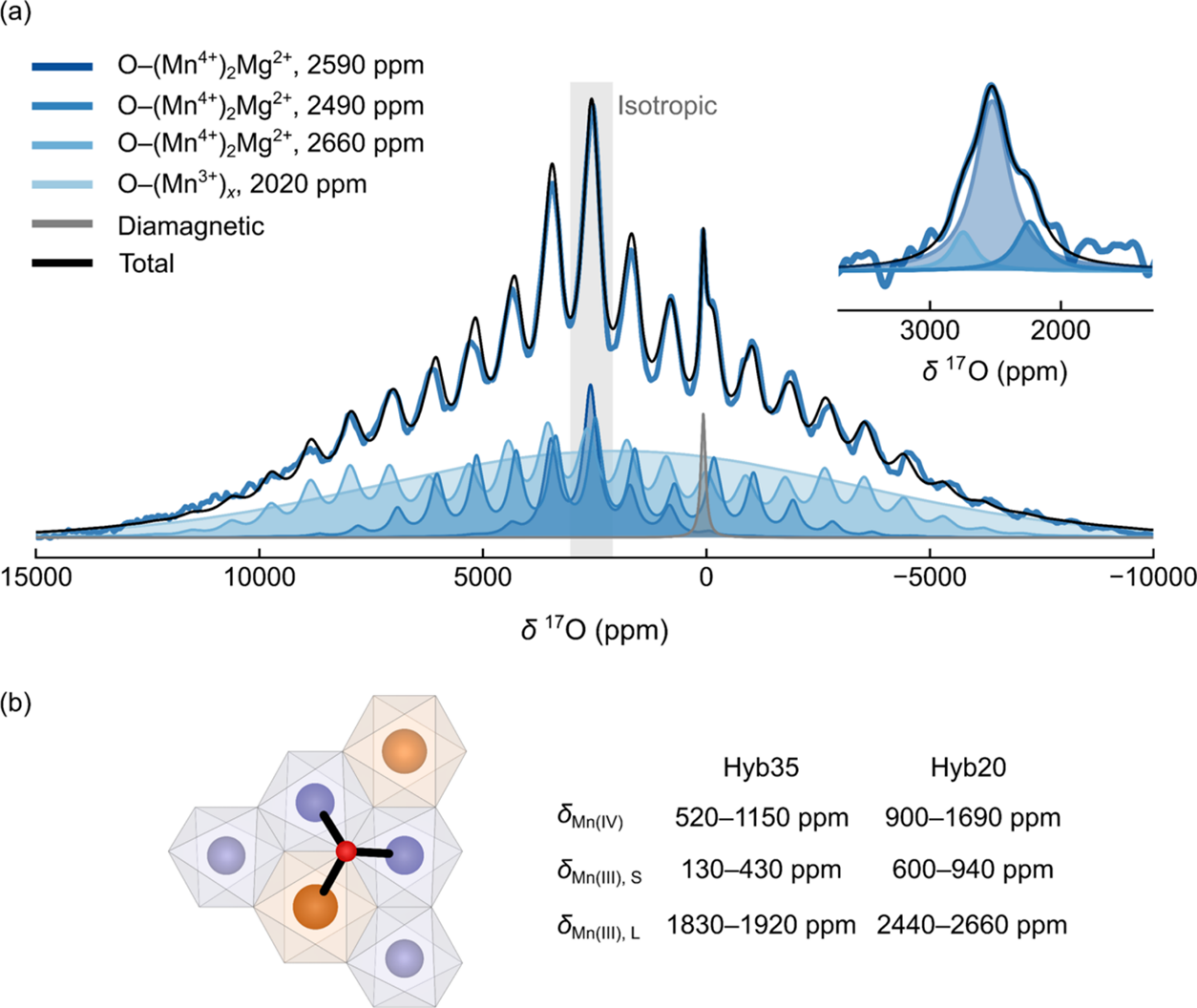
^17^O NMR spectroscopy of ^17^O-enriched pristine NMMO. In (a), the Hahn-echo VOCS data acquired (under 60 kHz MAS rate and a field of 11.7 T) is presented, with the main isotropic resonance highlighted. Inset shows the isotropic projection obtained from a pjMATPASS experiment. A fit to the pjMATPASS spectrum is also presented. In (b), the bond pathway hyperfine shifts obtained for ^17^O are shown; these used 35 and 20% Hartree–Fock hybrid functionals (Hyb35 and Hyb20, respectively), from which the peaks fit to the spectrum in (a) were assigned. The ranges quoted arise from the distributions in bond lengths and Mn–O–Mn angles.

**Figure 8:**
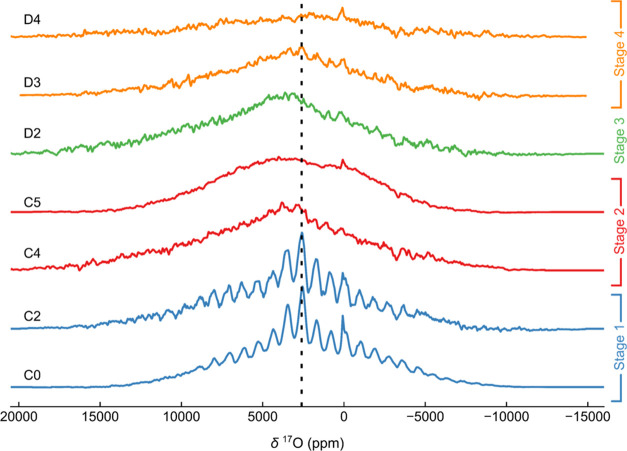
*Ex situ*^17^O NMR spectroscopy of ^17^O-enriched NMMO at 11.7 T and 60 kHz MAS. Note that the spectra are scaled to the number of scans and sample mass. The dashed line indicates the position of the isotropic resonance in pristine NMMO.

We next explore the local O environments and charge compensation mechanism in NMMO further using *ex situ*^17^O NMR spectroscopy (Figures 7 and 8). Strong electron–nuclear hyperfine interactions (due to short Mn–O distances), combined with the relatively strong quadrupolar interaction (*C*_Q_ ∼ 4 MHz) of ^17^O, yield a broad spinning sideband manifold spanning several thousand ppm, as has been observed in several LIB cathodes.^92^ For this reason, VOCS ^17^O NMR spectra were collected on each sample.

The pristine NMMO spectrum exhibits a single resonance at approximately 2490 ppm, associated with multiple sidebands (Figure 7a, Table 4). This resonance cannot be fit using a single tensor and at least three resonances are needed to fit the sharp sideband manifold. A fit is illustrated (Figure 7a) with three resonances with isotropic shifts of 2490, 2660, and 2590 ppm, these resonances corresponding to those identified in the projected magic-angle-turning phase-adjusted sideband separation, pjMATPASS, experiments^47^ (see inset to Figure 7a). A broad component—likely comprising a broad overlapping isotropic resonance and its individual sidebands—was also introduced, which is tentatively assigned to oxygen nearby Mn^3+^ ions. First-principles calculations of ^17^O NMR parameters on this system, including bond pathway analysis, reveal that Mn^4+^ centers bound to O^2–^ contribute a shift of between 520 and 1690 ppm per Mn^4+^ ion (Figure 7b, Table 5), the large range here again stemming from the two hybrid exchange–correlation functionals used. For each functional, however, a distribution of paths is observed; we attribute this to small differences in Mn–O–Mn local bond angles and lengths, consistent with pathways observed for Li_2_MnO_3_.^93^ Clearly, the resonances clustered around 2500 ppm in Figure 7a arise from O bound to two Mn^4+^ centers and are similar to those observed in ^17^O NMR spectra obtained on Li_2_MnO_3_. Additional fits using only two or three sites suggest this four-site model is the minimum number of components required to adequately fit the spectrum. Since pristine NMMO is known to adopt a mixture of both a disordered parent phase with one crystallographically unique O position and an ordered daughter phase with four O positions, a four-component fit is reasonable. However, the values extracted should only be taken as ranges of quadrupolar coupling constants and hyperfine interactions for the environments in this material.

**Table 4:** Fitted ^17^O Hyperfine and Quadrupolar Parameters from the Fit to the ^17^O NMR Spectrum of Pristine NMMO at 11.7 T and 60 kHz MAS^a^

site	δ_iso_ (ppm)	Δδ_hyp._ (ppm)	η_hyp._	*C*_Q_ (MHz)	η_Q_
O–(Mn^4+^)_2_Mg^2+^	2490(30)	–5210(50)	0.85(2)	3.57(3)	0.48(2)
O–(Mn^4+^)_2_Mg^2+^	2660(20)	–8140(20)	0.91(2)	3.65(2)	0.30(3)
O–(Mn^4+^)_2_Mg^2+^	2590(20)	–2030(25)	0.07(2)	3.48(3)	0.60(2)
O–(Mn^3+^)_*x*_	2020(350)				

a Note that all sites except the O–(Mn^3+^)_*x*_ site were fit using combined chemical shift anisotropy and quadrupolar models; the O–(Mn^3+^)_*x*_ site was fit to a Gaussian/Lorentzian peak shape and as such we report only the center-of-mass of this signal. Values listed include: the isotropic hyperfine shift, δ_hyp,iso_, the dipolar hyperfine shift anisotropy, Δδ_hyp_, the asymmetry of the dipolar hyperfine tensor, η_hyp_, the quadrupolar coupling constant, *C*_Q_, and the asymmetry of the electric field gradient tensor, η_Q_.

**Table 5:** Calculated ^17^O NMR Parameters for Pristine NMMO Using Two Levels of Hybrid Functionals, One with 35% Hartree–Fock Exchange (Hyb35) and One with 20% (Hyb20)^a^

		O–(Mn^4+^)_2_Mg^2+^	O–Mn^3+^Mn^4+^Mg^2+^
Hyb35	Δ*δ*_hyp_ (ppm)	–4785	2034
	η_hyp_	0.407	0.593
	*C*_Q_ (MHz)	3.84	3.6
	δ_QIS_ (ppm)	–16	–30
	η_Q_	0.840	0.519
Hyb20	Δδ_hyp_ (ppm)	–4574	792
	η_hyp_	0.394	0.603
	*C*_Q_ (MHz)	3.57	3.4
	δ_QIS_ (ppm)	–14	–26
	η_Q_	0.800	0.492

a Calculations are performed assuming a field strength of 11.7 T and an experimental temperature of 318 K (60 kHz MAS). Quantities listed include: the dipolar hyperfine shift anisotropy, Δδ_hyp_, the asymmetry of the dipolar hyperfine tensor, η_hyp_, the quadrupolar coupling constant, *C*_Q_, the second-order quadrupole-induced shift, δ_QIS_, and the asymmetry of the electric field gradient tensor, η_Q_.

Underneath the sharp ^17^O resonances lies an extremely broad resonance centered at approximately 2020 ppm, ascribed to O bound to one or more Mn^3+^ centers. Bond pathway analysis of O environments bound to Jahn–Teller (JT)-distorted Mn^3+^ indicate that short Mn–O bonds contribute a shift of between 130 and 940 ppm, while long Mn–O bonds contribute between 1830 and 2660 ppm (Figure 7b). If the JT elongated axis of Mn^3+^ fluctuates on the NMR time scale, O bound to Mn^3+^ will experience a large change in resonant frequency, resulting in rapid transverse signal dephasing (hence a shortened *T*_2_) and a severely broadened resonance; this effect has also been observed in ^17^O NMR spectra of Li[Ni_0.8_Co_0.15_Al_0.05_]O_2_ materials.^92^ Identifying how many Mn^3+^ centers are bound to O is challenging, owing to the width of this resonance. Were the Mn^3+^ centers to be statically JT-distorted, the local moment around O would change far less, likely resulting in sharper, more well-resolved resonances, as seen for JT-distorted Ni^3+^ in La_2–*x*_Sr_*x*_NiO_4+δ_.^48^ The presence of this broad feature therefore suggests that the JT distortion in NMMO is dynamic, rather than static. We note however the errors associated with the analysis of this broad resonance, since severe overlap of multiple sidebands can often be modeled by adding a broad resonance. The center of mass of this resonance is different from that of the sharper components, indicating that this resonance is real.

A sharp resonance at approximately 72 ppm was also observed in all of the ^17^O spectra and can be assigned to diamagnetic ^17^O species, e.g., carbonates and hydroxyls on the surface of the NMMO particles (Figure 7a), due to the presence of residual precursors (pristine sample) and/or electrolyte decomposition products (cycled samples).^94,95^

Quadrupolar parameters for the sharp environments near 2500 ppm were also obtained from a fit of the observed spectrum. The *C*_Q_s for each of the O sites, approximately 3.5 MHz, are consistent with calculated values (between 3.6 and 3.8 MHz; Tables 5 and S5 and S6) and with fits of spectra acquired at a different magnetic field strength (Table S9, Figure S11). Since the *C*_Q_ value is related to the degree of ionicity or covalency of the local ^17^O environment,^96^ with small *C*_Q_s corresponding to high ionic character and large *C*_Q_s to high covalent character, a *C*_Q_ value of 3.5 MHz suggests a relatively ionic bond (approximately 60%, according to ref (96)), as expected for Mn^4+^ bound to O^2–^.

On charging NMMO through stage 1, minor changes are observed in the ^17^O NMR data, with only a small increase (*ca*. 50 ppm) in the shift of the NMMO cathode signal (Figure 8). This increase in the Fermi contact shift likely arises from a change in the O–Mn bond lengths and Mn–O–Mn bond angles, as expected from the oxidation of the few JT-distorted Mn^3+^ centers in the pristine cathode to Mn^4+^.^97^ In addition to the shift increase, the very broad and underlying resonance previously ascribed to O bound to Mn^3+^ decreases in intensity.

During stage 2, the well-defined, relatively sharp resonances observed for the pristine (C0) and C2 samples become less well-defined and are replaced by a broad feature with a center-of-mass at approximately 3800 ppm for the C4 and C5 samples (Figure 8).

**Figure 9:**
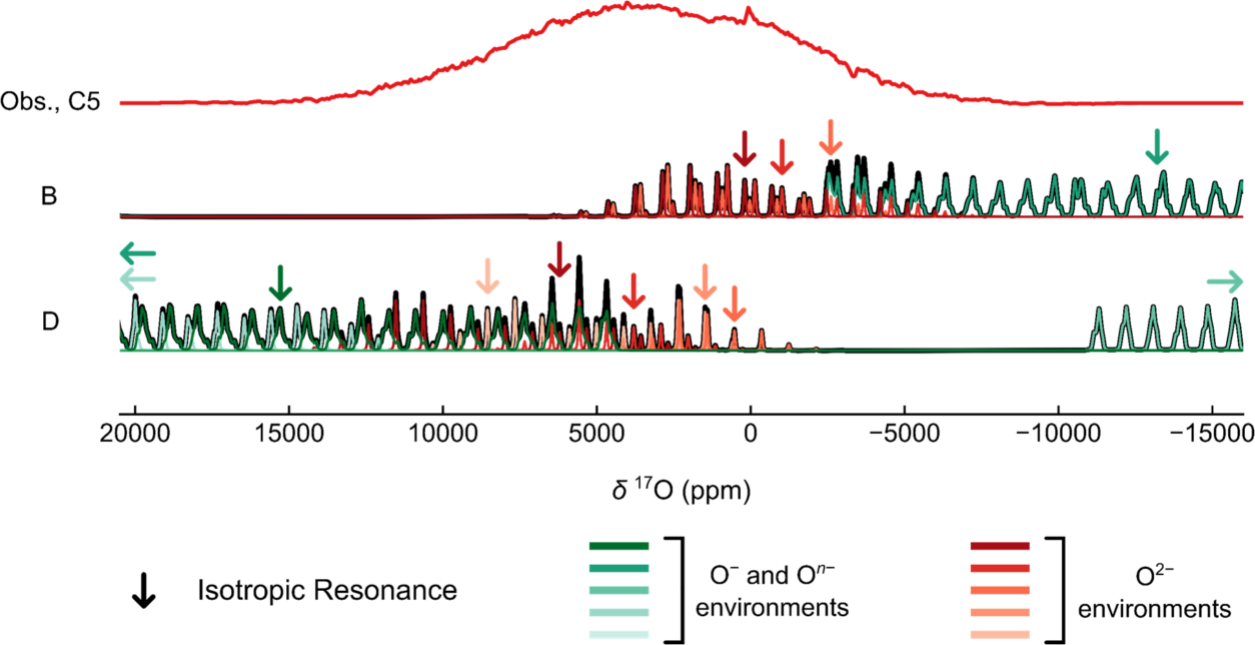
Comparison of the *ex situ*^17^O NMR spectrum acquired at point C5 of ^17^O-enriched NMMO (at 11.7 T and 60 kHz MAS) with simulated spectra for charge compensation mechanisms B and D. Arrows which face one side or the other in spectrum D indicate isotropic resonances beyond the plot window shown here.

Simulations of the ^17^O NMR spectrum at point C5 were performed using the parameters computed for charge compensation scenarios B and D (Figure 9; simulations from A, C, and E are in the SI, Figure S13). O^*n*–^ and O_2_ environments appear at either large positive shifts (*ca*. 16 000 to 20 000 ppm for schemes A, C, and D) or large negative shifts (*ca*. −25 000 to −12 000 ppm for schemes B, C, D, and E). No such resonances are observed experimentally, consistent with the paramagnetic nature of O^*n*–^ (*n* ∼ 1) and (O_2_)^*m*−^ species (0 < *m* < 2), which results in very rapid nuclear relaxation times and complete signal decay by the time the data is recorded. The only simulated resonances that agree well with observation correspond to O environments with very little unpaired electron density at the nuclear position (i.e., O^2–^ interacting with nearby paramagnetic O^–^, with O_2_-like species and/or with Mn^4+^). Assignment of the spectrum to specific local environments, however, is not possible, as both B and D have at least one set of resonances consistent with the observed spectrum. We note that, unlike previous work, we see no evidence of sharp resonances corresponding to trapped O_2_ in the ^17^O NMR spectra,^15,98^ at either the previously reported shifts (approximately 3000–3300 ppm at 318 K and 7.05 T) or Curie–Weiss scaled shifts of liquid ^17^O_2_ (approximately 27 500 ppm at 318 K and 7.05 T).^99^

Discharging NMMO to point D2 yields a spectrum similar to C4, indicating that O environments similar to those present during charge are regenerated on discharge (Figure 8). At point D3, the spectrum remains severely broadened, but a relatively sharp, low-intensity resonance appears at approximately 2600 ppm. This resonance is attributed to O^2–^ bound to Mn^4+^; the small change in the isotropic shift compared to the resonance observed for pristine NMMO likely stems from slight changes in the bond lengths and bond angles around ^17^O. The resonance observed for the D3 sample is at a slightly higher shift (center-of-mass at approximately 3000 ppm in D3, compared to 2500–2600 ppm in C0) and is much broader than that observed for the pristine material, suggesting a broader distribution of bond lengths and angles that leads to greater overlap of closely spaced resonances. At the end of the first discharge, the overall signal intensity decreases and the distinct feature present at D3 disappears. We attribute the decrease in intensity to two effects: first, the larger number of Mn^3+^ centers in NMMO, leading to shorter spin–spin relaxation (*T*_2_) times, and therefore loss of ^17^O signal intensity and signal broadening; second, a loss of ^17^O species at or near the surface of the particles. The latter was confirmed by the increase in signal intensity between the pristine state and end of first change in solution state NMR, where we compared the ^17^O signal intensity of unenriched electrolyte when cycled with an unenriched and a ^17^O-enriched cathode (Figure S16). We expect the former dominates over the latter, as only a small increase (1.4 times) in ^17^O NMR signal was seen in solution state NMR between the electrolytes of half cells with enriched and unenriched cathodes, while the solid-state NMR intensity at D3 is approximately half that of pristine NMMO.

The ^17^O NMR results indicate a large change in the local environments of O species during charge, with O going from well-defined environments in the pristine material and during stage 1 on charge, to a severely broadened resonance in stage 2 (top of charge). This broadening likely stems from rapid nuclear relaxation times and/or a broad distribution of local environments. To interrogate the local environment of the electronic spins in NMMO, we performed bulk magnetic susceptibility, *operando* X-band electron paramagnetic resonance (EPR) spectroscopy and *ex situ* high-frequency EPR.

### Bulk Magnetic Susceptibility

**Figure 10:**
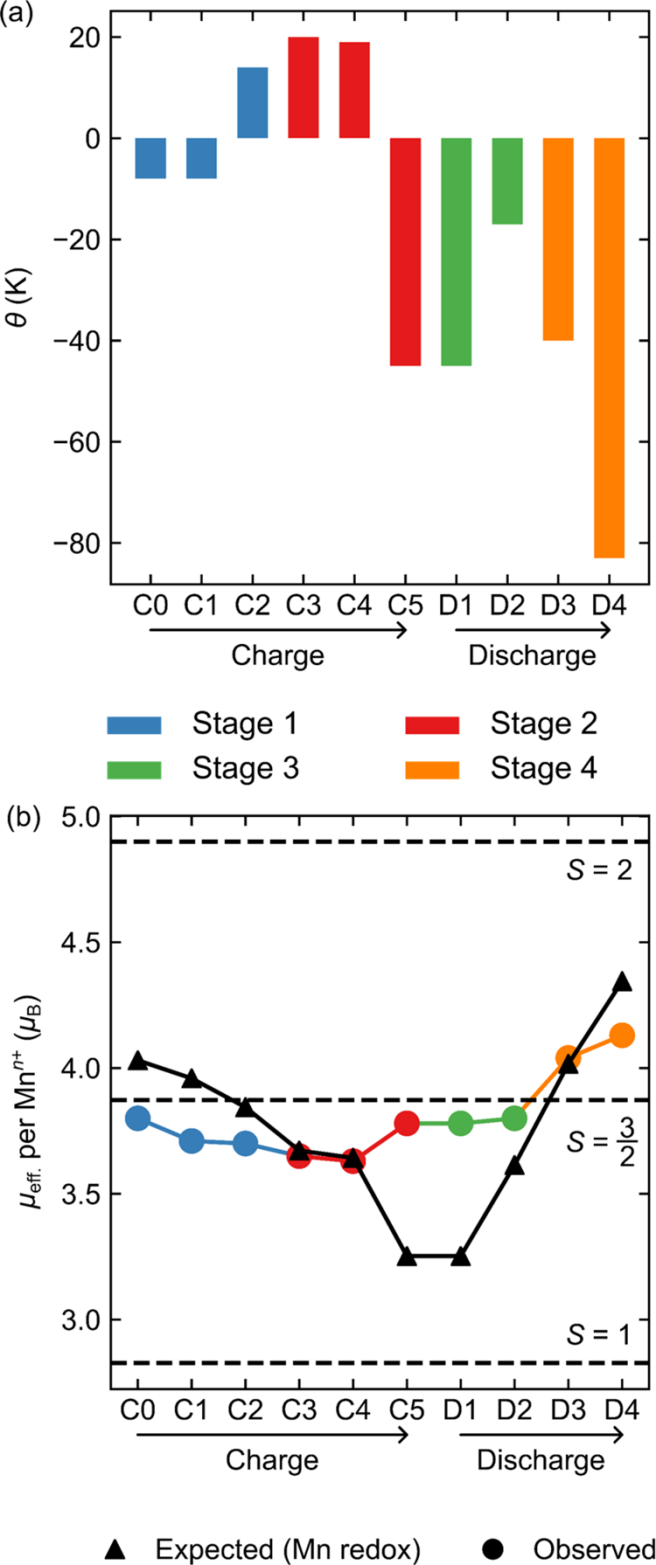
Extracted Curie–Weiss parameters obtained from *ex situ* bulk magnetic susceptibility data collected for NMMO under a field of 0.1 T: (a) shows the Weiss constant, *θ*, while (b) shows the effective magnetic moment per mole of Mn in NMMO, *μ*_eff_, with the predicted μ_eff_ for Mn-only redox shown in black. The error in *θ* is 1 K, while the error in *μ*_eff_ is 0.04 μ_B_ per Mn; the error bar in θ is omitted in (a) for clarity and for *μ*_eff_ is represented by the marker size in (b).

Bulk magnetic susceptibility measurements on *ex situ* samples of NMMO were carried out at a field of *H* = 0.1 T. In addition, isothermal magnetization measurements (*M*(*H*) curves) were carried out between 2 and 300 K (Figures S14 and S15) and indicate that saturation is never achieved in the region −7 to +7 T. For each sample, high-temperature magnetic susceptibility (*T* > 150 K) data were fitted to the Curie–Weiss law, yielding the Weiss constants, *θ*, and effective magnetic moments, *μ*_eff_, shown in Figure 10.

All samples displayed spin-glass behavior, as indicated by the frequency-dependent peak in the AC susceptibility for pristine NMMO (Figure S19), similar to other layered cathode materials.^100^ This behavior is attributed to competing intralayer exchange interactions between Mn^3+^ and Mn^4+^ in NMMO: the Mn^3+^–Mn^3+^ interaction is anticipated to be antiferromagnetic and strong, while the Mn^3+^–Mn^4+^ and Mn^4+^–Mn^4+^ interactions are expected to be either weakly ferromagnetic or weakly antiferromagnetic, owing to the competing ferromagnetic superexchange (from *t*_2g_ to *t*_2g_*via* intervening O 2*p* orbitals) and antiferromagnetic direct exchange interactions (Figure S20). Although strongest, Mn^3+^–Mn^3+^ interactions only just compensate the weak net Mn^3+^–Mn^4+^ and Mn^4+^–Mn^4+^ interactions owing to the low probability of finding two neighboring Mn^3+^ centers in pristine NMMO. Besides causing spin-glass behavior, competing ferromagnetic and antiferromagnetic interactions result in an unusually small Weiss constant (compared to other oxide-based framework materials) of −10 K.

On charging NMMO through stage 1, the Weiss constant goes from weakly antiferromagnetic (−10 K at C0) to weakly ferromagnetic (+14 K at C2) due to the oxidation of Mn^3+^, and dominant ferromagnetic interactions between Mn^4+^ centers (Figure 10a). Further oxidation along stage 2 results in a small initial increase in the Weiss constant—which likely stems from oxidation of any residual Mn^3+^ centers—followed by a sharp decrease at the end of charge (point C5). This rapid decrease suggests a new strong antiferromagnetic exchange interaction in NMMO, which we tentatively attribute with the strong exchange coupling between Mn^4+^ and O^–^ ions (−500 K) discussed earlier for charge compensation scenario B.

On discharge, *θ* becomes more positive initially, and then more negative. The sharp decrease in *θ* to more negative values observed at the end of discharge stems from reduction of Mn^4+^ to Mn^3+^. At D3, *θ* does not return to the value obtained for the pristine NMMO sample, despite having the same nominal Na^+^ content, suggesting a significant change in the electronic structure between charge and discharge (and consistent with the ^17^O NMR results, where the spectrum at D3 is significantly different to C0). This could be related to residual Mg^2+^ in the Na^+^ layer on discharge (see Discussion).

The effective magnetic moment, *μ*_eff_, also provides useful information on the electronic structure of the cathode (Figure 10b). Pristine NMMO exhibits an effective magnetic moment of 3.80 *μ*_B_ per Mn center, below the spin-only (SO) value based on the average Mn oxidation state (*μ*_SO_(Mn^3.85+)^ = 4.05 *μ*_B_). This discrepancy originates from deviation of the *g*-factor from the free-electron value *g*_e_ ≈ 2 (see EPR results) for both Mn^3+^ and Mn^4+^ centers, a consequence of spin–orbit coupling, delocalization of electron spin density from Mn centers to nearby species (Na, Mg, and O) and possible temperature-dependence of the Curie constant.^101−103^ For comparison, the spin-only moments expected at each state of charge based on Mn-only redox have been plotted in Figure 10b.

During stage 1 on charge, *μ*_eff_ decreases due to oxidation of Mn^3+^, which decreases the net spin per Mn^*n*+^ center. On further charging through stage 2, *μ*_eff_ continues to decrease. At the very end of charge, *μ*_eff_ increases back to the moment obtained for pristine NMMO. The small decrease in *μ*_eff_ seen along the charge plateau suggests a gradual change in the electronic structure and a sudden change at the end of charge. The expected spin-only moments for B and D are 4.24 and 4.15 *μ*_B_ per Mn center, respectively, neither of which match well with experiment. This is explored further in the discussion, but we speculate that this stems from a deviation from the spin-only moment and interactions between Mn and O spins.

On discharge, *μ*_eff_ remains approximately constant from C5 to D2. While there is little change in the Na^+^ content (and therefore in the average Mn oxidation state) from C5 to D1, by point D2 approximately 0.3 equiv of Na^+^ have been reinserted and the lack of change in the moment is surprising. One possibility is the presence of competing effects: if, for example, Mn and O are both reduced, this would cause the moment on Mn to increase, but the moment on O to decrease; this scenario is explored further in the Discussion section. At the end of discharge, *μ*_eff_ increases sharply, as anticipated for Mn^4+^ reduction back to Mn^3+^. The deviation from the spin-only moment arises from the large spin–orbit coupling expected for Mn^3+^ (*E*_g_ ground term). The increase in *μ*_eff_ between C0 and D3 (approximately the same Na composition) indicates a larger number of unpaired spins at D3, perhaps due to some residual spin on O at point D3. This is explored further in the discussion.

### *Operando* EPR Spectroscopy

**Figure 11:**
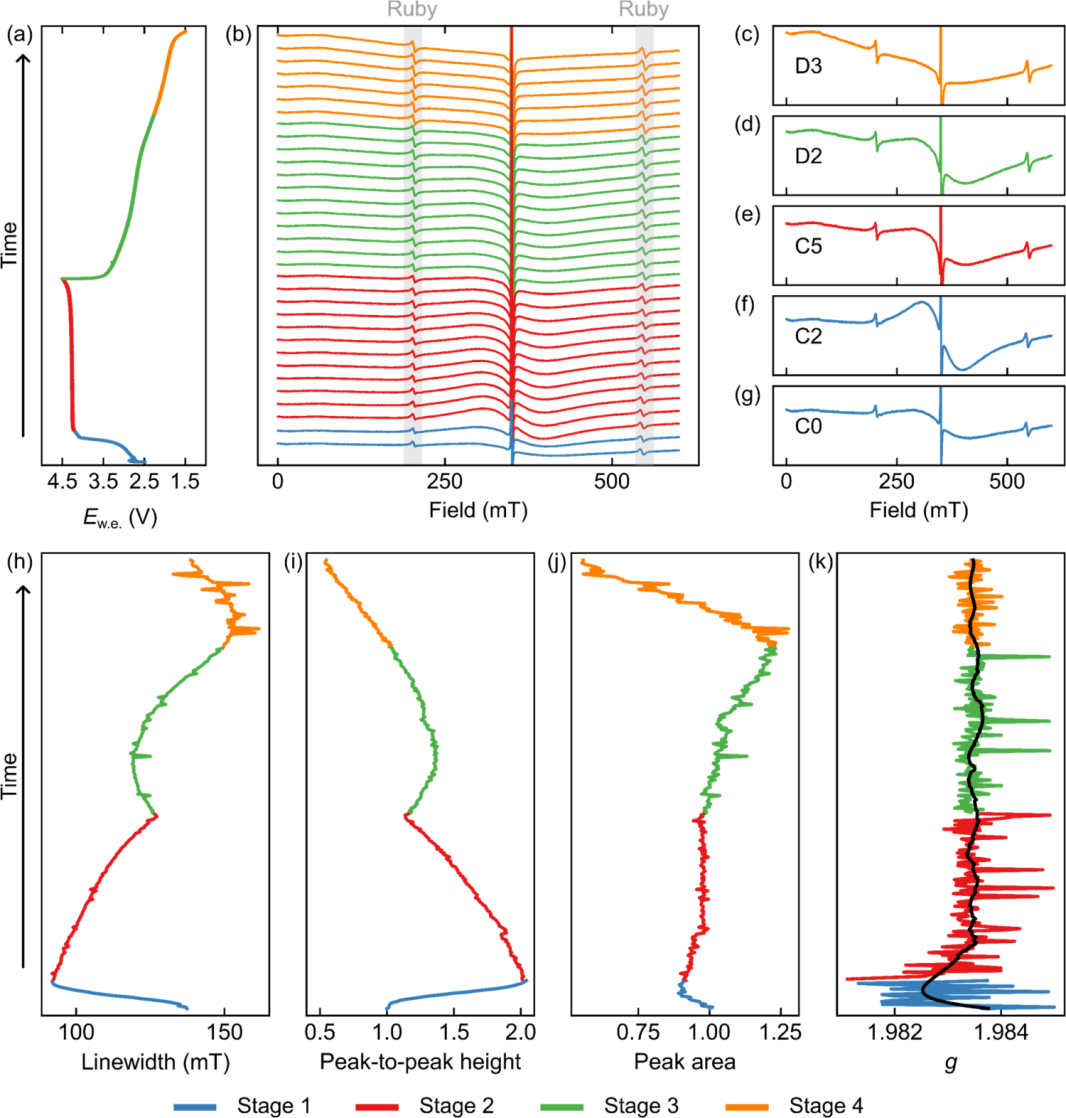
*Operando* X-band EPR of an NMMO/Na metal half cell during the first charge–discharge cycle, cycled at a rate of 10 mA g^–1^. In (a), the voltage profile for the half cell is shown, with every fifth spectrum along the profile shown in (b), and the ruby resonances highlighted in gray. Individual spectra at the voltages corresponding to *ex situ* points C0, C2, C5, D2, and D3 are shown in (c) to (g), respectively; note that the *y*-axis scale is the same on all these plots. Panels (h), (i), (j), and (k) show the fitted line widths, extracted peak-to-peak heights, total peak areas and the fitted isotropic *g*-values plotted as a function of time along the charge–discharge cycle, respectively. The peak-to-peak height and peak are have been normalized relative to the pristine material. The black trace in (k) corresponds to the smoothed *g*-factor; a Savitzky–Golay filter was applied to the data to carry out the smoothing.

To further investigate the local distribution of electron spins and their evolution during charge and discharge, *operando* X-band EPR was performed on a half-cell of NMMO *vs* Na metal (Figure 11).

Three noticeable features are evident from the EPR spectra: (1) two sharp, low-intensity features at 203 mT (effective *g* = 3.447) and 545 mT (effective *g* = 1.286), corresponding to the ruby reference; (2) a broad resonance centered at 340 mT spanning at least 250 mT, corresponding to the cathode signal and (3) a sharp, high-intensity resonance centered at 350 mT (*g* = 1.999), corresponding to conducting electrons in the carbon additive (part of the cathode composite) and the Na metal counter electrode (Figure 11b). The spectrum exhibits a severe background arising from the current collectors and wires that has been subtracted from the spectra (Figure S22). However, this subtraction is not perfect, due to changes in the background signal during charging (potentially due to the changing susceptibility of the cell).

The spectra were fit using the methodology explained in the SI (Section S1). The following discussion focuses on changes to the broad resonance associated with the cathode material. The pristine cathode resonance arises due to Mn^4+^ species in the material (the strong zero-field splitting prevents the observation of Mn^3+^ species at X-band microwave frequencies, ca. 9 GHz). The signal is extremely broad, spanning several hundred mT, a result of the strong, spatially anisotropic dipolar interactions between Mn^3+^ and Mn^4+^ spins, as well as the distribution of Mn environments.^43^ On charge, transitions involving other spin microstates (delocalized states, O^*n*–^ and (O_2_)^*m*−^ species) are expected to be observed, but their lineshapes and *g*-factors cannot be predicted *a priori*. The peak-to-peak line width, Δ*H*_pp_, depends on the strength of the dipolar and magnetic exchange interactions—the former generally broadens spectra, while the latter narrows them—as well as on the distribution of local environments. The *g*-factor is analogous to the chemical shift in NMR and is diagnostic of the local chemical and magnetic environment of the (here, electronic) spin under observation. Deviation of *g* from the free electron value (*g*_e_ ≈ 2.0023) arises from spin–orbit coupling, the size of the deviation depending on the strength of spin–orbit coupling and hence the difference between the ground and excited states, as well as the size of the orbital spin–orbit coupling constant.^104^ Residual magnetic interactions may also result in further deviations (see below). The peak-to-peak height and signal intensity may be combined to give a peak area (intensity multiplied by Δ*H*_pp_^2^), which is related to the static susceptibility of the sample.

Throughout stage 1, the line width decreases (Figure 11h) while the peak-to-peak height increases (Figure 11i), corresponding to a decrease in peak area (Figure 11j) and therefore susceptibility. Those changes are attributed to the loss of Mn^3+^, resulting in a narrower distribution of Mn^4+^ local environments, as well as weaker dipolar interactions (the strength of the dipolar coupling depends on, among other factors, the number of unpaired electrons (or *S*), which is larger for Mn^3+^ than for Mn^4+^). Simultaneously, the *g* factor decreases from approximately 1.984 to 1.983, which, while a seemingly small difference, represents a large change in the local environment (Figure 11k). This drop in *g* (away from *g*_e_) is consistent with *g*-factors reported in the literature^101,105,106^ and likely stems from the change in local exchange interactions, as the nearby Mn^3+^ ions are oxidized, altering the local field experienced by electrons and therefore their *g*-factors.^107,108^

On charging through stage 2, the increase in line width suggests either a broader distribution of local environments, an increase in the electron–electron dipolar coupling strength and/or a decrease in magnetic exchange interaction strength, while the decrease in peak-to-peak height under an approximately constant peak area indicates a decrease in the number of spin microstates that can be excited. The initial increase in *g* (toward *g*_e_) suggests a decrease in the spin–orbit coupling interaction strength, which could arise from forming delocalized states between Mn and O. This increase in *g*, followed by a constant *g* over the high voltage plateau region, is explored further in the Discussion section.

During stage 3 on discharge, the line width initially decreases and the peak-to-peak height increases, leading to a gradual increase in peak area. This is followed by an increase in the line width and a decrease in the peak-to-peak height, giving a faster rise in peak area. The initial decrease in line width suggests a narrower distribution of environments (consistent with the increase in peak-to-peak height), a decrease in the dipolar coupling strength and/or an increase in magnetic exchange interaction strength. The increase in line width partway through stage 3 suggests stronger dipolar coupling, likely due to the formation of Mn^3+^ species, while the concomitant increase in peak area corresponds to an increase in static susceptibility (Figure 11h,I,j), suggesting an increase in the number of unpaired electrons.

At stage 4, the line width falls, while the peak-to-peak height continues to decrease. The former suggests a smaller distribution of local environments, while the latter likely arises from the loss of X-band EPR-active Mn^4+^ centers (perhaps *via* oxidation of Mn^4+^ to Mn^3+^); this is also reflected in the decrease in peak area. Throughout discharge, *g* remains essentially constant, surprising given the significant change in Na content on discharge (from 0.10 equiv Na to 0.89 equiv Na). It is also noteworthy that the signal seen for pristine NMMO is not recovered, which we suggest is consistent with the formation of delocalized states between Mn and O. Independent of the mechanism, there is a consistent change in structure between the pristine sample and D3.

### *Ex Situ* High-Frequency EPR Spectroscopy

**Figure 12:**
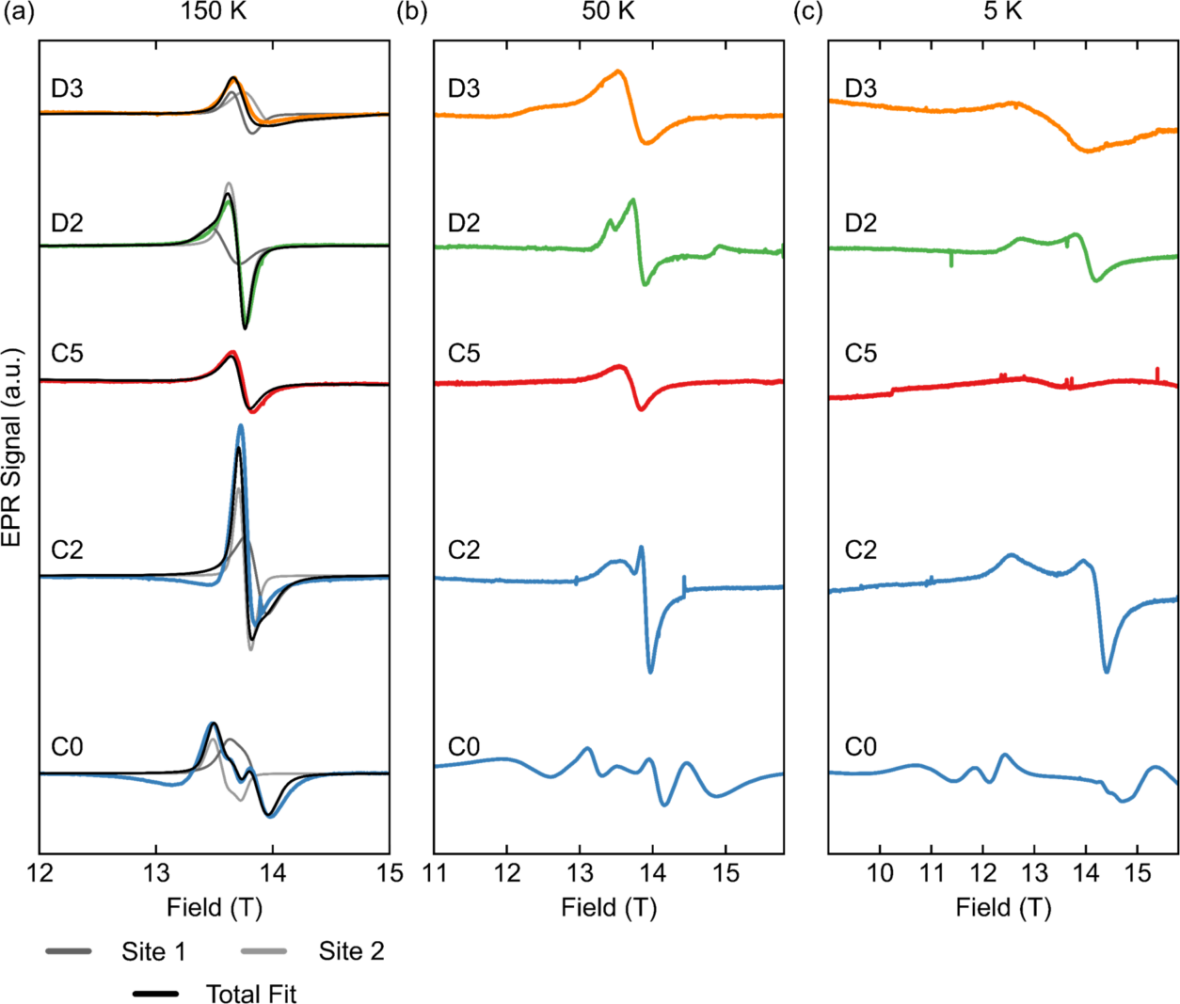
*Ex situ* high-frequency EPR of NMMO, recorded at 383.04 GHz microwave frequency. Spectra have been rephased using a spherical harmonic function and are plotted scaled by the sample mass. In (a), the spectra at 150 K are shown alongside fits; (b) and (c) show spectra recorded at 50 and 5 K, respectively.

X-band EPR, while beneficial for providing information about the electronic structure of “dilute” paramagnetic systems, is resolution-limited for “concentrated” paramagnetic systems such as those studied here, due to the severe line broadening induced by the strong electron–electron dipolar interactions. Hence, variable-temperature EPR spectra were acquired on *ex situ* cathode samples at extremely high frequencies (>200 GHz) to maximally resolve overlapping resonances and obtain additional information on the exchange interactions involving the different electron spin environments (Figure 12). By varying the temperature, both the paramagnetic (Curie–Weiss) regime, where EPR probes each spin individually, and the magnetically ordered regime, where EPR probes spin ensembles, can be studied.

The microwave phase in high-frequency EPR (HF-EPR) differs from “conventional” (low-frequency) EPR, owing to the spectrometer design to allow broad frequency operation (*i.e.*, the signal is simply the light transmitted through the sample that arrives at the bolometer).^109^ To assist interpretation, each spectrum was phased using a spherical harmonic function to minimize the out-of-phase component of the spectrum. The low-temperature spectra span a large field range, consistent with strongly coupled spin systems. Since the phase may vary in a nonlinear way across these spectra, making phasing and fitting challenging, only the highest temperature spectra (*T* = 150 K) for each *ex situ* sample was fitted. The fitted *g*-values are given in Table 6. We note these values differ in part from those observed at X-band, a consequence of the offset between the true field at the sample and the requested set field, generated from the large inductance built up from sweeping across large magnetic field ranges. Qualitative assignments of the full set of spectra can be made based on the temperature behavior of the signals. The changes in *g* as a function of temperature (caused by, for example, slowing down of the magnetic fluctuations) and microwave frequency will be the focus of a future study.

**Table 6:** Fitted High-Frequency EPR *g*-Values: *g*_*x*,*y*_ Represents the In-Plane Component of the *g*-Tensor and *g*_*z*_ the Axial Component^a^

sample	*g*_*x*,*y*,1_	*g*_*z*,1_	*g*_iso,1_	*g*_*x*,*y*,2_	*g*_*z*,2_	*g*_iso,2_
C0	2.024(16)	1.991(2)	2.013(2)	1.963(19)	2.008(4)	1.978(3)
C2	1.989(4)	1.978(12)	1.985(8)	1.971(10)	1.970(6)	1.971(5)
C5			1.992(3)			
D2	1.991(5)	2.011(4)	1.998(5)	2.007(13)	2.027(7)	2.014(4)
D3	1.97(3)	1.90(9)	1.95(6)	1.98(2)	2.00(2)	1.99(2)

a For samples C0, C2, D2, and D3, two axial resonances were used to model the spectrum; for comparison, the isotropic *g*-values were computed *viag*_iso_ = (2*g*_*x*,*y*_ + *g*_*z*_)/3. At C5, the low intensity and breadth of the resonance could only reasonably be fit to one, isotropic resonance. The strain (approximate error bar) in each *g*-value is shown in parentheses.

It is anticipated that the HF-EPR spectra probe Mn^4+^ environments only, based on the spectra acquired for NaMnO_2_, an ordered, Mn^3+^-only system, where no HF-EPR resonances could be observed. Parallel-mode (X-band) EPR spectra were also recorded to try and excite Mn^3+^-based transitions, but no additional resonances were observed. This is likely due to a combination of a large Mn^3+^ zero field splitting interaction and insufficient distortion of the local Mn^3+^ coordination environment (to enable the mixing of ground and excited states required to observe the formally forbidden integer-spin transitions). We note that hyperfine couplings to ^17^O are not observed (Figure S21); our calculations suggest a splitting of between −0.43 and −0.14 mT in the pristine material and between −0.18 to +1.6 mT for the antiferromagnetically coupled state and −5.4 to +3.2 mT for the trapped O_2_ state at the end of charge. These are much smaller than the width of the observed lines; while such couplings may be present, obtaining a meaningful value of the hyperfine splitting by adding this to our fits would be very challenging.

Beginning with the high-temperature (150 K) spectra, two overlapping resonances are observed for pristine NMMO (C0), and for sample C2, D2, and D3 and can be fitted with two axial *g*-tensors (Figure 12a, Table 6). Axial *g*-tensors are expected for layered Mn^4+^-containing systems, as the in-plane component, *g*_*x*,*y*_, will differ from the out-of-plane component, *g*_*z*_, by virtue of the near-axial symmetry around Mn. Yet, the spectrum at C5 could only be fitted to a single, isotropic resonance, suggesting a significant change in the local environment of the unpaired spins.

The *g*-factors for the two NMMO signals decrease between C0 and C2, then slightly increase at C5 and decrease again at D2 and D3, with the values at D2 and D3 lower than those observed in the pristine material. On the basis of empirical observations by Stoyanova *et al*., whereby sites with stronger net exchange coupling interactions have larger deviations of *g* from *g*_e_,^110^ the lower *g* resonance in C0 is tentatively assigned to Mn^4+^ with only Mn^4+^ and Mg^2+^ nearest neighbors, while the higher *g* resonance is assigned to Mn^4+^ with Mn^4+^, Mn^3+^ and Mg^2+^ nearest neighbors. The decrease in both *g*-factors from C0 to C2 is in line with the *operando* measurements and is attributed to Mn^3+^ oxidation, while the increase between C2 and C5 is again consistent with the *operando* data and suggests a decrease in spin–orbit coupling that could be due to the formation of delocalized Mn–O states. This scenario is explored further in the discussion.

The increase in *g* on discharge differs from the *operando* data fits, where a flat trend in *g* on discharge was observed. However, it is also noted that size of the distribution of the *g*-tensor components (the “strain” in *g*) at points D2 and D3 are far greater than those at C0, C2, or C5, suggesting a much broader distribution of environments for the paramagnetic species. Therefore, it is possible that the *operando* X-band spectra capture less information in the limit of a broad distribution of environments; it will, however, better capture metastable states not seen in *ex situ* measurements. Compared to C5, the *g* values increase at D2, suggesting a further decrease in spin–orbit coupling experienced by the unpaired electron. Discharging to D3 results in a decrease in *g* back to values consistent with those observed in the pristine material, indicating that the unpaired electrons sit primarily on Mn^4+^ centers. Complete assignment of these spectra require *ab initio* calculations of the *g*-factors, but such calculations are challenging in concentrated paramagnetic systems and beyond the scope of this work.

On cooling to 50 K, the exchange interaction begins to dominate the spectrum, indicated by the spreading of resonances to high and low fields. The spectrum of pristine NMMO splits into (at least) five overlapping resonances, while the spectra at points C2, D2, and D3 split into at least two resonances, the latter further suggesting axially symmetric *g*-tensors. At point C5, an isotropic resonance is still observed, but the signal intensity drops significantly compared to the 140 K spectrum (Figure 12c). At this stage, it is anticipated that each sample is starting to form spin clusters, with some of the resonances remaining near the *g* = *g*_e_ region and some resonances spreading up- and downfield. We note that the feature at 14.8 T in sample D2 at 50 K corresponds is a spike, (*i.e.*, an artifact) rather than a true resonance.

Finally, at 5 K, all samples are in the magnetically ordered regime (the sample temperature lying well below the experimental Weiss temperatures), meaning that the electron spin microstates are poorly defined, as spin clusters develop and microstates of individual paramagnetic centers become mixed by the exchange interactions within the cluster.^107,108^ This manifests as a spreading of resonances to higher and lower field (Figure 12a,d). For pristine NMMO, five resonances are seen, with some additional features imposed on the high-field resonance, which we attribute to different local magnetic environments.

At 5 K for points C2 and D2, the axial symmetry of the *g*-tensor is retained, as expected. The broadening of the resonances can be attributed to the smearing of resonances by the exchange interaction, in addition to the greater distribution of local environments generated during cycling. At point C5, a low-intensity isotropic resonance is observed; additional “spikes” are anomalies in the detection process, as confirmed from phasing (spikes in the out-of-phase component also exist but remain unaffected by phasing; these spikes are also absent when changing temperature). The loss of intensity on cooling may be ascribed to an increase in the energy gap between spin microstates due to strong exchange coupling in the system, resulting in an antiferromagnetic ground state with few transitions that can be excited using these frequencies, and a spreading of resonances over a wider field range.^111^

We note that all the EPR spectra may contain contributions from radicals in the electrolyte resulting from electrolyte degradation reactions, These contributions will be small in these samples since all samples were washed and dried prior to measuring. In the *operando* measurements, these signals if present are likely obscured by the carbon/sodium metal sharp signals. Mn-containing surface species, with different environments from those in the bulk will also be present, however, they are likely obscured by the EPR signals of the bulk.

### *Ex Situ* XAS and XANES

To investigate the redox processes taking place in NMMO over the first charge–discharge cycle further, *ex situ* X-ray absorption spectroscopy (XAS) measurements at the Mn and O *K*-edges were carried out (Figures S25 and S26). A brief outline of the results is given here; the reader is direct to the SI (Section 10) for deeper analysis.

In Stage 1, the Mn *K*-edge XANES shows a small increase in the edge energy, consistent with oxidation of Mn^3+^ to Mn^4+^, and an increase in the pre-edge intensity, suggesting greater distortions to the local Mn coordination environments. The O *K*-edge shows little change in the position or intensity of the edge and pre-edge features.

Over stage 2, the Mn *K*-edge XANES shows a decrease in the pre-edge energy and further increase in intensity, as well as a loss of features near the edge. Both indicate greater *p*–*d* mixing and an increase in octahedral distortions. In the O *K*-edge XAS, the features corresponding to transitions to the 1*s* to Mn(3*d, t*_2g_*)–O(2*p*) and 1*s* to Mn(3*d, e*_g_*)–O(2*p*) states increase in energy, a new pre-edge feature is observed between the two. The former indicates greater mixing of the Mn(3*d*) and O(2*p*) states, while the latter suggests the development of a new electronic state. This new feature could correspond to a transition to delocalized π-like states; the precise identity of this new state is challenging to pin down, however.^112^

During stages 3 and 4, the Mn *K*-edge undergoes little change, while the pre-edge becomes less intense and moves to higher energies, consistent with the decrease in local Mn distortions. The O *K*-edge pre-edge decreases in intensity and energy and resembles the spectrum for pristine NMMO.

## Discussion

### Structural Changes from SXRD and NMR

Building upon our previous work^42^ on NMMO, the SXRD and ^25^Mg NMR results presented here provide further evidence for Mg^2+^ migration at high states of charge. A quantitative assessment of the proportion of Mg^2+^ that migrates is challenging using both methods, owing to the width and low intensity of the observed reflections/resonances.

Migration of Mg^2+^ will drastically alter the local electronic structure around both O and Mn, as some O centers become “underbonded”; we anticipate that this manifests as an increase in Mn–O bond covalency. These changes in electronic structure may be teased apart by careful examination of the NMR and EPR data.

### Examining the Electronic Structure on Charge

Our earlier work^42^ showed that, on charging NMMO through stage 1, single-phase Na^+^ extraction from the pristine P2 phase occurs, with residual Mn^3+^ ions oxidizing to Mn^4+^. This was borne out in *ex situ* magnetometry results, where a decrease in the effective magnetic moment and more ferromagnetic Weiss constant was seen. The increase in sharpness of the ^17^O NMR resonances from C0 to C2 is also consistent with the loss of Mn^3+^ (which induces short *T*_2_s and severely broadened signals). This was also corroborated by *operando* X-band and *ex situ* high-frequency EPR, where a decrease in *g* and a smaller line width—a consequence of both longer spin–spin lifetimes (longer *T*_2e_s) and a smaller distribution in local environments—was seen. Additional evidence from Mn *K*-edge XANES and O *K*-edge XAS results also indicated oxidation of Mn (Figures S22 and S23), as well as slightly less mixing of the Mn 3*d* and O 2*p* states, which can be attributed to the loss of the JT-shortened Mn^3+^–O bonds.

During stage 2, μ_eff_ remained approximately constant, while θ gradually became strongly antiferromagnetic. The trend seen in μ_eff_—*i.e.*, a relatively flat profile throughout the charge plateau, with only a small increase at the very end of charge—is consistent with a gradual oxidation of both Mn and O, such that the moment at Mn decreases while that at O increases. This is also consistent with the approximately constant *operando* EPR peak area (proportional to the magnetic susceptibility) over stage 2. On oxidizing both species, an increasingly strong antiferromagnetic interaction between the Mn and O^–^ centers or between the Mn and O_2_ is generated, causing the observed gradual decrease in θ (*i.e.*, a more antiferromagnetic net exchange interaction).

At the end of charge (C5), the effective magnetic moment per mole of NMMO is 3.21 μ_B_ mol^–1^, where the composition is Na_0.10_[Mg_0.28_Mn_0.72_]O_2_; note that this value differs from that in Figure 10 where it was presented as the moment per mole of Mn. Assuming a mixture of the material at the start of the charge plateau (nominally Na_0.56_[Mg_0.28_Mn_0.72_]O_2_, whose moment is 3.30 μ_B_ per mole, comprising 18% of the material at the end of charge, based on Na^+^ content) and a Na^+^-deficient O2-phase at the end of charge (Na_0_[Mg_0.28_Mn_0.72_]O_2_, 82% of the material at the end of charge), then the total moment of NMMO at C5 is 2.89 μ_B_ per mole for model B (Mn^4+^ and O^–^, with O^–^ spins antiparallel to Mn spins) and 3.42 μ_B_ per mole for D (trapped O_2_ with additional O^1.5–^ and O^*n*–^, 1.78 < *n* < 2). We note that degradation involving electrolyte-surface reactions and potentially particle cracking, leading to increased surface area, could lead to the formation of paramagnetic surface species, or differences in local environments at and near the surface. However, no clear signature of isolated and additional paramagnetic environments was observed.

Additional evidence of the charge compensation mechanism during stage 2 was obtained from the *operando* X-band and *ex situ* high-frequency EPR data. Here, the approximately constant *g*-factor (closer to *g*_e_ than the pristine material) combined with the drop in signal intensity and increased line width (constant peak area) indicated overall weaker exchange interactions in NMMO and little change of the magnetic susceptibility of NMMO over stage 2. The absence of any additional resonances at the end of charge—which would correspond to trapped O_2_ or electrons localized to O—suggests that no new species are generated or that these species cannot be detected by EPR (owing to a large zero-field splitting, as for Mn^3+^), or that delocalized states form. Since molecular O_2_ has been observed in both layered systems and disordered rocksalts at low temperatures (<50 K) using EPR previously,^113,114^ this leads to one of two conclusions: either (1) O_2_ formation is unlikely, or (2) any O_2_ which forms is effectively part of a delocalized state, thus contributing to the broad EPR resonance observed (in this case, any description of a molecular O_2_ or (O_2_)^*n–*^like state seems inappropriate).

Previous reports of the EPR spectrum of O_2_ at X-band and at room temperature show many lines, corresponding to the vibrational and rotational modes of the O_2_ molecule.^115^ No such features are seen in our *operando* EPR, suggesting the absence of O_2_. One possibility is that resonances from O_2_ are severely broadened due to, for example, restricted motion, the resonances then becoming indistinguishable from the baseline; such signals might become visible at high frequencies under variable temperatures: this is not observed. More generally, when O_2_ is cooled below the condensation point (typically <10 K), an EPR signal whose breadth depends on the surrounding matrix is observed (*e.g.*, in silica gels, a broad line, approximately 100 mT peak-to-peak, is seen, while sharp features, around 2.5 mT, are seen when in N_2_). At high frequencies (>95 GHz), transitions between microstates separated by the zero field splitting may be readily observed.^116^ In our system, the presence of other paramagnetic centers nearby any trapped O_2_ in the NMMO lattice may lead to broadening of the EPR signal so that it is no longer observable.^115,117^

DOS calculations of the O_2_ trapped state, suggest that the formation of O_2_ results in strongly localized states, so it seems unlikely that “molecular” O_2_ forms in this system. This is further supported by XANES and XAS measurements, which suggest the formation of a new mixed Mn(3*d*)–O(2*p*) state, corresponding to the 529.8 eV shoulder seen in O *K*-edge XAS at the end of charge. The appearance of the new peak could be a transition to a new hybridized state (whose nature is unknown), or it could be a splitting of the Mn(3*d*, *t*_2g_*)–O(2*p*) state by the exchange interaction. If the latter, the splitting corresponds to approximately 0.8 eV; given that the exchange splitting of the O *K-*pre-edge peak can be empirically related to the number of unpaired electrons in the system (splitting = 0.6 eV × number of spins in the paramagnetic state),^118^ this suggests a total of 1.33 unpaired spins, which may be generated by strong antiferromagnetic coupling between the three unpaired electrons from Mn^4+^ with the unpaired electrons from nearby O^*n*–^ centers. These strong interactions are also predicted in *ab initio* calculations of the Mn^4+^–O^–^ exchange coupling constant, estimated to be approximately −200 K (or 17 meV; see SI for further information). Based on the greater EPR line width, however, it would appear that the exchange interactions in NMMO weaken along the charge plateau. We suggest this weakening is not a bulk property, as *ex situ* magnetometry suggests the net exchange interaction strength increases, but in fact indicates the observable spins experience weaker exchange interactions, consistent with the reduced peak-to-peak height of the *operando* EPR signal observed during stage 2. These observable spins are likely not part of the Mn^4+^–O^*n*–^ units, so the X-band *operando* EPR spectra do not provide information on these Mn^4+^–O^*n*–^ units.

It seems unlikely that the new peak in the O *K*-edge XAS stems from O_2_, as the pre-edge for molecular O_2_ lies just below 531 eV and is strongly peaked. The effect of binding to Mn on the XAS spectrum (in terms of transition energies and intensities) is, however, unknown. The Mn *K*-edge XANES is also consistent with delocalized redox: the small change in the Mn *K*-pre-edge peaks suggests that both the Mn and O are involved in redox along the charge plateau, but it is expected the majority of the redox occurs on O, as the relative change in energy of the pre-edge is larger for O than Mn. It should also be noted that the absolute energy of charge compensation state D (trapped O_2_) is the second lowest of all the configurations. We suggest, however, that the energy barrier to forming this state is extremely large, as it requires a rearrangement of the TM sublattice. If Mn migrates, there will be a large energy penalty due to the *d*^3^ (Mn^4+^) center passing through a tetrahedral transition state.^119,120^ Such a migration would therefore involve an energy barrier that far exceeds the overpotentials applied and is therefore unlikely to account for the bulk of this high voltage capacity. Note that some Mn migration may take place in certain unique local environments, however, further *ab initio* calculations are required to determine under what conditions Mn migration becomes favorable.^89,90^ Indeed, it is possible, however, that Mn could migrate as Mn^5+^ or Mn^2+^, though we observe no evidence for such a migration from NMR, EPR or XANES. We anticipate that these migrations are likely irreversible, as the driving force for migration must be substantial to overcome the large energy barrier, making the barrier to return to the initial configuration very high. This would further contribute to the path hysteresis between charge and discharge in NMMO.

While several mechanisms may coexist, our results suggest that the formation of strongly magnetically coupled and/or delocalized-like states are more consistent with the observed data. Indeed, the tendency of Mg and Mn in NMMO to honeycomb order^43^ will encourage π-redox behavior.^40^ We acknowledge, however, that other mechanisms may be present where the structure deviates from honeycomb order. For example, for several Mg^2+^ nearest neighbors, one might expect sufficient free volume to enable O_2_ trapping.

### Evolution of Electronic Structure on Discharge

During stage 3, the effective magnetic moment of NMMO remains approximately constant; this is analogous to stage 2, where concomitant oxidation of Mn and O lead to a relatively flat profile for *μ*_eff_. In stage 3, reduction of these delocalized Mn–O states results in an increase in *μ*_eff_ at Mn but a decrease at O. At the same time, *θ* increases, suggesting a less antiferromagnetic interaction between Mn and O, as there is less unpaired electron density on O to interact with Mn. This gradual change in electronic structure is also reflected in the EPR: the *g*-factor of NMMO remains approximately constant throughout discharge, with a much broader distribution in the *g*-values than seen on charge (seen in both the line width and peak area in *operando* EPR and the *g*-strain in high-frequency EPR, respectively).

Discharging through stage 3 results in the migration of Mg^2+^ back to the octahedral sites in the transition metal layers, as seen the ^25^Mg NMR spectrum. It is anticipated that not all the Mg^2+^ will return to these sites, owing to the asymmetric energy profile seen;^42^ quantification of this using ^25^Mg NMR will likely be challenging. Nevertheless, based on the observed integrals of the 8000 to 14 000 ppm region of the spectrum (corresponding to Mg^2+^ in the TM layers), a semiquantitative estimate from ^25^Mg NMR suggests that 30% of Mg^2+^ in the sample remains trapped in the Na^+^ layers; SXRD suggests this value is 5%. It is expected that the true value lies between these, but more likely closer to the estimate from SXRD than NMR, as the SXRD refinement will be (marginally) more quantitative. The migration process back into the TM layers is likely driven by Na^+^ insertion into the O-type layers, causing the Mg^2+^ to be repelled back into the Mn layer. As seen in our earlier work,^42^ the energy barrier for the migration process is, however, much higher in the presence of Na^+^, further suggesting that some of the Mg^2+^ will remain kinetically trapped in the Na^+^ layer and prevent slipping back to a P-type layer.

The presence of residual Mg^2+^ in the Na^+^ layers will change both the interlayer and intralayer exchange coupling constants, as the local Mn–O–Mn bond angles and distances will change, causing a change in the Weiss constant (compared to the pristine material), as observed. Furthermore, the absence of some Mg^2+^ in the TM layer on subsequent charge cycles will likely change the degree of overlap between Mn and O orbitals, resulting in a new manifold of electronic states and thereby changing the voltage at which oxidation and reduction occurs. This is consistent with theoretical observations from the delocalized π redox charge compensation scheme.^40^

It is anticipated that the large voltage hysteresis seen between charge and discharge stems almost exclusively from the Mg^2+^ migration. A recent study of Na_2_Mn_3_O_7_ also suggested that O redox was stabilized by strong antiferromagnetic interactions in delocalized Mn–O states.^88^ This material exhibits no metal migration processes and has a limited voltage hysteresis, suggesting that, in NMMO, Mg^2+^ migration dominates the voltage hysteresis seen. It is, however, also possible that the delocalized Mn–O states drop in energy once oxidized (owing to the strong antiferromagnetic interactions between Mn and O) and are only repopulated at lower voltages on discharge; this would also contribute to the observed voltage hysteresis, as seen in previous studies.^17,18^

Stage 4 is dominated by Mn^4+^ reduction, as evidenced by the net stronger antiferromagnetic interactions (reflected in θ) and the increase in μ_eff_. This is accompanied by a decrease in the *operando* EPR peak area, as fewer spins are excited by EPR on forming more Mn^3+^. The *g*-factor of NMMO during stage 4 (as measured from *operando* EPR) remains approximately constant, suggesting that the local chemical environment of the unpaired electrons again undergoes little change. High-frequency EPR reveals a small decrease in *g*, but with a broad distribution. As mentioned earlier, the absolute *g* values cannot be (readily) compared, but the changes can; assuming the true *g* value lies close to 1.98 for pristine NMMO (consistent with *operando* X-band measurements and with reported literature values^101,106,110,121^), this small decrease in *g* is a move away from the free-electron *g*-value (*i.e.*, it is expected that the true *g* value is below 1.98). This suggests an increase in the extent of spin–orbit coupling, which we attribute to a decrease in the energy between the ground and excited states (consistent with the lower energy O *K*-pre-edge feature).

## Conclusions

In this work, the evolution of the electronic structure of Na_0.67_Mg_0.28_Mn_0.72_O_2_ (NMMO) and its charge compensation mechanism has been presented. Initially on charging, Mn^3+^ is oxidized to Mn^4+^; this is associated with the extraction of Na^+^ from the P2 phase. Further charging results in the development of the *Z*-phase and migration of Mg^2+^ from the octahedral sites in the TMO_2_ layers to the vacant tetrahedral sites in the Na^+^ layer. This migration process results in some of the O centers becoming undercoordinated, raising the energy of their electrons and generating a new set of electronic states. The nature of these states was identified by a combination of *ab initio* calculations, bulk magnetic susceptibility measurements, *operando* and high-frequency EPR, Mn and O *K*-edge XAS. Bulk magnetic susceptibility ruled out the presence of isolated holes on O aligned either ferro- or antiferromagnetically with Mn, while *operando* and high-frequency EPR, as well as Mn and O *K*-edge XAS ruled out the formation of trapped molecular O_2_, leaving the most likely mechanism to be the formation of delocalized states between Mn and the O centers nearby the migrated Mg^2+^, the spins on these Mn and O being antiferromagnetically aligned.

Overall, the results presented here suggest that the charge compensation mechanism in NMMO is primarily the oxidation of oxygen stabilized by strong antiferromagnetic interactions with Mn^4+^ species and delocalization of charge onto Mn. During charge, the highest occupied Mn–O states are depopulated, resulting in a gradual change in the electronic structure around both Mn and O, with the majority of the changes occurring around O, as these centers dominate these states. The formation of O^*n*–^–like (*n* < 2) species results in strong antiferromagnetic coupling with Mn and stabilization of these states. On discharge, the delocalized states are repopulated, with the voltage hysteresis almost exclusively arising from the asymmetric profile to Mg^2+^ migration found previously.^42^ Once repopulated, some of the Mn^4+^ is then reduced back to Mn^3+^, up to the limit of Na^+^ solubility in the lattice. The precise oxidation state and degree to which the antiferromagnetic Mn–O states are (de)localized could not be determined from these results, but an *ab initio* investigation of the formation of delocalized states in different local environments of Mn (*i.e.*, different numbers of Mn and Mg nearest neighbors) would prove invaluable to studying the true extent of (de)localization.

The results shown here indicate that the dominant bulk redox mechanism relies on the formation of these strongly antiferromagnetic states; we acknowledge that other mechanisms may be invoked in local environments that deviate from the average honeycomb ordered structure or at grain boundaries or pores within the material. This is perhaps why studies of different O redox materials have concluded different mechanisms.

The implication of these findings is that O redox appears to be strongly correlated with the local structures in materials. Each material will have a range of local structures, which themselves differ in type and distribution. While most of the O-redox-active materials studied have O-based states just below the Fermi level available for oxidation, the way in which the unpaired electrons generated on O are stabilized depends intimately on the local chemical structure. If the new states developed during charge are high in energy and encourage degradation—for example by partially reversible or irreversible phase transformations—the cathode should be redesigned to avoid such transformations, perhaps by removing the mobile redox-inactive dopants from the TM layer or by preventing their rearrangement and migration, for example, by “pillaring”.^122^ For example, Li migration is mitigated in Na layered materials because of the larger Na-oxygen layer.^123,124^

Future research on O redox materials should focus on determining the factors which govern mechanism heterogeneity and attempting to control this heterogeneity synthetically, for example by generating “perfect” superstructures, in which all local TM and O environments are fully determined and known and remain unchanged during cycling. As part of such studies, noninvasive experimental techniques such as NMR and EPR, combined with *ab initio* calculations, are invaluable in teasing apart these complex mechanisms.

## Supporting Information Available

A detailed experimental, additional computational results (density of states and NMR parameters), additional ^25^Mg and ^17^O NMR spectra, additional bulk magnetic susceptibility results, additional *operando* X-band and *ex situ* high-frequency EPR results, as well as X-ray absorption spectra (PDF)

## References

[ref1] Slater M. D.; Kim D.; Lee E.; Johnson C. S. Sodium-Ion Batteries. Adv. Funct. Mater. 2013, 23 (8), 947–958. 10.1002/adfm.201200691.

[ref2] Huang Y.; Wang Z.; Guan M.; Wu F.; Chen R. Toward Rapid-Charging Sodium-Ion Batteries Using Hybrid-Phase Molybdenum Sulfide Selenide-Based Anodes. Adv. Mater. 2020, 32, 2003534 10.1002/adma.202003534. 32844532

[ref3] Chayambuka K.; Mulder G.; Danilov D. L.; Notten P. H. L. Sodium-Ion Battery Materials and Electrochemical Properties Reviewed. Adv. Energy Mater. 2018, 8 (16), 1800079 10.1002/aenm.201800079.

[ref4] Yabuuchi N.; Kubota K.; Dahbi M.; Komaba S. Research Development on Sodium-Ion Batteries. Chem. Rev. 2014, 114 (23), 11636–11682. 10.1021/cr500192f. 25390643

[ref5] Delmas C.; Carlier D.; Guignard M. The Layered Oxides in Lithium and Sodium-Ion Batteries: A Solid-State Chemistry Approach. Adv. Energy Mater. 2021, 11, 2001201 10.1002/aenm.202001201.

[ref6] Manthiram A. A Reflection on Lithium-Ion Battery Cathode Chemistry. Nat. Commun. 2020, 11 (1), 1550 10.1038/s41467-020-15355-0. 32214093 PMC7096394

[ref7] Han M. H.; Gonzalo E.; Singh G.; Rojo T. A Comprehensive Review of Sodium Layered Oxides: Powerful Cathodes for Na-Ion Batteries. Energy Environ. Sci. 2015, 8 (1), 81–102. 10.1039/C4EE03192J.

[ref8] Radin M. D.; Hy S.; Sina M.; Fang C.; Liu H.; Vinckeviciute J.; Zhang M.; Whittingham M. S.; Meng Y. S.; Van Der Ven A. Narrowing the Gap between Theoretical and Practical Capacities in Li-Ion Layered Oxide Cathode Materials. Adv. Energy Mater. 2017, 7, 1602888 10.1002/aenm.201602888.

[ref9] Zhang M.; Kitchaev D. A.; Lebens-Higgins Z.; Vinckeviciute J.; Zuba M.; Reeves P. J.; Grey C. P.; Whittingham M. S.; Piper L. F. J.; Van der Ven A.; Meng Y. S. Pushing the Limit of 3d Transition Metal-Based Layered Oxides That Use Both Cation and Anion Redox for Energy Storage. Nat. Rev. Mater. 2022, 7, 522–540. 10.1038/s41578-022-00416-1.

[ref10] Assat G.; Tarascon J.-M. Fundamental Understanding and Practical Challenges of Anionic Redox Activity in Li-Ion Batteries. Nat. Energy 2018, 3 (5), 373–386. 10.1038/s41560-018-0097-0.

[ref11] Assat G.; Delacourt C.; Corte D. A. D.; Tarascon J.-M. Editors’ Choice—Practical Assessment of Anionic Redox in Li-Rich Layered Oxide Cathodes: A Mixed Blessing for High Energy Li-Ion Batteries. J. Electrochem. Soc. 2016, 163 (14), A2965–A2976. 10.1149/2.0531614jes.

[ref12] Zhao C.; Wang Q.; Lu Y.; Hu Y.-S.; Li B.; Chen L. Review on Anionic Redox for High-Capacity Lithium- and Sodium-Ion Batteries. J. Phys. Appl. Phys. 2017, 50 (18), 183001 10.1088/1361-6463/aa646d.

[ref13] Li C.; Geng F.; Hu B.; Hu B. Anionic Redox in Na-Based Layered Oxide Cathodes: A Review with Focus on Mechanism Studies. Mater. Today Energy 2020, 17. 10.1016/J.MTENER.2020.100474.

[ref14] Maitra U.; House R. A.; Somerville J. W.; Tapia-Ruiz N.; Lozano J. G.; Guerrini N.; Hao R.; Luo K.; Jin L.; Pérez-Osorio M. A.; Massel F.; Pickup D. M.; Ramos S.; Lu X.; McNally D. E.; Chadwick A. V.; Giustino F.; Schmitt T.; Duda L. C.; Roberts M. R.; Bruce P. G. Oxygen Redox Chemistry without Excess Alkali-Metal Ions in Na2/3[Mg0.28Mn0.72]O2. Nat. Chem. 2018, 10 (3), 288–295. 10.1038/nchem.2923. 29461536

[ref15] House R. A.; Rees G. J.; Pérez-Osorio M. A.; Marie J.-J.; Boivin E.; Robertson A. W.; Nag A.; Garcia-Fernandez M.; Zhou K.-J.; Bruce P. G. First-Cycle Voltage Hysteresis in Li-Rich 3d Cathodes Associated with Molecular O2 Trapped in the Bulk. Nat. Energy 2020, 5, 777–785. 10.1038/s41560-020-00697-2.

[ref16] Mortemard de Boisse B.; Nishimura S.; Watanabe E.; Lander L.; Tsuchimoto A.; Kikkawa J.; Kobayashi E.; Asakura D.; Okubo M.; Yamada A. Highly Reversible Oxygen-Redox Chemistry at 4.1 V in Na4/7–x[□1/7Mn6/7]O2 (□: Mn Vacancy). Adv. Energy Mater. 2018, 8 (20), 1800409 10.1002/aenm.201800409.

[ref17] Gent W. E.; Lim K.; Liang Y.; Li Q.; Barnes T.; Ahn S.-J.; Stone K. H.; McIntire M.; Hong J.; Song J. H.; Li Y.; Mehta A.; Ermon S.; Tyliszczak T.; Kilcoyne D.; Vine D.; Park J.-H.; Doo S.-K.; Toney M. F.; Yang W.; Prendergast D.; Chueh W. C. Coupling between Oxygen Redox and Cation Migration Explains Unusual Electrochemistry in Lithium-Rich Layered Oxides. Nat. Commun. 2017, 8 (1), 2091 10.1038/s41467-017-02041-x. 29233965 PMC5727078

[ref18] Hong J.; Gent W. E.; Xiao P.; Lim K.; Seo D.-H.; Wu J.; Csernica P. M.; Takacs C. J.; Nordlund D.; Sun C.-J.; Stone K. H.; Passarello D.; Yang W.; Prendergast D.; Ceder G.; Toney M. F.; Chueh W. C. Metal–Oxygen Decoordination Stabilizes Anion Redox in Li-Rich Oxides. Nat. Mater. 2019, 18 (3), 256–265. 10.1038/s41563-018-0276-1. 30718861

[ref19] Yabuuchi N.; Hara R.; Kubota K.; Paulsen J.; Kumakura S.; Komaba S. A New Electrode Material for Rechargeable Sodium Batteries: P2-Type Na2/3[Mg0.28Mn0.72]O2 with Anomalously High Reversible Capacity. J. Mater. Chem. A 2014, 2 (40), 16851–16855. 10.1039/C4TA04351K.

[ref20] Kim E. J.; Maughan P. A.; Bassey E. N.; Clément R. J.; Ma L. A.; Duda L. C.; Sehrawat D.; Younesi R.; Sharma N.; Grey C. P.; Armstrong A. R. Importance of Superstructure in Stabilizing Oxygen Redox in P3-Na0.67Li0.2Mn0.8O2. Adv. Energy Mater. 2022, 12 (3), 2102325 10.1002/aenm.202102325.

[ref21] Yabuuchi N. Solid-State Redox Reaction of Oxide Ions for Rechargeable Batteries. Chem. Lett. 2017, 46 (4), 412–422. 10.1246/cl.161044.

[ref22] Boivin E.; House R. A.; Pérez-Osorio M. A.; Marie J. J.; Maitra U.; Rees G. J.; Bruce P. G. Bulk O2 Formation and Mg Displacement Explain O-Redox in Na0.67Mn0.72Mg0.28O2. Joule 2021, 5 (5), 1267–1280. 10.1016/j.joule.2021.04.006.

[ref23] Kim E. J.; Ma L. A.; Duda L. C.; Pickup D. M.; Chadwick A. V.; Younesi R.; Irvine J. T. S.; Robert Armstrong A. Oxygen Redox Activity through a Reductive Coupling Mechanism in the P3-Type Nickel-Doped Sodium Manganese Oxide. ACS Appl. Energy Mater. 2020, 3 (1), 184–191. 10.1021/acsaem.9b02171.

[ref24] Sharma N.; Tapia-Ruiz N.; Singh G.; Armstrong A. R.; Pramudita J. C.; Brand H. E. A.; Billaud J.; Bruce P. G.; Rojo T. Rate Dependent Performance Related to Crystal Structure Evolution of Na 0.67 Mn 0.8 Mg 0.2 O 2 in a Sodium-Ion Battery. Chem. Mater. 2015, 27 (20), 6976–6986. 10.1021/acs.chemmater.5b02142.

[ref25] Assat G.; Foix D.; Delacourt C.; Iadecola A.; Dedryvère R.; Tarascon J.-M. Fundamental Interplay between Anionic/Cationic Redox Governing the Kinetics and Thermodynamics of Lithium-Rich Cathodes. Nat. Commun. 2017, 8 (1), 2219 10.1038/s41467-017-02291-9. 29263321 PMC5738393

[ref26] Xu J.; Sun M.; Qiao R.; Renfrew S. E.; Ma L.; Wu T.; Hwang S.; Nordlund D.; Su D.; Amine K.; Lu J.; McCloskey B. D.; Yang W.; Tong W. Elucidating Anionic Oxygen Activity in Lithium-Rich Layered Oxides. Nat. Commun. 2018, 9 (1), 947 10.1038/s41467-018-03403-9. 29507369 PMC5838240

[ref27] Reeves P. J.; Seymour I. D.; Griffith K. J.; Grey C. P. Characterizing the Structure and Phase Transition of Li 2 RuO 3 Using Variable-Temperature 17 O and 7 Li NMR Spectroscopy. Chem. Mater. 2019, 31 (8), 2814–2821. 10.1021/acs.chemmater.8b05178.

[ref28] Li X.; Li X.; Monluc L.; Chen B.; Tang M.; Chien P.-H.; Feng X.; Hung I.; Gan Z.; Urban A.; Hu Y.-Y.; Li X.; Monluc L.; Chen B.; Tang M.; Chien P.-H.; Feng X.; Hu Y.-Y.; Urban A.; Hung I.; Gan Z. Stacking-Fault Enhanced Oxygen Redox in Li2MnO3. Adv. Energy Mater. 2022, 12 (18), 2200427 10.1002/AENM.202200427.

[ref29] Mortemard de Boisse B.; Liu G.; Ma J.; Nishimura S.; Chung S.-C.; Kiuchi H.; Harada Y.; Kikkawa J.; Kobayashi Y.; Okubo M.; Yamada A. Intermediate Honeycomb Ordering to Trigger Oxygen Redox Chemistry in Layered Battery Electrode. Nat. Commun. 2016, 7, 11397 10.1038/ncomms11397. 27088834 PMC4837481

[ref30] Clément R. J.; Bruce P. G.; Grey C. P. Review—Manganese-Based P2-Type Transition Metal Oxides as Sodium-Ion Battery Cathode Materials. J. Electrochem. Soc. 2015, 162 (14), A2589–A2604. 10.1149/2.0201514jes.

[ref31] Seo D.-H.; Lee J.; Urban A.; Malik R.; Kang S.; Ceder G. The Structural and Chemical Origin of the Oxygen Redox Activity in Layered and Cation-Disordered Li-Excess Cathode Materials. Nat. Chem. 2016, 8 (7), 692–697. 10.1038/nchem.2524. 27325096

[ref32] Luo K.; Roberts M. R.; Hao R.; Guerrini N.; Pickup D. M.; Liu Y.-S.; Edström K.; Guo J.; Chadwick A. V.; Duda L. C.; Bruce P. G. Charge-Compensation in 3d-Transition-Metal-Oxide Intercalation Cathodes through the Generation of Localized Electron Holes on Oxygen. Nat. Chem. 2016, 8, 684–691. 10.1038/nchem.2471. 27325095

[ref33] Luo K.; Roberts M. R.; Guerrini N.; Tapia-Ruiz N.; Hao R.; Massel F.; Pickup D. M.; Ramos S.; Liu Y.-S.; Guo J.; Chadwick A. V.; Duda L. C.; Bruce P. G. Anion Redox Chemistry in the Cobalt Free 3d Transition Metal Oxide Intercalation Electrode Li[Li 0.2 Ni 0.2 Mn 0.6]O 2. J. Am. Chem. Soc. 2016, 138 (35), 11211–11218. 10.1021/jacs.6b05111. 27498756

[ref34] McCalla E.; Abakumov A. M.; Saubanère M.; Foix D.; Berg E. J.; Rousse G.; Doublet M.-L.; Gonbeau D.; Novák P.; Van Tendeloo G.; Dominko R.; Tarascon J.-M. Visualization of O-O Peroxo-like Dimers in High-Capacity Layered Oxides for Li-Ion Batteries. Science 2015, 350 (6267), 1516–1521. 10.1126/science.aac8260. 26680196

[ref35] Grimaud A.; Hong W. T.; Shao-Horn Y.; Tarascon J.-M. Anionic Redox Processes for Electrochemical Devices. Nat. Mater. 2016, 15 (2), 121–126. 10.1038/nmat4551. 26796721

[ref36] McCalla E.; Sougrati M. T.; Rousse G.; Berg E. J.; Abakumov A.; Recham N.; Ramesha K.; Sathiya M.; Dominko R.; Van Tendeloo G.; Novák P.; Tarascon J.-M. Understanding the Roles of Anionic Redox and Oxygen Release during Electrochemical Cycling of Lithium-Rich Layered Li 4 FeSbO 6. J. Am. Chem. Soc. 2015, 137 (14), 4804–4814. 10.1021/jacs.5b01424. 25811894

[ref37] House R. A.; Maitra U.; Pérez-osorio M. A.; Lozano J. G.; Jin L.; Somerville J. W.; Duda L. C.; Nag A.; Walters A.; Zhou K.; Roberts M. R.; Bruce P. G. Superstructure Control of First-Cycle Voltage Hysteresis in O-Redox Cathodes. Nature 2020, 577, 502–508. 10.1038/s41586-019-1854-3. 31816625

[ref38] House R. A.; Maitra U.; Jin L.; Lozano J. G.; Somerville J. W.; Rees N. H.; Naylor A. J.; Duda L. C.; Massel F.; Chadwick A. V.; Ramos S.; Pickup D. M.; McNally D. E.; Lu X.; Schmitt T.; Roberts M. R.; Bruce P. G. What Triggers Oxygen Loss in Oxygen Redox Cathode Materials?. Chem. Mater. 2019, 31 (9), 3293–3300. 10.1021/acs.chemmater.9b00227.

[ref39] Gent W. E.; Abate I. I.; Yang W.; Nazar L. F.; Chueh W. C. Design Rules for High-Valent Redox in Intercalation Electrodes. Joule 2020, 4 (7), 1369–1397. 10.1016/j.joule.2020.05.004.

[ref40] Kitchaev D. A.; Vinckeviciute J.; Van Der Ven A. Delocalized Metal–Oxygen π-Redox Is the Origin of Anomalous Nonhysteretic Capacity in Li-Ion and Na-Ion Cathode Materials. J. Am. Chem. Soc. 2021, 143 (4), 1908–1916. 10.1021/jacs.0c10704. 33481574

[ref41] Rahman M. M.; Lin F. Oxygen Redox Chemistry in Rechargeable Li-Ion and Na-Ion Batteries. Matter 2021, 4 (2), 490–527. 10.1016/j.matt.2020.12.004.

[ref42] Bassey E. N.; Reeves P. J.; Jones M. A.; Lee J.; Seymour I. D.; Cibin G.; Grey C. P. Structural Origins of Voltage Hysteresis in the Na-Ion Cathode P2–Na0.67[Mg0.28Mn0.72]O2: A Combined Spectroscopic and Density Functional Theory Study. Chem. Mater. 2021, 33 (13), 4890–4906. 10.1021/acs.chemmater.1c00248. 34276134 PMC8280737

[ref43] Bassey E. N.; Seymour I. D.; Bocarsly J. D.; Keen D. A.; Pintacuda G.; Grey C. P. Superstructure and Correlated Na+ Hopping in a Layered Mg-Substituted Sodium Manganate Battery Cathode Are Driven by Local Electroneutrality. Chem. Mater. 2023, 35 (24), 10564–10583. 10.1021/acs.chemmater.3c02180. 38162043 PMC10753809

[ref44] Conan Doyle A. A Study in Scarlet; Ward Lock & Co., 1887.

[ref45] Wasylishen R.; Ashbrook S.; Wimperis S.; Vega A. J. Quadrupolar Nuclei in Solids. In NMR of Quadrupolar Nuclei in Solid Materials; Wiley, 2012; pp 17–44.

[ref46] Massiot D.; Fayon F.; Capron M.; King I.; Le Calvé S.; Alonso B.; Durand J.-O.; Bujoli B.; Gan Z.; Hoatson G. Modelling One- and Two-Dimensional Solid-State NMR Spectra. Magn. Reson. Chem. 2002, 40 (1), 70–76. 10.1002/mrc.984.

[ref47] Hung I.; Zhou L.; Pourpoint F.; Grey C. P.; Gan Z. Isotropic High Field NMR Spectra of Li-Ion Battery Materials with Anisotropy > 1 MHz. J. Am. Chem. Soc. 2012, 134 (4), 1898–1901. 10.1021/ja209600m. 22235803

[ref48] Halat D. M.; Dunstan M. T.; Gaultois M. W.; Britto S.; Grey C. P. Study of Defect Chemistry in the System La2-xSrxNiO4+δ by 17O Solid-State NMR Spectroscopy and Ni K-Edge XANES. Chem. Mater. 2018, 30 (14), 4556–4570. 10.1021/acs.chemmater.8b00747.

[ref49] Tartoni N.; Thompson S. P.; Tang C. C.; Willis B. L.; Derbyshire G. E.; Wright A. G.; Jaye S. C.; Homer J. M.; Pizzey J. D.; Bell A. M. T. IUCr. High-Performance X-Ray Detectors for the New Powder Diffraction Beamline I11 at Diamond. J. Synchrotron Radiat. 2008, 15 (1), 43–49. 10.1107/S0909049507046250. 18097077

[ref50] Thompson S. P.; Parker J. E.; Potter J.; Hill T. P.; Birt A.; Cobb T. M.; Yuan F.; Tang C. C. Beamline I11 at Diamond: A New Instrument for High Resolution Powder Diffraction. Rev. Sci. Instrum. 2009, 80 (7), 075107 10.1063/1.3167217. 19655981

[ref51] Coelho A. A. *TOPAS* and *TOPAS-Academic*: An Optimization Program Integrating Computer Algebra and Crystallographic Objects Written in C++. J. Appl. Crystallogr. 2018, 51 (1), 210–218. 10.1107/S1600576718000183.

[ref52] Kresse G.; Hafner J. Ab Initio Molecular Dynamics for Liquid Metals. Phys. Rev. B 1993, 47 (1), 558–561. 10.1103/PhysRevB.47.558. 10004490

[ref53] Kresse G.; Hafner J. Ab Initio Molecular-Dynamics Simulation of the Liquid-Metalamorphous- Semiconductor Transition in Germanium. Phys. Rev. B 1994, 49 (20), 14251–14269. 10.1103/PhysRevB.49.14251. 10010505

[ref54] Kresse G.; Furthmüller J. Efficiency of Ab-Initio Total Energy Calculations for Metals and Semiconductors Using a Plane-Wave Basis Set. Comput. Mater. Sci. 1996, 6 (1), 15–50. 10.1016/0927-0256(96)00008-0.

[ref55] Dovesi R.; Erba A.; Orlando R.; Zicovich-Wilson C. M.; Civalleri B.; Maschio L.; Rérat M.; Casassa S.; Baima J.; Salustro S.; Kirtman B. Quantum-Mechanical Condensed Matter Simulations with CRYSTAL. Wiley Interdiscip. Rev.: Comput. Mol. Sci. 2018, 8 (4), e1360 10.1002/wcms.1360.

[ref56] Kresse G.; Joubert D. From Ultrasoft Pseudopotentials to the Projector Augmented-Wave Method. Phys. Rev. B 1999, 59 (3), 1758–1775. 10.1103/PhysRevB.59.1758.

[ref57] Blöchl P. E. Projector Augmented-Wave Method. Phys. Rev. B 1994, 50 (24), 17953–17979. 10.1103/PhysRevB.50.17953. 9976227

[ref58] Anisimov V. I.; Zaanen J.; Andersen O. K. Band Theory and Mott Insulators: Hubbard U Instead of Stoner I. Phys. Rev. B 1991, 44 (3), 943–954. 10.1103/PhysRevB.44.943. 9999600

[ref59] Anisimov V. I.; Solovyev I. V.; Korotin M. A.; Czyyk M. T.; Sawatzky G. A. Density-Functional Theory and NiO Photoemission Spectra. Phys. Rev. B 1993, 48 (23), 16929–16934. 10.1103/PhysRevB.48.16929. 10008291

[ref60] Liechtenstein A. I.; Anisimov V. I.; Zaanen J. Density-Functional Theory and Strong Interactions: Orbital Ordering in Mott-Hubbard Insulators. Phys. Rev. B 1995, 52 (8), R5467 10.1103/PhysRevB.52.R5467. 9981806

[ref61] Zhou F.; Cococcioni M.; Kang K.; Ceder G. The Li Intercalation Potential of LiMPO 4 and LiMSiO 4 Olivines with M = Fe, Mn, Co, Ni. Electrochem. Commun. 2004, 6, 1144–1148. 10.1016/j.elecom.2004.09.007.

[ref62] Toumar A. J.; Ong S. P.; Richards W. D.; Dacek S.; Ceder G. Vacancy Ordering in O3 -Type Layered Metal Oxide Sodium-Ion Battery Cathodes. Phys. Rev. Appl. 2015, 4 (6), 064002 10.1103/PhysRevApplied.4.064002.

[ref63] Monkhorst H. J.; Pack J. D. Special Points for Brillouin-Zone Integrations. Phys. Rev. B 1976, 13 (12), 5188–5192. 10.1103/PhysRevB.13.5188.

[ref64] Becke A. D. Density-functional Thermochemistry. III. The Role of Exact Exchange. J. Chem. Phys. 1993, 98 (7), 5648–5652. 10.1063/1.464913.

[ref65] Lee C.; Yang W.; Parr R. G. Development of the Colle-Salvetti Correlation-Energy Formula into a Functional of the Electron Density. Phys. Rev. B 1988, 37 (2), 785–789. 10.1103/PhysRevB.37.785. 9944570

[ref66] Kim J.; Middlemiss D. S.; Chernova N. A.; Zhu B. Y. X.; Masquelier C.; Grey C. P. Linking Local Environments and Hyperfine Shifts: A Combined Experimental and Theoretical 31 P and 7 Li Solid-State NMR Study of Paramagnetic Fe(III) Phosphates. J. Am. Chem. Soc. 2010, 132 (47), 16825–16840. 10.1021/ja102678r. 21053901

[ref67] Middlemiss D. S.; Ilott A. J.; Clément R. J.; Strobridge F. C.; Grey C. P. Density Functional Theory-Based Bond Pathway Decompositions of Hyperfine Shifts: Equipping Solid-State NMR to Characterize Atomic Environments in Paramagnetic Materials. Chem. Mater. 2013, 25 (9), 1723–1734. 10.1021/cm400201t.

[ref68] Clément R. J.; Pell A. J.; Middlemiss D. S.; Strobridge F. C.; Miller J. K.; Whittingham M. S.; Emsley L.; Grey C. P.; Pintacuda G. Spin-Transfer Pathways in Paramagnetic Lithium Transition-Metal Phosphates from Combined Broadband Isotropic Solid-State MAS NMR Spectroscopy and DFT Calculations. J. Am. Chem. Soc. 2012, 134 (41), 17178–17185. 10.1021/ja306876u. 23004936

[ref69] Catti M.; Sandrone G.; Dovesi R. Periodic Unrestricted Hartree–Fock Study of Corundumlike Ti2O3 and V2O3. Phys. Rev. B 1997, 55 (24), 16122–16131. 10.1103/PhysRevB.55.16122.

[ref70] Catti M.; Valerio G.; Dovesi R.; Causà M. Quantum-Mechanical Calculation of the Solid-State Equilibrium MgO+α-Al2O3 ⇄ MgAl2O4 (Spinel) versus Pressure. Phys. Rev. B 1994, 49 (20), 14179–14187. 10.1103/PhysRevB.49.14179. 10010497

[ref71] Catti M.; Sandrone G.; Valerio G.; Dovesi R. Electronic, Magnetic and Crystal Structure of Cr2O3 by Theoretical Methods. J. Phys. Chem. Solids 1996, 57 (11), 1735–1741. 10.1016/0022-3697(96)00034-0.

[ref72] Schäfer A.; Horn H.; Ahlrichs R. Fully Optimized Contracted Gaussian Basis Sets for Atoms Li to Kr. J. Chem. Phys. 1992, 97 (4), 2571–2577. 10.1063/1.463096.

[ref73] Kutzelnigg W.; Fleischer U.; Schindler M. The IGLO-Method: Ab-Initio Calculation and Interpretation of NMR Chemical Shifts and Magnetic Susceptibilities; Springer: Berlin, Heidelberg, 1990; pp 165–262 10.1007/978-3-642-75932-1_3.

[ref74] Lee J.; Seymour I. D.; Pell A. J.; Dutton S. E.; Grey C. P. A Systematic Study of ^25^Mg NMR in Paramagnetic Transition Metal Oxides: Applications to Mg-Ion Battery Materials. Phys. Chem. Chem. Phys. 2017, 19 (1), 613–625. 10.1039/C6CP06338A. 27918022

[ref75] Bain G. A.; Berry J. F. Diamagnetic Corrections and Pascal’s Constants. J. Chem. Educ. 2008, 85 (4), 532–536. 10.1021/ed085p532.

[ref76] Nguyen H.; Bassey E.N.; Foley E.E.; Kitchaev D.A.; Giovine R.; Clement R.J. Operando Spin Probes for the Study of Battery Processes. J. Mag. Res. 2024, 368, 107772 10.1016/j.jmr.2024.107772. 39305685

[ref77] Barra A. L.; Hassan A. K.; Janoschka A.; Schmidt C. L.; Schünemann V. Broad-Band Quasi-Optical HF-EPR Spectroscopy: Application to the Study of the Ferrous Iron Center from a Rubredoxin Mutant. Appl. Magn. Reson. 2006, 30 (3), 385–397. 10.1007/BF03166208.

[ref78] Stoll S.; Schweiger A. EasySpin, a Comprehensive Software Package for Spectral Simulation and Analysis in EPR. J. Magn. Reson. 2006, 178 (1), 42–55. 10.1016/j.jmr.2005.08.013. 16188474

[ref79] Newville M. IFEFFIT: Interactive XAFS Analysis and FEFF Fitting. J. Synchrotron Radiat. 2001, 8 (2), 325–327. 10.1107/S0909049500016964. 11512767

[ref80] Ravel B.; Newville M. ATHENA, ARTEMIS, HEPHAESTUS: Data Analysis for X-Ray Absorption Spectroscopy Using IFEFFIT. J. Synchrotron Radiat. 2005, 12 (4), 537–541. 10.1107/S0909049505012719. 15968136

[ref81] Solé V.; Papillon E.; Cotte M.; Walter P.; Susini J. A Multiplatform Code for the Analysis of Energy-Dispersive X-Ray Fluorescence Spectra. Spectrochim. Acta Part B At. Spectrosc. 2007, 62 (1), 63–68. 10.1016/j.sab.2006.12.002.

[ref82] Yao Z.; Kim S.; He J.; Hegde V. I.; Wolverton C. Interplay of Cation and Anion Redox in Li _4_ Mn _2_ O _5_ Cathode Material and Prediction of Improved Li _4_ (Mn,M) _2_ O _5_ Electrodes for Li-Ion Batteries. Sci. Adv. 2018, 4 (5), eaao6754 10.1126/sciadv.aao6754. 29795779 PMC5959302

[ref83] Rozier P.; Sathiya M.; Paulraj A.-R.; Foix D.; Desaunay T.; Taberna P.-L.; Simon P.; Tarascon J.-M. Anionic Redox Chemistry in Na-Rich Na2Ru1–ySnyO3 Positive Electrode Material for Na-Ion Batteries. Electrochem. Commun. 2015, 53, 29–32. 10.1016/j.elecom.2015.02.001.

[ref84] Assat G.; Iadecola A.; Delacourt C.; Dedryvère R.; Tarascon J.-M. Decoupling Cationic–Anionic Redox Processes in a Model Li-Rich Cathode via Operando X-Ray Absorption Spectroscopy. Chem. Mater. 2017, 29 (22), 9714–9724. 10.1021/acs.chemmater.7b03434.

[ref85] Saubanère M.; McCalla E.; Tarascon J.-M.; Doublet M.-L. The Intriguing Question of Anionic Redox in High-Energy Density Cathodes for Li-Ion Batteries. Energy Environ. Sci. 2016, 9 (3), 984–991. 10.1039/C5EE03048J.

[ref86] House R. A.; Marie J. J.; Park J.; Rees G. J.; Agrestini S.; Nag A.; Garcia-Fernandez M.; Zhou K. J.; Bruce P. G. Covalency Does Not Suppress O2 Formation in 4d and 5d Li-Rich O-Redox Cathodes. Nat. Commun. 2021, 12 (1), 2975 10.1038/s41467-021-23154-4. 34016979 PMC8137948

[ref87] Sathiya M.; Abakumov A. M.; Foix D.; Rousse G.; Ramesha K.; Saubanère M.; Doublet M. L.; Vezin H.; Laisa C. P.; Prakash A. S.; Gonbeau D.; VanTendeloo G.; Tarascon J.-M. Origin of Voltage Decay in High-Capacity Layered Oxide Electrodes. Nat. Mater. 2015, 14 (2), 230–238. 10.1038/nmat4137. 25437258

[ref88] Zhao C.; Liu H.; Geng F.; Hu B.; Li C. Stable Electronic Structure Related with Mn4+O–• Coupling Determines the Anomalous Nonhysteretic Behavior in Na2Mn3O7. Energy Storage Mater. 2022, 48, 290–296. 10.1016/j.ensm.2022.02.049.

[ref89] Radin M. D.; Vinckeviciute J.; Seshadri R.; Van der Ven A. Manganese Oxidation as the Origin of the Anomalous Capacity of Mn-Containing Li-Excess Cathode Materials. Nat. Energy 2019, 4, 639–646. 10.1038/s41560-019-0439-6.

[ref90] Vinckeviciute J.; Kitchaev D. A.; Van der Ven A. A Two-Step Oxidation Mechanism Controlled by Mn Migration Explains the First-Cycle Activation Behavior of Li2MnO3-Based Li-Excess Materials. Chem. Mater. 2021, 33 (5), 1625–1636. 10.1021/ACS.CHEMMATER.0C03734.

[ref91] Kentgens A. P. M. A Practical Guide to Solid-State NMR of Half-Integer Quadrupolar Nuclei with Some Applications to Disordered Systems. Geoderma 1997, 80 (3–4), 271–306. 10.1016/S0016-7061(97)00056-6.

[ref92] Bassey E. N.; Reeves P. J.; Seymour I. D.; Grey C. P. 17O NMR Spectroscopy in Lithium-Ion Battery Cathode Materials: Challenges and Interpretation. J. Am. Chem. Soc. 2022, 144 (41), 18714–18729. 10.1021/jacs.2c02927. 36201656 PMC9585580

[ref93] Seymour I. D.; Middlemiss D. S.; Halat D. M.; Trease N. M.; Pell A. J.; Grey C. P. Characterizing Oxygen Local Environments in Paramagnetic Battery Materials via 17O NMR and DFT Calculations. J. Am. Chem. Soc. 2016, 138 (30), 9405–9408. 10.1021/jacs.6b05747. 27404908

[ref94] Gerothanassis I. P. Oxygen-17 NMR Spectroscopy: Basic Principles and Applications (Part I). Prog. Nucl. Magn. Reson. Spectrosc. 2010, 56 (2), 95–197. 10.1016/j.pnmrs.2009.09.002. 20633350

[ref95] Gerothanassis I. P. Oxygen-17 NMR Spectroscopy: Basic Principles and Applications (Part II). Prog. Nucl. Magn. Reson. Spectrosc. 2010, 57 (1), 1–110. 10.1016/j.pnmrs.2009.12.001. 20633360

[ref96] Schramm S.; Oldfield E. High-Resolution Oxygen-17 NMR of Solids. J. Am. Chem. Soc. 1984, 106 (9), 2502–2506. 10.1021/ja00321a002.

[ref97] Grey C. P.; Dupré N. NMR Studies of Cathode Materials for Lithium-Ion Rechargeable Batteries. Chem. Rev. 2004, 104 (10), 4493–4512. 10.1021/cr020734p. 15669160

[ref98] House R. A.; Rees G. J.; McColl K.; Marie J.-J.; Garcia-Fernandez M.; Nag A.; Zhou K.-J.; Cassidy S.; Morgan B. J.; Saiful Islam M.; Bruce P. G. Delocalized Electron Holes on Oxygen in a Battery Cathode. Nat. Energy 2023, 8, 351–360. 10.1038/s41560-023-01211-0.

[ref99] Dundon J. M. 17O NMR in Liquid O2. J. Chem. Phys. 1982, 76 (5), 2171–2173. 10.1063/1.443233.

[ref100] Mukherjee P.; Paddison J. A. M.; Xu C.; Ruff Z.; Wildes A. R.; Keen D. A.; Smith R. I.; Grey C. P.; Dutton S. E. Sample Dependence of Magnetism in the Next-Generation Cathode Material LiNi_0.8_Mn_0.1_Co_0.1_O_2_. Inorg. Chem. 2021 60 ( (1), ), 263–271. 10.1021/acs.inorgchem.0c02899. 33320647

[ref101] Stoyanova R.; Gorova M.; Zhecheva E. EPR Monitoring of Mn4+ Distribution in Li4Mn5O12 Spinels. J. Phys. Chem. Solids 2000, 61 (4), 615–620. 10.1016/S0022-3697(99)00245-0.

[ref102] Masquelier C.; Tabuchi M.; Ado K.; Kanno R.; Kobayashi Y.; Maki Y.; Nakamura O.; Goodenough J. B. Chemical and Magnetic Characterization of Spinel Materials in the LiMn2O4–Li2Mn4O9–Li4Mn5O12System. J. Solid State Chem. 1996, 123 (2), 255–266. 10.1006/jssc.1996.0176.

[ref103] Endres P.; Fuchs B.; Kemmler-Sack S.; Brandt K.; Faust-Becker G.; Praas H.-W. Influence of Processing on the Li:Mn Ratio in Spinel Phases of the System Li1 + xMn2 – xO4 – δ. Solid State Ion. 1996, 89 (3), 221–231. 10.1016/0167-2738(96)00349-9.

[ref104] Pell A. J.; Pintacuda G.; Grey C. P. Paramagnetic NMR in Solution and the Solid State. Prog. Nucl. Magn. Reson. Spectrosc. 2019, 111 (May), 1–271. 10.1016/j.pnmrs.2018.05.001. 31146806

[ref105] Azamat D. V.; Dejneka A.; Lancok J.; et al. Electron Paramagnetic Resonance Studies of Manganese Centers in SrTiO 3: Non-Kramers Mn 3+ Ions and Spin-Spin Coupled Mn 4+ Dimers. J. Appl. Phys. 2012, 111, 104119 10.1063/1.4723653.

[ref106] Stoyanova R.; Carlier D.; Sendova-Vassileva M.; Yoncheva M.; Zhecheva E.; Nihtianova D.; Delmas C. Stabilization of Over-Stoichiometric Mn4+ in Layered Na2/3MnO2. J. Solid State Chem. 2010, 183 (6), 1372–1379. 10.1016/j.jssc.2010.04.024.

[ref107] Keffer F.; Kittel C. Theory of Antiferromagnetic Resonance. Phys. Rev. 1952, 85 (2), 329–337. 10.1103/PhysRev.85.329.

[ref108] Kittel C. Theory of Antiferromagnetic Resonance. Phys. Rev. 1951, 82 (4), 565 10.1103/PhysRev.82.565.

[ref109] Barra A. L.; Brunel L. C.; Robert J. B. EPR Spectroscopy at Very High Field. Chem. Phys. Lett. 1990, 165 (1), 107–109. 10.1016/0009-2614(90)87019-N.

[ref110] Stoyanova R.; Ivanova S.; Zhecheva E.; Samoson A.; Simova S.; Tzvetkova P.; Barra A.-L. Correlations between Lithium Local Structure and Electrochemistry of Layered LiCo_1–2x_Ni_x_Mn_x_O_2_ Oxides: ^7^Li MAS NMR and EPR Studies. Phys. Chem. Chem. Phys. 2014, 16 (6), 2499–2507. 10.1039/C3CP54438A. 24356075

[ref111] Abragam A.; Bleaney B. Electron Paramagnetic Resonance of Transition Ions; Oxford University Press, 1970.

[ref112] Yang W.; Devereaux T. P. Anionic and Cationic Redox and Interfaces in Batteries: Advances from Soft X-Ray Absorption Spectroscopy to Resonant Inelastic Scattering. J. Power Sources 2018, 389, 188–197. 10.1016/j.jpowsour.2018.04.018.

[ref113] Zhao C.; Li C.; Liu H.; Qiu Q.; Geng F.; Shen M.; Tong W.; Li J.; Hu B. Coexistence of (O2)N– and Trapped Molecular O2 as the Oxidized Species in P2-Type Sodium 3d Layered Oxide and Stable Interface Enabled by Highly Fluorinated Electrolyte. J. Am. Chem. Soc. 2021, 143 (44), 18652–18664. 10.1021/jacs.1c08614. 34699720

[ref114] Liu H.; Li C.; Zhao C.; Tong W.; Hu B. Coincident Formation of Trapped Molecular O2 in Oxygen-Redox-Active Archetypical Li 3*d* Oxide Cathodes Unveiled by EPR Spectroscopy. Energy Storage Mater. 2022, 50, 55–62. 10.1016/j.ensm.2022.05.011.

[ref115] Eaton S. S.; Eaton G. R. EPR Spectra and Electron Spin Relaxation of O2. Appl. Magn. Reson. 2021, 52 (10), 1223–1236. 10.1007/s00723-021-01353-y. PMC11951249 40160976

[ref116] Pardi L. A.; Krzystek J.; Telser J.; Brunel L.-C. Multifrequency EPR Spectra of Molecular Oxygen in Solid Air. J. Magn. Reson. 2000, 146 (2), 375–378. 10.1006/jmre.2000.2175. 11001854

[ref117] Kon H. Paramagnetic Resonance of Molecular Oxygen in Condensed Phases. J. Am. Chem. Soc. 1973, 95 (4), 1045–1049. 10.1021/ja00785a009.

[ref118] Frati F.; Hunault M. O. J. Y.; De Groot F. M. F. Oxygen K-Edge X-Ray Absorption Spectra. Chem. Rev. 2020, 120 (9), 4056–4110. 10.1021/acs.chemrev.9b00439. 32275144 PMC7227067

[ref119] Seymour I. D.; Chakraborty S.; Middlemiss D. S.; Wales D. J.; Grey C. P. Mapping Structural Changes in Electrode Materials: Application of the Hybrid Eigenvector-Following Density Functional Theory (DFT) Method to Layered Li 0.5 MnO 2. Chem. Mater. 2015, 27 (16), 5550–5561. 10.1021/acs.chemmater.5b01674.

[ref120] Seymour I. D.; Wales D. J.; Grey C. P. Preventing Structural Rearrangements on Battery Cycling: A First-Principles Investigation of the Effect of Dopants on the Migration Barriers in Layered Li 0.5 MnO2. J. Phys. Chem. C 2016, 120 (35), 19521–19530. 10.1021/acs.jpcc.6b05307.

[ref121] Kalapsazova M.; Ivanova S.; Kukeva R.; Simova S.; Wegner S.; Zhecheva E.; Stoyanova R. Combined Use of EPR and 23Na MAS NMR Spectroscopy for Assessing the Properties of the Mixed Cobalt–Nickel–Manganese Layers of P3-NayCo1–2xNixMnxO2. Phys. Chem. Chem. Phys. 2017, 19 (39), 27065–27073. 10.1039/C7CP04849A. 28959996

[ref122] Wang Y.; Feng Z.; Cui P.; Zhu W.; Gong Y.; Girard M. A.; Lajoie G.; Trottier J.; Zhang Q.; Gu L.; Wang Y.; Zuo W.; Yang Y.; Goodenough J. B.; Zaghib K. Pillar-Beam Structures Prevent Layered Cathode Materials from Destructive Phase Transitions. Nat. Commun. 2021, 12 (1), 13 10.1038/s41467-020-20169-1. 33397895 PMC7782780

[ref123] Xu J.; Lee D. H.; Clément R. J.; Yu X.; Leskes M.; Pell A. J.; Pintacuda G.; Yang X.-Q.; Grey C. P.; Meng Y. S. Identifying the Critical Role of Li Substitution in P2–Nax [Liy Niz Mn1-y-z]O 2 (0 < x, y, *z* < 1) Intercalation Cathode Materials for High-Energy Na-Ion Batteries. Chem. Mater. 2014, 26 (2), 1260–1269. 10.1021/cm403855t.

[ref124] Clément R. J.; Xu J.; Middlemiss D. S.; Alvarado J.; Ma C.; Meng Y. S.; Grey C. P. Direct Evidence for High Na + Mobility and High Voltage Structural Processes in P2-Na x [Li y Ni z Mn 1–y–z]O2 (x, y, *z* ≤ 1) Cathodes from Solid-State NMR and DFT Calculations. J. Mater. Chem. A 2017, 5 (8), 4129–4143. 10.1039/C6TA09601H.

