# Supplementary material for: Strong Magnetic Exchange Interactions and Delocalized Mn–O States Enable High-Voltage Capacity in the Na-Ion Cathode P2–Na0.67[Mg0.28Mn0.72]O2
Source: Chem Mater. 2024 Sep 23;36(19):9493–515. doi: 10.1021/acs.chemmater.4c01320 (PMC11467838; doi:10.1021/acs.chemmater.4c01320)
Supplement: Supplementary file 1 — cm4c01320_si_001.pdf [file cm4c01320_si_001.pdf]

Supporting Information

Strong Magnetic Exchange Interactions and Delocalised Mn−O States Enable High-Voltage Capacity in the Na-Ion Cathode P2–$Na_{0.67}[Mg_{0.28}Mn_{0.72}]O_2$

Euan N. Bassey,[1,a] Howie Nguyen,[2] Teresa Insinna,[1] Jeongjae Lee,[3] Anne-Laure Barra,[4,5] Giannantonio Cibin,[6] Peter Bencok,[6] Raphaële J. Clément,[2] Clare P. Grey[1,*]

[1]Yusuf Hamied Department of Chemistry, University of Cambridge, Lensfield Road, Cambridge, CB2 1EW, United Kingdom

[2]Materials Department and Materials Research Laboratory, University of California, Santa Barbara, California 93106-5050, United States

[3]School of Earth and Environmental Sciences, Seoul National University, Seoul 08826, Korea

[4]Laboratoire National des Champs Magnétiques Intenses, CNRS, 38042 Grenoble Cedex 9, France

[5]Université Grenoble Alpes, 621 Av. Centrale, 38400 Saint-Martin-d'Hères, France

[6]Diamond Light Source, Harwell Science and Innovation Campus, Didcot, United Kingdom

[a]Present address: Materials Research Laboratory, University of California, Santa Barbara, Santa Barbara, CA 93106-5121, United States of America

* To whom correspondence should be addressed:

E-mail: cpg27@cam.ac.uk

Contents

1. **Experimental**
2. **Additional Computational Results**
3. **Additional ^{25}Mg NMR Results**
4. **Additional ^{17}O NMR Results**
5. **Solution-State NMR Results**
6. **Additional Bulk Magnetic Susceptibility Results**
7. **Additional *Operando* EPR Electrochemical Results**
8. ***Operando* EPR Background**
9. **Additional High-Frequency EPR Results**
10. **X-ray Absorption Spectroscopy Results: Mn *K*-edge XANES and O *K*-edge XAS**

List of Figures

Figure S1: DOS calculations for pristine NMMO using **(a)** the CRYSTAL code with Hyb35 functional, **(b)** the CRYSTAL code with Hyb20 functional and **(c)** the VASP code with DFT+*U*. The spin-up bands are shown as positive and the spin-down as negative. 16

Figure S2: DOS calculations for antiferromagnetic arrangements of O2-NMMO under **(a)** scheme A, **(b)** scheme B, **(c)** scheme C and **(d)** scheme D. DOSs are plotted with spin up bands as positive and spin down bands as negative. Note that these DOSs are calculated using the Hyb20 functional. 17
Figure S3: Ab initio density of states calculations for each of the O2-NMMO charge compensation models: (a) shows the pristine DOS, whilst (b) to (f) show the DOSs for schemes A to E, respectively. DOSs were calculated using a hybrid DFT functional with 20% Hartree-Fock. The upward- and downward-facing DOSs correspond to the spin up and down bands. 18
Figure S4: Magnetic cluster expansion fits for NMMO under charge compensation states **(a)** B and **(b)** D. 20
Figure S5: Comparison of the energies of states B and D using Hybrid 20 and 35 (from CRYSTAL calculations), as well as PBE+*U* and r^2SCAN (implemented in VASP). On the right, an zoom-in on the low-energy region is shown. Zero on these energy scales corresponds to B computed with the Hyb35 functional. 20
Figure S6: Ex situ ^{25}Mg NMR spectra of NMMO at different states of charge, with the probe background (empty stator and rotor). 22
Figure S7: VOCS ^{25}Mg NMR spectrum of pristine NMMO (C0), recorded at 16.4 T, static (T = 300 K). The lowest receiver offset was −2500 ppm and the highest 20000 ppm, in steps of 1250 ppm. 22
Figure S8: Comparison of ^{25}Mg NMR spectrum of NMMO at the end of first charge (C5), with simulated spectra corresponding to each of the five possible charge compensation states, A to E. 23
Figure S9: Fit to the ^{25}Mg NMR spectrum of pristine NMMO (C0) using a quadrupolar and chemical shift anisotropy model. Fitted values are highlighted to the right. Asterisks denote values which were frozen during the fit. 23
Figure S10: Isotropic slice of the pjMATPASS experiments for *ex situ* samples C0 and C2 of NMMO, acquired at 11.7 T under 60 kHz MAS rate. 24
Figure S11: Fits to the ^{17}O NMR spectra acquired under 60 kHz MAS and at **(a)** 11.7 T and **(b)** 16.4 T. 25
Figure S12: Fits to the ^{17}O NMR spectra acquired under 60 kHz MAS and at 11.7 with **(a)** four sites, **(b)** three sites and **(c)** two sites. 27
Figure S13: Comparison of the *ex situ* ^{17}O NMR spectrum acquired at point C5 of ^{17}O-enriched NMMO (at 11.7 T and 60 kHz MAS) with simulated spectra for each of the charge compensation mechanisms considered in this work. Arrows which face one side or the other in spectrum D indicate isotropic resonances beyond the plot window shown here. 28
Figure S14: Solution-state ^{19}F NMR spectra for the electrolyte used in NMMO half cells. Pristine corresponds to fresh electrolyte (which had been soaked in a coin cell but not been electrochemically cycled); “charged” corresponds to point C5, while discharged is point D3. The oscillatory features between approximately −120 ppm and −200 ppm correspond to background from the probe. 29
Figure S15: Solution-state ^{1}H NMR spectra for the electrolyte used in NMMO half cells. Pristine corresponds to fresh electrolyte (which had been soaked in a coin cell but not been electrochemically cycled); “charged” corresponds to point C5, while discharged is point D3. 30
Figure S16: Solution-state ^{17}O NMR spectra for the electrolyte used in NMMO half cells; the electrolyte was harvested from the same state of charge (end of discharge, D4). The “enriched” sample corresponds to electrolyte extracted from a cell where the cathode was ^{17}O-enriched (but the electrolyte was not), while the “unenriched” sample corresponds to electrolyte from an unenriched cathode (this electrolyte was also not enriched). 31

Figure S17: M(H) curves for ex situ samples of NMMO between 2 and 300 K over a field range −0.5 T to +0.5 T: **(a)** C0, **(b)** C1, **(c)** C2, **(d)** C3, **(e)** C4 and **(f)** C5. 33
Figure S18: M(H) curves for ex situ samples of NMMO between 2 and 300 K over a field range −0.5 T to +0.5 T: **(a)** D1, **(b)** D2, **(c)** D3 and **(d)** D4. 34
Figure S19: AC Magnetic Susceptibility data for pristine NMMO. **(a)** and **(c)** show the real (χ') and imaginary (χ'') components of the AC susceptibility for pristine NMMO, respectively, whilst **(b)** and **(d)** show the real and imaginary components of the AC susceptibility for NMMO at the end of charge, respectively. 35
Figure S20: Schematics of the exchange interactions in NMMO. Note that all interactions in NMMO are 90°-like and occur between Mn3+ and Mn3+, Mn3+ and Mn4+ or Mn4+ and Mn4+. The interactions are labelled as Ferromagnetic, F, or Antiferromagnetic, AF. Dashed lines indicate spin density which is transferred to a centre, whilst solid lines indicate spins which are already present and have been polarised. 36
Figure S21: Voltage profiles for an NMMO vs Na metal half cell in two different electrolytes: 1 M $NaPF_6$ in a 1:1:1 (by volume) mixture of ethylene carbonate (EC), dimethyl carbonate (DMC) and diethyl carbonate (DEC) and 1 M $NaPF_6$ in propylene carbonate (PC). 37
Figure S22: Background EPR spectrum of the *operando* EPR cell, recorded under the same conditions as the *operando* run. Note that the cathode and ruby were removed, but all other components were present. 38
Figure S23: Modification of the EasySpin fitted spectra field axis. In **(a)**, a screenshot of a fitted spectrum from EasySpin; **(b)** shows the same spectrum exported directly and plotted against the observed spectrum. In **(c)**, the data index is plotted against the field value to show its non-linearity (black) and the fit to a fifth-order polynomial function (red); translating the EasySpin field using this curve yields the true fit in **(d)**. 39
Figure S24: Ex situ continuous wave high-frequency (383.04 GHz) EPR on ^{17}O-enriched NMMO cathodes recorded at **(a)** 150 K, **(b)** 50 K and **(c)** 5 K. 41
Figure S25: *Ex situ* Mn *K*-edge XANES data for NMMO. **(a)** shows the region around the edge, with the regions which change most—at the pre-edge, edge and post-edge—highlighted in grey. For clarity, the pre-edge region is shown in **(b)**, with the fitted pre-edge energies shown in **(c)**. Fits were achieved using two Pseudo-Voigt peaks and a smooth step function background. A trendline has been added as a guide to the eye. 42
Figure S26: Ex situ O *K*-edge XAS total fluorescence yield (TFY) data for NMMO. **(a)** shows the spectra collected at each state of charge, whilst **(b)** shows the pre-edge region of the spectrum and **(c)** shows the fitted energies of the pre-edge peaks (fit using pseudo-Voigt peaks and a quadratic background). 44

List of Tables

Table S1: Open circuit voltages (OCVs) of cells cycled to a given cutoff voltage, after at least one hour's rest; standard errors are shown in parentheses. 6
Table S2: Compositions of each of the five compensation states, A to E, examined in this work, before ("initial") and after ("final") relaxation. Note that, for paramagnetic species, the spin orientations are denoted in superscript as ↑ for up and ↓ for down. The total spin of the cell, *S*, is also given. 10
Table S3: Calculated ^{25}Mg NMR parameters for pristine NMMO and NMMO under each of the charge compensation states, A to E, using two levels of hybrid functionals, one with 35% Hartree-Fock exchange (Hyb35) and one with 20% (Hyb20). Calculations are performed assuming a field strength of 16.4 T and an experimental temperature of 300 K. Quantities listed include: the isotropic hyperfine shift, $\delta_{hyp,iso}$, the dipolar hyperfine shift anisotropy,

$\Delta\delta_{hyp}$, the asymmetry of the dipolar hyperfine tensor, η_{hyp}, the quadrupolar coupling constant, C_Q, the second-order quadrupole-induced shift, δ_{QIS}, and the asymmetry of the electric field gradient tensor, η_Q. 11
Table S4: Calculated overlap populations for different Mg/Mn–O bonds found in NMMO in the pristine state and under each charge compensation mechanism; standard errors are shown in parentheses. Overlap populations were determined from density of states calculations using two levels of hybrid functionals: one with 35% Hartree-Fock (Hyb35) and one with 20% (Hyb20). 12
Table S5: Calculated ^{17}O Quadrupolar NMR parameters for NMMO under each of the charge compensation states, A to E, using two levels of hybrid functionals, one with 35% Hartree-Fock exchange (Hyb35) and one with 20% (Hyb20). Calculations are performed assuming a field strength of 11.7 T and an experimental temperature of 318 K (to account for the frictional heating incurred under 60 kHz magic angle spinning). Quantities listed include: the second-order quadrupole-induced shift, δ_{QIS}, the quadrupolar coupling constant, C_Q, and the asymmetry of the electric field gradient tensor, η_Q. 13
Table S6: Calculated ^{17}O Hyperfine NMR parameters for NMMO under each of the charge compensation states, A to E, using two levels of hybrid functionals, one with 35% Hartree-Fock exchange (Hyb35) and one with 20% (Hyb20). Calculations are performed assuming a field strength of 11.7 T and an experimental temperature of 318 K (to account for the frictional heating incurred under 60 kHz magic angle spinning). Quantities listed include: the isotropic hyperfine shift, $\delta_{hyp,iso}$, the dipolar hyperfine shift anisotropy, $\Delta\delta_{hyp}$, and the asymmetry of the dipolar hyperfine tensor, η_{hyp}. 14
Table S7: Calculated and observed ^{17}O isotropic hyperfine parameters, A_{iso}, for O_2* and •OH. [a] from ref. 44 and [b] from ref. 45 15
Table S8: Nearest-neighbour magnetic exchange constants, J, obtained from the magnetic cluster expansions of charge compensation states B and D. Note that $J_{Mn\text{-}O}$ represents the exchange constant between Mn^{4+} and a nearby O^{n-} ($n \approx 1$ for B, $1.0 < n < 2.0$ for D), while $J_{Mn\text{-}O2}$ is the exchange constant between Mn^{4+} and O_2 molecules. 21
Table S9: Fitted ^{17}O hyperfine and quadrupolar parameters from the fits at 11.7 T and 16.4 T. Note that all sites except the O–$(Mn^{3+})_x$ site were fit using combined chemical shift anisotropy and quadrupolar models; the O–$(Mn^{3+})_x$ site was fit to a Gaussian/Lorentzian peakshape. Values listed include: the isotropic hyperfine shift, δ_{iso}, the dipolar hyperfine shift anisotropy, $\Delta\delta_{hyp}$, the asymmetry of the dipolar hyperfine tensor, η_{hyp}, the quadrupolar coupling constant, C_Q, and the asymmetry of the electric field gradient tensor, η_Q. 26

1. Experimental

Synthesis. A detailed synthetic route and sample preparation scheme is detailed in refs. [1,2]. $Na_{0.67}[Mg_{0.28}Mn_{0.72}]O_2$ was synthesised *via* a high-temperature solid-state reaction as described previously. Stoichiometric quantities of Na_2CO_3, MgO and Mn_2O_3 were ball milled together (400 rpm, two hours total), pressed into a pellet and heated to 1073 K (10 K min^{-1}) for 10 hours under flowing O_2 (< 30 mL min^{-1}), followed by a natural cooling process. The pellet was ground in an Ar-filled glovebox (H_2O and O_2 < 1 ppm), reheated to 973 K (10 K min^{-1}) and then immediately quenched to room temperature. For ^{25}Mg enrichment, the same synthetic procedure was carried out, using ^{25}MgO as the starting reagent. For ^{17}O enrichment, approximately 150 mg of as-synthesised $Na_{0.67}[Mg_{0.28}Mn_{0.72}]O_2$ was taken, packed into an alumina crucible and then loaded into a quartz tube which was subsequently evacuated and then refilled with $^{17}O_2$ gas (Nukem, 70 at.% enrichment) to a pressure of approximately 1.1 bar. The tube was then sealed and reheated to 973 K for 24 hours, before being quenched to room temperature and immediately transferred to an Ar-filled glovebox.

Cathodes of $Na_{0.67}[Mg_{0.28}Mn_{0.72}]O_2$ were prepared as per the method in refs. [1,2]. For all experiments, a 1.0 M $NaPF_6$ (Acros Organics, 98.5+%; dried at 393 K for 12 hours under dynamic vacuum) in propylene carbonate (PC; Solvionic, < 50 ppm H_2O) electrolyte was used unless otherwise stated. All cells in this work were half-cells, using a Na metal disc as the anode; these discs were punched out from Na metal (Sigma-Aldrich, 99.0%, 13 mm diameter). All electrochemical tests were carried out using NMMO/Na metal half-cells. Each cell was assembled from a stack of one cathode, one glass fibre separator (Whatman, GF/B, 0.68 mm thick, 16 mm diameter, 1.0 µm pore size) soaked with 150 µL electrolyte and one Na metal disc.

Electrochemical measurements on these half cells were performed using either a BioLogic MPG2 potentiostat/galvanostat instrument running EC-Lab software (^{17}O and ^{25}Mg NMR and high-frequency EPR experiments), or an Arbin potentiostat/galvanostat (Mn *K*-edge XANES, O *K*-edge XAS and bulk magnetic susceptibility measurements). The half cells were galvanostatically charged at a rate of 10 mA g^{-1} (corresponding to approximately *C*/19, for a theoretical *C* rate determined from the time elapsed and current applied, assuming that x in $Na_x[Mg_{0.28}Mn_{0.72}]O_2$ runs between 0 and 1 and that no parasitic reactions take place during cycling). This slow cycling rate was chosen to minimise effects of concentration gradients in the active material and to avoid the high overpotentials often seen at high voltages.

***Ex situ* Sample Preparation.** Cathodes of NMMO were cycled up to a given cutoff voltage and then allowed to relax for at least one hour (see Table S1 for a list of cutoff voltages and open circuit voltages), before being extracted from the cell inside an Ar-filled glovebox and either scraped off the Al foil backing (for NMR, EPR, SXRD and bulk magnetic susceptibility measurements) or peeled off the Al foil intact (for XANES and XAS measurements).

Table S1: Open circuit voltages (OCVs) of cells cycled to a given cutoff voltage, after at least one hour's rest; standard errors are shown in parentheses.

Sample label	Cutoff	OCV (V)
C0	-	-
C1	2.90 V (1st Charge)	2.855(2)
C2	4.22 V (1st Charge)	3.830(2)
C3	4.247 V (1st Charge)	4.067(2)
C4	4.265 V (1st Charge)	4.118(2)
C5	4.50 V (1st Charge)	3.983(2)
D1	3.50 V (1st Discharge)	3.774(2)
D2	2.75 V (1st Discharge)	2.972(2)
D3	2.142 V (1st Discharge)	2.264(2)
D4	1.50 V (1st Discharge)	1.867(2)

Solid-state ^{25}Mg Nuclear Magnetic Resonance Spectroscopy. ^{25}Mg-enriched *ex situ* cycled cathodes and pristine powders were packed into 4 mm diameter ZrO_2 magic angle spinning (MAS) rotors in an Ar-filled glovebox, with inert PTFE tape packed at either end to ensure the sample was centred in the rotor. No rotor spent longer than 5 minutes outside of the glovebox before being inserted into the magnet under a protective atmosphere of flushing nitrogen gas. ^{25}Mg NMR spectra were referenced to solid MgO at 26.0 ppm. NMR spectra were acquired on a Bruker Avance III (16.4 T) using a Bruker 4 mm MAS probe with two 33 pF capacitors installed on the *X* channel; spectra were recorded static, with an effective $\frac{\pi}{2}$ pulse length of 2.21 μs (which corresponds to $\frac{\pi}{6}$, to account for the strong quadrupolar interaction of ^{25}Mg and ensure all ^{25}Mg centres are in the quadrupolar liquid limit[3]). Hahn-echo pulse sequences (90°–τ–180°–τ–acquire) at different receiver frequency offsets were used to record individual slices in a variable-offset cumulative spectrum (VOCS). The recycle delay (10 ms) was set such that the bulk, paramagnetically shifted signal was recorded quantitatively. A background spectrum (acquired *via* VOCS using the same number of scans, recycle delay and receiver offset frequencies) was also obtained and subtracted from the observed spectra.

Solid-state ^{17}O Nuclear Magnetic Resonance Spectroscopy. ^{17}O-enriched pristine powder and *ex situ* cycled cathodes were packed into 1.3 mm diameter ZrO_2 MAS rotors in an Ar-filled glovebox; no rotor spent longer than 5 minutes outside of the glovebox before being inserted into the magnet under a protective atmosphere of flushing nitrogen gas. ^{17}O NMR spectra were referenced to liquid $H_2{}^{17}O$ (0 ppm). NMR spectra were acquired on a Bruker Avance III (11.7 T) using a Bruker 1.3 mm MAS probe. A MAS frequency of 60 kHz was used, with an effective $\frac{\pi}{2}$ pulse length of 0.66 μs (which corresponds to $\frac{\pi}{6}$, due to the quadrupolar nature of ^{17}O[3]). A rotor-synchronised Hahn-echo pulse sequence (90°–τ–180°–τ–acquire) was used. Spectra were scaled according to the mass of the sample and number of residuals recorded. The recycle delay (5 ms; at least $5T_1$) was set such that the bulk, paramagnetically shifted signal was recorded quantitatively, while the diamagnetic signal due to electrolyte decomposition products was suppressed. Projection magic angle turning phase-adjusted sideband separation (pjMATPASS) experiments were also recorded to separate the isotropic resonances from the overlapping spinning sideband manifold.[4–6]

Solution-state ^{1}H, ^{17}O and ^{19}F Nuclear Magnetic Resonance Spectroscopy. Na:NMMO half cells containing unenriched or ^{17}O-enriched NMMO were cycled until end of charge (C*x*) and end of discharge (D*x*). Upon disassembly of the cells in an Ar-filled glovebox, the glass fiber separator was soaked in 0.8 mL of dry d_8-THF (Eurisotop, 99.5% D) for 15 min to extract

electrolyte components. The solution was then transferred in an airtight NMR tube equipped with a J-Young's tap. The pristine electrolyte (1 M $NaPF_6$ in PC) was also measured, with the original electrolyte volume (150 µL) being added to a glass fiber separator and then extracted as described above. One dimensional ^{1}H, ^{19}F{^{1}H}, ^{31}P{^{1}H} and ^{17}O spectra were collected for all samples on a Bruker AVANCE III HD 9.4 T (ω_H = 400 MHz) spectrometer using a BBFO probe. The spectra were internally referenced to d_8-THF at 1.8 ppm (δ ^{1}H) and at 19 ppm (δ ^{17}O), and internally referenced to $NaPF_6$ at -74.5 ppm (δ ^{19}F).

Synchrotron X-Ray Diffraction. *Ex situ* diffraction patterns of both the pristine material and cycled cathodes were recorded at beamline I11 at the Diamond Light Source,[7,8] with a wavelength of 0.82652 Å over a range 2θ = 0 to 150°. Samples were loaded into borosilicate glass capillaries (outer diameter 0.5 mm) inside an Ar-filled glovebox and sealed using two-component epoxy resin. Rietveld refinements of the diffraction patterns were carried out using the TOPAS Academic 6.0 software package.[9]

***Ab initio* density of states calculations and chemical shift calculations.** To simplify calculations, a model system, O2-$Na_0[Mg_{1/3}Mn_{2/3}]O_2$, was constructed, using the lattice parameters and atomic coordinates obtained from *ex situ* synchrotron X-ray diffraction at the end of charge. For most calculations, all Mg^{2+} centres were placed in the tetrahedral sites in the vacant Na^+ layers. Throughout the calculations, (2 × 2 × 1) supercells were constructed to account for both inter- and intralayer exchange interactions between the paramagnetic centres. The initial charge distributions are detailed in the results section.

The density of states was calculated for a series of charge compensation schemes, all based on the $Na_0[Mg_{1/3}Mn_{2/3}]O_2$ model system described in the results, using both the VASP[10,11] and CRYSTAL codes.[12,13] In VASP, the projector-augmented wave method (PAW)[14,15] was employed, with a spin-polarised Perdew-Burke-Ernzerhof exchange correlation functional and Hubbard *U* model[16,17] applied within the rotationally invariant formalism proposed by Liechstenstein *et al.*,[18] to correct for the known deficiencies of pure functionals for highly localised 3*d* states.[19] The plane-wave energy cutoff was set to 520 eV, and an effective Hubbard *U* parameter for Mn, $U_{eff} = U - J$ = 3.9 eV, where *U* and *J* are the effective on-site Coulomb and exchange parameters (J = 1 eV), respectively, was chosen, in line with previous work on the parent material, Na_xMnO_2.[20] SCF cycles were converged with an energy tolerance of 10^{-8} eV and the Brillouin zone was samples with a Monkhorst-Pack[21] Gamma-centred *k*-mesh of density < 0.025 Å^{-1}.

Periodic spin-polarised DFT calculations of the density of states and hyperfine and quadrupole-induced shifts were performed in CRYSTAL. Hyperfine parameters were calculated with B3LYP[22] and a modified B3LYP hybrid functional containing 20% and 35% Hartree-Fock exchange, referred to as Hyb20 and Hyb35, respectively. These weights were chosen based on the success of these functionals in calculating the properties of *TM* compounds and have been previously reported to provide an upper and lower bound on experimental shifts.[23,24]

The calculations employed two basis sets: a smaller basis set for geometry optimisations (denoted BS-I) and a more extended set for the single-point hyperfine calculations (BS-II). Geometry optimisations were carried out on a ferromagnetic configuration, with an energy tolerance of 10^{-5} AU, a root-mean-square force gradient tolerance of 3 × 10^{-4} AU and integral thresholds of 10^{-8}, 10^{-8}, 10^{-8}, 10^{-8} and 10^{-16} for the Coulomb overlap, Coulomb penetration, exchange overlap and *g* and *n* series exchange penetration, respectively. The BS-I sets were taken—without modification—from solid-state studies by Catti *et al.*,[25–27] whilst the BS-II sets comprised bases from the Ahlrichs set for metal ions[28] and IGLO-III basis set for O.[29] Single-point calculations to obtain the density of states were performed using integral thresholds of

10^{-7}, 10^{-7}, 10^{-7}, 10^{-7} and 10^{-17} for the Coulomb overlap, Coulomb penetration, exchange overlap and *g* and *n* series exchange penetration, respectively, whilst the energy tolerance was set to 10^{-8} AU. The BS-I sets are of the form (15*s*7*p*)/[1*s*3*sp*] for Na, (15*s*3*p*)/[1*s*3*sp*] for Mg, (20*s*12*p*5*d*)/[1*s*4*sp*2*d*] for Mn and (14*s*6*p*)/[1*s*3*sp*] for O. The BS-II sets are of the form (11*s*7*p*)/[7*s*3*p*] for Na, (11*s*7*p*)/[6*s*3*p*] for Mg, (13*s*10*p*5*d*)/[7*s*6*p*3*d*] for Mn and (10*s*6*p*2*d*)/[6*s*5*p*2*d*] for O. In both cases, the values in parentheses denote the number of Gaussian primitives and the values in square brackets denote the contraction scheme.

The magnetic cluster expansion of NMMO in charge compensation states B (antiferromagnetic Mn^{4+} and O^{-}like states) and D (trapped O_2) were carried out; unit cells of each system were decorated with at least 50 unique magnetic configurations and the energy computed using VASP under the r^2SCAN functional,[30–32] with the same energy cutoffs and convergence limits as above. The relative energies of each state under r^2SCAN and PBE+*U* were comparable. A simple Ising magnetic Hamiltonian was fit to the calculated data using a Lasso model, as implemented in the Scikit-learn module for python, using a *α*-value of 1 μeV/Mn. Enumeration of the magnetic configurations and determination of the magnetic correlations (S_iS_j) were performed using the CASM software package.[33]

Bulk magnetic susceptibility measurements. The bulk magnetic susceptibility measurements were carried out on powder samples and *ex situ* cycled cathodes (approximately 3 – 15 mg) using a Quantum Design Magnetic Property Measurement System 3 (MPMS) superconducting quantum interference device (SQUID) magnetometer. The zero-field cooled (ZFC) and field-cooled (FC) susceptibilities were measured in a field of 0.1 T over a temperature range 2–300 K. As *M*(*H*) is linear in this field range, the small-field approximation to the susceptibility, $\chi \simeq \frac{M}{H}$, was assumed to be valid. The data for each sample were corrected for diamagnetism of the sample using Pascal's constants.[34]

***Operando* Electron Paramagnetic Resonance Spectroscopy.** *Operando* EPR measurements were carried out on an in-house quartz cell containing the following stack: an Al foil current collector (5 mm diameter), a cathode disc of NMMO (5 mm diameter), a glass fibre separator (Whatman, GF/B, 0.68 mm thick, 5 mm diameter, 1.0 μm pore size) soaked in 8 μL of electrolyte (1.0 M $NaPF_6$ in a 1:1:1 volumetric mixture of ethylene carbonate, diethylene carbonate and dimethyl carbonate), a Na metal foil disc (5 mm diameter) and a Cu foil current collector. The cell was cycled at a rate of 10 mA g^{-1} between 1.5 V and 4.5 V *vs* $Na^{0/+}$. Spectra were recorded continuously during cycling, with 420 seconds between each spectral slice; these slices comprised four individual spectra, to improve signal-to-noise. Spectra were fitted in the following way: initially, the ruby signals were fitted to Lorentzian lineshapes using the EasySpin toolbox in MATLAB,[35] from these fits the integral and *g*-factor of the broad resonance and ruby resonances were obtained. Then, the central region of the spectrum (216 to 531 mT) containing the broad cathode resonance and the sharp carbon/Na resonance were fit using a Lorentzian lineshape for the broad feature and a combined Gaussian/Lorentzian for the sharp feature.

High-frequency Electron Paramagnetic Resonance Spectroscopy. The high-frequency EPR (HF-EPR) spectra of pristine powders and *ex situ* cycled cathodes were recorded on a double-pass transmission EPR spectrometer built at the high magnetic field laboratory in Grenoble, France.[36] The frequencies were varied from 255 to 383 GHz using a 127 GHz

frequency source and its multipliers (Virginia Diodes), whilst detection was achieved using a bolometer (QMC instruments). Temperatures were recorded using a variable-temperature insert (Cryogenic) at 5 K, 50 K and 150 K. The EPR spectra were fitted to a powder pattern lineshape with axial or isotropic *g* tensors using the EasySpin toolbox for MATLAB.[35] The EPR spectra were fitted to a powder pattern lineshape with axial or isotropic *g* tensors using the EasySpin toolbox for MATLAB and then modified to account for the non-linearity of the field and assumed linearity of the data by EasySpin (see section 8).

Mn *K*-edge X-ray Absorption Near Edge Spectroscopy. *Ex situ* XANES was performed at beamline B18 at the Diamond Light Source. Mn *K*-edge data were recorded at ambient temperature in transmission mode above and below the absorption edge (6539 eV). Samples were loaded into an in-house (Diamond) transfer chamber with transparent polyimide (Kapton) film windows. Three spectral scans were recorded for each sample; no changes between the first and last measurements were observed. Alignment, background removal and fitting of the XANES data was carried out in the Athena software package.[37,38]

O *K*-edge X-ray Absorption Spectroscopy. *Ex situ* XAS was performed at beamline I10 at the Diamond Light Source. O *K*-edge data were recorded at ambient temperature in both fluorescence yield (FY) and total electron yield (TEY) modes above and below the absorption edge of 543 eV. Samples were loaded onto an in-house (Diamond) transfer module using a conductive epoxy resin. Four spectral scans were recorded for each sample; no drift in the beam energy was observed between the first and last measurement. Alignment, background removal and fitting of the data was carried out in the PyMCA software package.[39]

2. Additional Computational Results

Our calculations employ hybrid functionals (as implemented in the CRYSTAL code), which have been shown to better capture the local spin and charged densities, and thus more accurately predict the hyperfine and quadrupolar NMR parameters,[12,23] a consequence of the localised Gaussian-type-orbital basis set used. We also note the success of these basis sets in calculating the hyperfine parameters of both peroxides and radical O species (see Table S7). Dispersion interactions were accounted for using a DFT-D3 correction.[40,41] The DOS results (see later) were benchmarked against calculations of the electronic structures obtained from the VASP plane-wave code,[10,11,42] to ensure these DOSs are consistent with DFT+*U* calculations performed elsewhere in the literature [Figure S1]. The DFT+*U* method was avoided, however, as it remains unclear whether a single *U* parameter can be used for all Mn sites, regardless of the local environment, and whether an additional *U* parameter is required to describe the contribution of O to the electronic states.[43] Such an investigation would require extensive testing of several combinations of *U* parameters, which is beyond the scope of this work. The calculations in this work are at the limit of the level of theory being used; the results presented are therefore contextualised and compared with the experimental results to produce a holistic picture of the charge compensation mechanism.

Table S2: Compositions of each of the five compensation states, A to E, examined in this work, before ("initial") and after ("final") relaxation. Note that, for paramagnetic species, the spin orientations are denoted in superscript as ↑ for up and ↓ for down. The total spin of the cell, *S*, is also given.

State	Initial State Composition	Final State Composition	Total Spin, *S*	Notes
A	$Na_0[Mg_{6,tet.}Mn_{12}^{4+,\uparrow}]O_{12}^{-,\uparrow}O_{24}^{2-}$	$Na_0[Mg_{6,tet.}Mn_{12}^{4+,\uparrow}]O_{12}^{-,\uparrow}O_{24}^{2-}$	24	
B	$Na_0[Mg_{6,tet.}Mn_{12}^{5+,\uparrow}]O_{36}^{2-}$	$Na_0[Mg_{6,tet.}Mn_{12}^{4+,\uparrow}]O_{12}^{-,\downarrow}O_{24}^{2-}$	12	
C	$Na_0[Mg_{6,tet.}Mn_{12}^{4.5+,\uparrow}]O_{12}^{1.5-,\uparrow}O_{24}^{2-}$	$Na_0[Mg_{6,tet.}Mn_6^{4+,\uparrow}Mn_6^{3.75+,\uparrow}]O_6^{-,\downarrow}O_6^{0.75-,\uparrow}O_{24}^{2-}$	18	
D	$Na_0[Mg_{6,tet.}Mn_{12}^{4+,\uparrow}](O_2)_2^{0,\uparrow}O_8^{1.5-,\uparrow}O_{24}^{2-}$	$Na_0[Mg_{6,tet.}Mn_{12}^{4+,\uparrow}](O_2)_2^{0,\uparrow}O^{1.5-,\downarrow}O_{28}^{n-,\uparrow}$	22	$2 < n < 1.78$
E	$Na_0[Mg_{6,oct.}Mn_{12}^{5+,\uparrow}]O_{36}^{2-}$	$Na_0[Mg_{6,oct.}Mn_{12}^{3.5+,\uparrow}]O_{36}^{1.5-,\downarrow}$	12	

Table S3: Calculated ^{25}Mg NMR parameters for pristine NMMO and NMMO under each of the charge compensation states, A to E, using two levels of hybrid functionals, one with 35% Hartree-Fock exchange (Hyb35) and one with 20% (Hyb20). Calculations are performed assuming a field strength of 16.4 T and an experimental temperature of 300 K. Quantities listed include: the isotropic hyperfine shift, $\delta_{hyp,iso}$, the dipolar hyperfine shift anisotropy, $\Delta\delta_{hyp}$, the asymmetry of the dipolar hyperfine tensor, η_{hyp}, the quadrupolar coupling constant, C_Q, the second-order quadrupole-induced shift, δ_{QIS}, and the asymmetry of the electric field gradient tensor, η_Q.

	System	Pristine	Pristine	A	B	C	C	D	D	D	E
	Number of Mn^{4+} n.n.	6	5	8	8	8	8	6	10	8	6 ($Mn^{3.5+}$)
	Number of Mn^{3+} n.n.	0	1	-	-	-	-	-	-	-	-
	Number of O^{n-} n.n.	-	-	4 (n = 1)	4 (n = 1)	4 (n = 0.75)	4 (n = 1)	-	-	-	6 (n = 1.5)
	Number of O_2 n.n.	-	-	-	0	0	0	1	0	0	0
Hyb35	$\delta_{hyp.,iso}$ (ppm)	10200	9310	920	3700	2940	1690	11310	5410	3020	20210
	$\Delta\delta_{hyp}$ (ppm)	1340	1427	-592	-395	-346	-643	1459	-786	-844	1801
	η_{hyp}	0.08	0.1	0.17	0.27	0.39	0.11	0.08	0.12	0.28	0.24
	C_Q (MHz)	8.33	7.34	5.8	6.02	6	5.83	6.27	6.97	4.59	6.37
	δ_{QIS} (ppm)	-361	-265	-144	-155	-154	-146	-193	-319	-100	-214
	η_Q	0.8	0.67	0.08	0.09	0.09	0.07	0.65	0.74	0.48	0.48
Hyb20	$\delta_{hyp.,iso}$ (ppm)	11700	10810	1110	4450	3660	1920	13360	6050	3620	22120
	$\Delta\delta_{hyp}$ (ppm)	1337	1398	-615	-395	-384	-628	1488	-777	-827	1790
	η_{hyp}	0.08	0.1	0.17	0.26	0.34	0.12	0.09	0.17	0.29	0.08
	C_Q (MHz)	8.05	7.3	5.6	5.84	5.84	5.61	6.38	6.96	4.66	5.12
	δ_{QIS} (ppm)	-332	-262	-135	-147	-146	-135	-197	-317	-101	-117
	η_Q	0.77	0.66	0.07	0.08	0.08	0.07	0.63	0.76	0.47	0.21

Overlap Populations

To understand how the relative covalency and ionicity of the *TM*–O bonds change under each of the charge compensation schemes, overlap populations were computed from the wavefunctions used to obtain the densities of state [Table S4]. In general, a higher overlap population indicates a more covalent bond, whilst a smaller overlap population suggests charge separation and a more ionic bond. As seen in the overlap populations below, migrating Mg^{2+} to the vacant tetrahedral sites in the Na^{+} O-type layers at the end of charge increases the covalency of the Mg–O bonds. Furthermore, the bonds between Mn^{m+} and O^{n-} ($3.75 < m < 4$; $0.9 < n < 1.8$) become less covalent under all the charge compensation schemes, as expected for an increase in the O orbital energies.

Table S4: Calculated overlap populations for different Mg/Mn–O bonds found in NMMO in the pristine state and under each charge compensation mechanism; standard errors are shown in parentheses. Overlap populations were determined from density of states calculations using two levels of hybrid functionals: one with 35% Hartree-Fock (Hyb35) and one with 20% (Hyb20).

State	Bond	Hyb35 Distance (Å)	Hyb35 Overlap Population (a.u.)	Hyb20 Distance (Å)	Hyb20 Overlap Population (a.u.)
Pristine	Mg^{2+}–O^{2-}	2.057(4)	0.099(5)	2.070(4)	0.099(5)
	Mn^{4+}–O^{2-}	1.923(13)	0.241(4)	1.937(13)	0.242(4)
A	Mg^{2+}–O^{2-}	1.926(11)	0.129(4)	1.926(11)	0.129(4)
	Mn^{4+}–O^{-}	1.912(2)	0.215(4)	1.912(2)	0.228(4)
	Mn^{4+}–O^{2-}	1.912(3)	0.253(3)	1.912(3)	0.249(3)
B	Mg^{2+}–O^{2-}	2.067(2)	0.102(2)	2.068(2)	0.099(2)
	Mn^{4+}–$O^{0.9-}$	2.001(7)	0.188(7)	1.930(7)	0.222(7)
	Mn^{4+}–O^{-}	1.973(8)	0.201(8)	1.929(8)	0.227(8)
	Mn^{4+}–O^{2-}	1.817(9)	0.266(8)	1.856(9)	0.254(9)
C	Mg^{2+}–O^{2-}	1.926(6)	0.130(5)	1.926(6)	0.131(5)
	$Mn^{3.75+}$–O^{-}_{down}	1.912(6)	0.214(2)	1.912(6)	0.225(2)
	$Mn^{3.75+}$–O^{2-}	1.912(6)	0.254(3)	1.912(6)	0.254(3)
	Mn^{4+}–O^{-}_{up}	1.912(6)	0.209(2)	1.912(6)	0.222(2)
	Mn^{4+}–O^{2-}	1.912(6)	0.252(3)	1.912(6)	0.249(3)
D	Mg^{2+}–O^{2-}	2.039(2)	0.101(3)	2.039(2)	0.102(2)
	Mg^{2+}–$O^{-}_{O2,\ far}$	3.118(18)	0.006(2)	3.118(18)	0.006(2)
	Mg^{2+}–$O^{-}_{O2,\ near}$	2.079(19)	0.100(2)	2.195(19)	0.089(2)
	Mn^{4+}–O^{-}_{O2}	1.956(19)	0.221(2)	1.956(19)	0.221(2)
	Mn^{4+}–$O^{1.5-}$	1.922(6)	0.236(6)	1.922(6)	0.243(6)
	Mn^{4+}–$O^{1.8-}$	1.876(3)	0.252(6)	1.876(3)	0.255(6)
	Mn^{4+}–O^{2-}	1.886(3)	0.249(6)	1.886(3)	0.251(6)
E	Mg^{2+}–$O^{1.5-}$	1.912(11)	0.112(3)	1.912(11)	0.113(3)
	$Mn^{3.5+}$–$O^{1.5-}$	1.912(8)	0.116(4)	1.912(8)	0.114(4)

Calculated ^{25}Mg and ^{17}O NMR Parameters

To assist interpretation of the observed ^{25}Mg and ^{17}O NMR spectra, NMR parameters of each of the charge compensation states (A to E) were calculated to obtain the simulated spectra shown in Figures 6, 7, 8 and 10 in the main text.

Table S5: Calculated ^{17}O Quadrupolar NMR parameters for NMMO under each of the charge compensation states, A to E, using two levels of hybrid functionals, one with 35% Hartree-Fock exchange (Hyb35) and one with 20% (Hyb20). Calculations are performed assuming a field strength of 11.7 T and an experimental temperature of 318 K (to account for the frictional heating incurred under 60 kHz magic angle spinning). Quantities listed include: the second-order quadrupole-induced shift, δ_{QIS}, the quadrupolar coupling constant, C_Q, and the asymmetry of the electric field gradient tensor, η_Q.

	Hyb35					Hyb20				
	A	B	C	D	E	A	B	C	D	E
δ_{QIS} (ppm)	-263.8	-337.6	-339.0	-36.1	-84.8	-174.6	-281.8	-283.7	-27.9	-30.7
	-30.8	-36.1	-35.2	-39.9	-87.4	-18.6	-28.5	-27.6	-31.5	-62.6
		-41.3	-41.9	-21.2	-156.1		-30.0	-30.4	-17.5	-88.8
		-44.1	-26.4	-26.0	-65.8		-33.0	-12.8	-18.7	-33.2
			-44.8	-39.9	-185.3			-38.5	-31.1	-58.3
				-145.9	-33.1				-128.2	-27.8
				-232.8	-301.4				-211.6	-49.0
				-156.4	-41.6				-153.3	-20.6
				-333.5					-279.8	
C_Q (MHz)	-12.2	-13.9	-13.9	-4.0	-6.4	-9.9	-12.7	-12.7	-3.5	-4.0
	-4.1	-4.5	-4.4	-3.8	-6.5	-3.0	-4.0	-3.9	-3.4	-5.3
		-4.8	-4.8	-3.1	-8.9		-4.1	-4.1	-2.8	-6.6
		-5.0	-3.8	-3.3	-5.8		-4.3	-2.6	-2.9	-4.1
			-4.9	-4.5	-9.8			-4.6	-4.0	-5.4
				-8.7	-4.4				-8.2	-3.9
				-10.7	-13.0				-10.3	-4.8
				-9.5	-4.8				-9.4	-3.4
				-13.1					-12.0	
η_Q	0.28	0.24	0.23	0.74	0.56	0.33	0.24	0.24	0.74	0.37
	0.43	0.36	0.36	0.68	0.58	0.47	0.36	0.36	0.69	0.40
		0.35	0.35	0.53	0.66		0.35	0.35	0.57	0.77
		0.33	0.42	0.58	0.61		0.32	0.44	0.56	0.31
			0.44	0.61	0.61			0.49	0.65	0.75
				0.42	0.09				0.38	0.49
				0.63	0.29				0.61	0.44
				0.11	0.30				0.07	0.21
				0.62					0.61	

Table S6: Calculated ^{17}O Hyperfine NMR parameters for NMMO under each of the charge compensation states, A to E, using two levels of hybrid functionals, one with 35% Hartree-Fock exchange (Hyb35) and one with 20% (Hyb20). Calculations are performed assuming a field strength of 11.7 T and an experimental temperature of 318 K (to account for the frictional heating incurred under 60 kHz magic angle spinning). Quantities listed include: the isotropic hyperfine shift, $\delta_{hyp,iso}$, the dipolar hyperfine shift anisotropy, $\Delta\delta_{hyp}$, and the asymmetry of the dipolar hyperfine tensor, η_{hyp}.

			Hyb35					Hyb20		
	A	B	C	D	E	A	B	C	D	E
$\delta_{hyp,iso}$ (ppm)	19575	-12923	-13074	6402	6591	16265	-9088	-9400	7264	4882
	3190	212	194	1766	5417	4712	1749	1735	3072	2675
		-2576	-2637	414	-576		674	581	2477	-291
		-1052	2797	4236	3881		1589	4786	5212	5852
			3489	8327	-1915			3700	9544	-2614
				23346	9626				18942	3605
				-23044	-8987				-16424	3441
				41932	8488				34840	5408
				15362					12829	
$\Delta\delta_{hyp}$ (ppm)	-41908	41647	41636	-4416	816	-38223	39440	39435	-3832	3160
	-10187	-5624	-5644	2381	4030	-12131	-5197	-5280	2094	7966
		-5638	-5687	-2808	24260		-5044	-5070	-2774	20772
		-6289	-11466	-3409	14638		-6008	-13537	-3521	-2007
			-4823	-6588	26292			-5307	-6302	18526
				-9141	-10850				-8649	11059
				26709	35211				21755	15213
				29396	-9446				28961	-2782
				-42048					-39314	
η_{hyp}	0.03	0.05	0.05	0.56	0.46	0.03	0.05	0.06	0.64	0.53
	0.29	0.43	0.46	0.66	0.61	0.17	0.41	0.44	0.72	0.54
		0.52	0.57	0.43	0.20		0.38	0.45	0.31	0.24
		0.61	0.30	0.68	0.67		0.51	0.14	0.57	0.68
			0.37	0.43	0.20			0.38	0.35	0.13
				0.21	0.60				0.26	0.48
				0.37	0.09				0.60	0.52
				0.03	0.72				0.04	0.66
				0.20					0.23	

Using Hyperfine Basis Sets for Shifts of Paramagnetic Oxygen

To confirm whether the basis sets used for paramagnetic NMR calculations were appropriate for determining the shifts of paramagnetic O centres, calculations of the hyperfine coupling constants to ^{17}O for both triplet oxygen (O_2*) and a hydroxyl radical (•OH) were calculated; the calculated parameters are given in Table S7 and compared to observed and calculated ^{17}O EPR parameters for these species.[44,45] The match between the calculated and observed parameters is generally good; it should be noted that the deviation seen for O_2* likely stems from the presence of low-lying excited states which are over-accounted for.

Table S7: Calculated and observed ^{17}O isotropic hyperfine parameters, A_{iso}, for O_2* and •OH. [a] from ref. 44 and [b] from ref. 45

	Observed A_{iso} (MHz)	Calculated A_{iso} (MHz)
O_2*	−56.7[a]	−24.3
•OH	−24.3[b]	−23.9

Benchmarking CRYSTAL DOS Calculations with VASP

Several studies in the literature use VASP (a plane-wave code) for calculating the density of states of paramagnetic battery cathode materials at the end of charge.[46,47] To compare the results obtained in this work against those of previous works on related materials, the densities of states (DOSs) of NMMO were computed in both CRYSTAL (using the Hyb35 and Hyb20 functionals used for shift calculations) and VASP (using DFT+U, as per the experimental). These are shown in Figure S1. The DOSs generated by CRYSTAL have similar features to those in VASP, with O-dominated states just below E_F. Notably, VASP calculates states which are dominated by O also just above E_F, whereas CRYSTAL predicts that Mn dominates these states (the latter in line with expectation from simple crystal field considerations). In addition, both Mn and O contribute similar amounts to states below E_F for the VASP DOS, but O overwhelmingly dominates these states in the CRYSTAL DOSs, the latter again consistent with expectation from crystal field theory. It can therefore be argued that, on the basis of these DOSs and the success of these functionals for calculating the hyperfine shifts and magnetic properties of paramagnetic cathode materials, CRYSTAL is better suited to DOS calculations than VASP for this system. Based on the better match of the Hyb20 band gap to that from VASP, all calculations presented below are Hyb20.

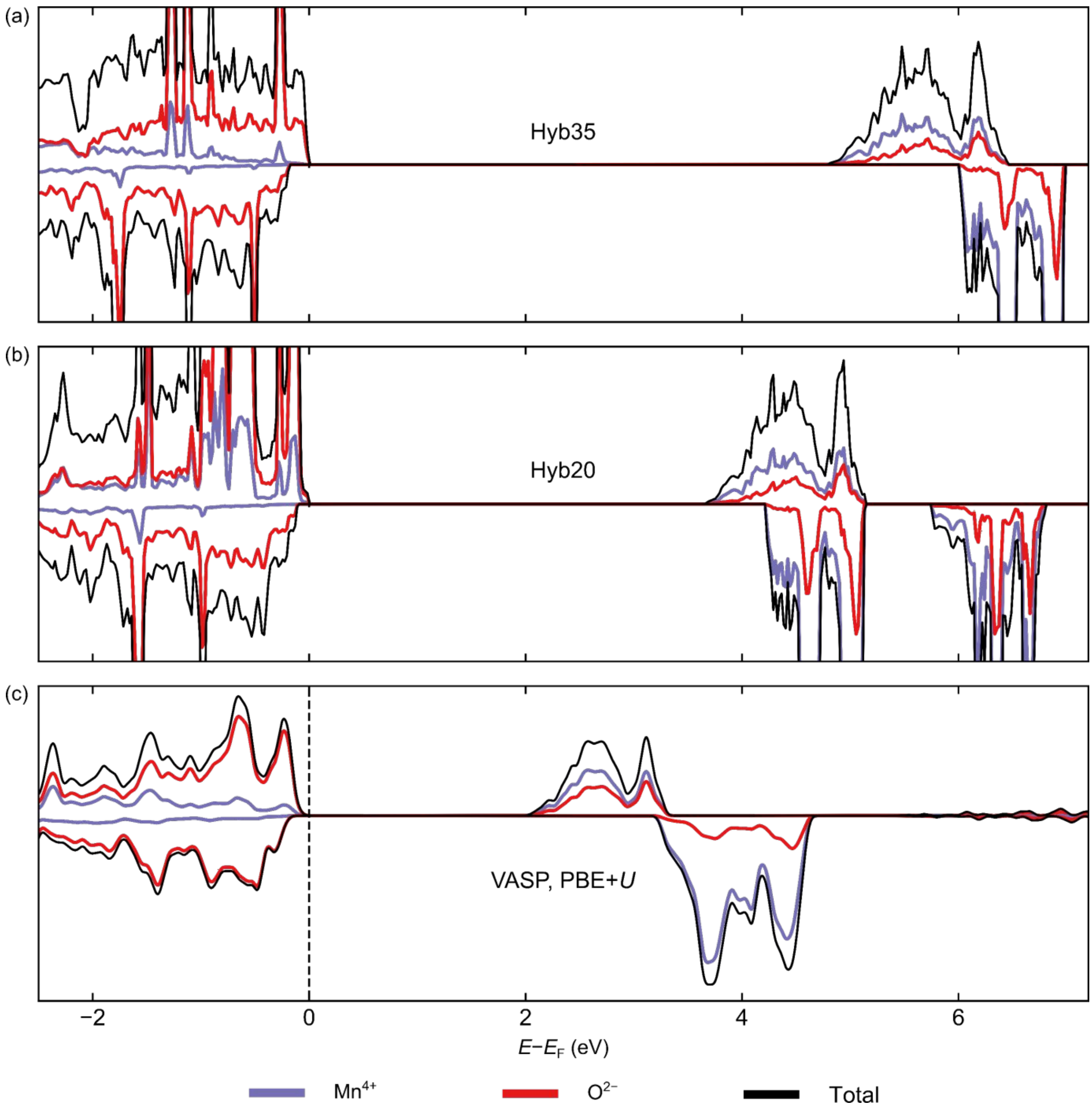

Figure S1: DOS calculations for pristine NMMO using **(a)** the CRYSTAL code with Hyb35 functional, **(b)** the CRYSTAL code with Hyb20 functional and **(c)** the VASP code with DFT+U. The spin-up bands are shown as positive and the spin-down as negative.

Antiferromagnetic Arrangements

The relative energies of the different charge compensation schemes detailed in the main text depend intimately on the magnetic arrangement of each system. In the calculations presented in the main text, a ferromagnetic ground state was assumed (whilst this is not necessarily the true ground state, this method is successful for calculating shifts). In Figure S2, the DOS of the system when in an antiferromagnetic ground state are compared. It should be noted that systems antiferromagnetic A produces incredibly similar results (in terms of the DOS and cell energy) to that of ferromagnetic B and *vice versa*.

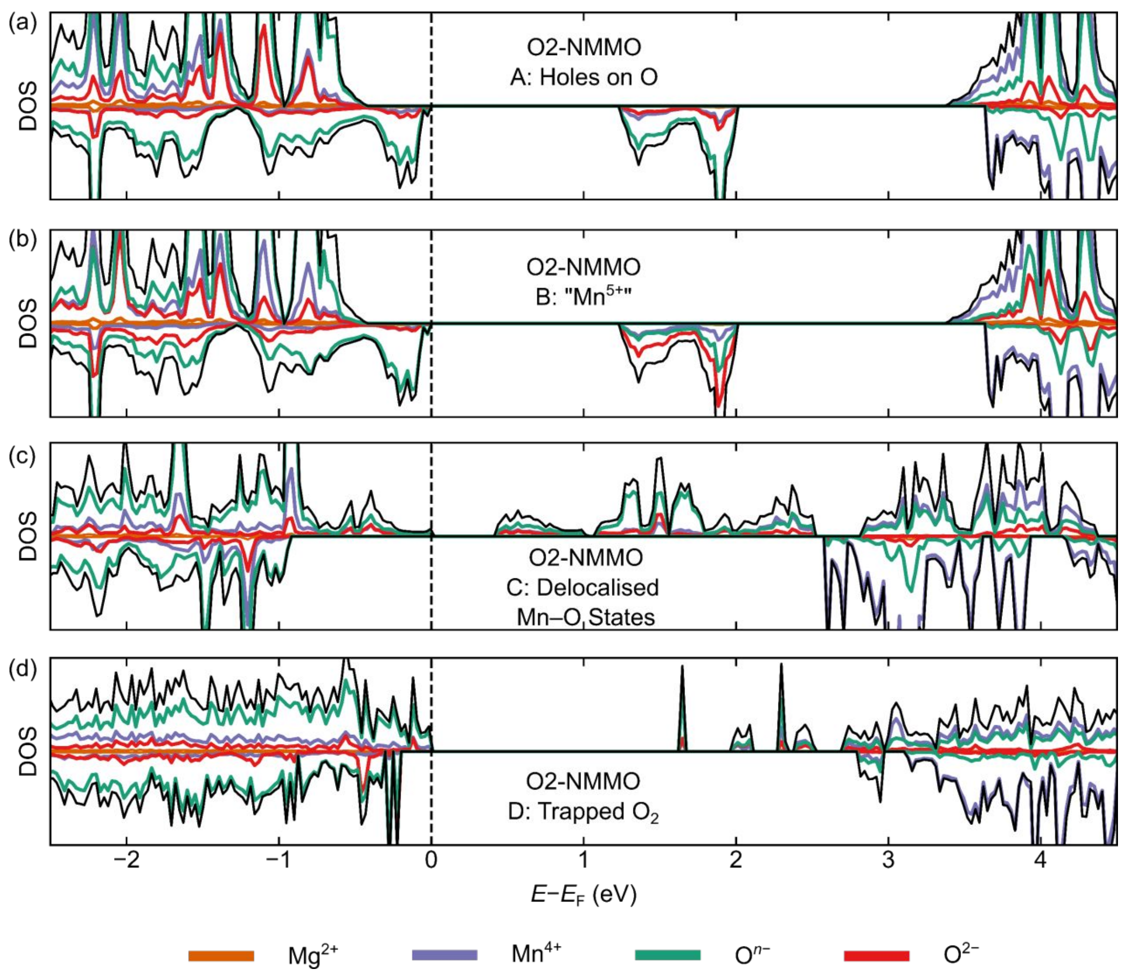

Figure S2: DOS calculations for antiferromagnetic arrangements of O2-NMMO under **(a)** scheme A, **(b)** scheme B, **(c)** scheme C and **(d)** scheme D. DOSs are plotted with spin up bands as positive and spin down bands as negative. Note that these DOSs are calculated using the Hyb20 functional.

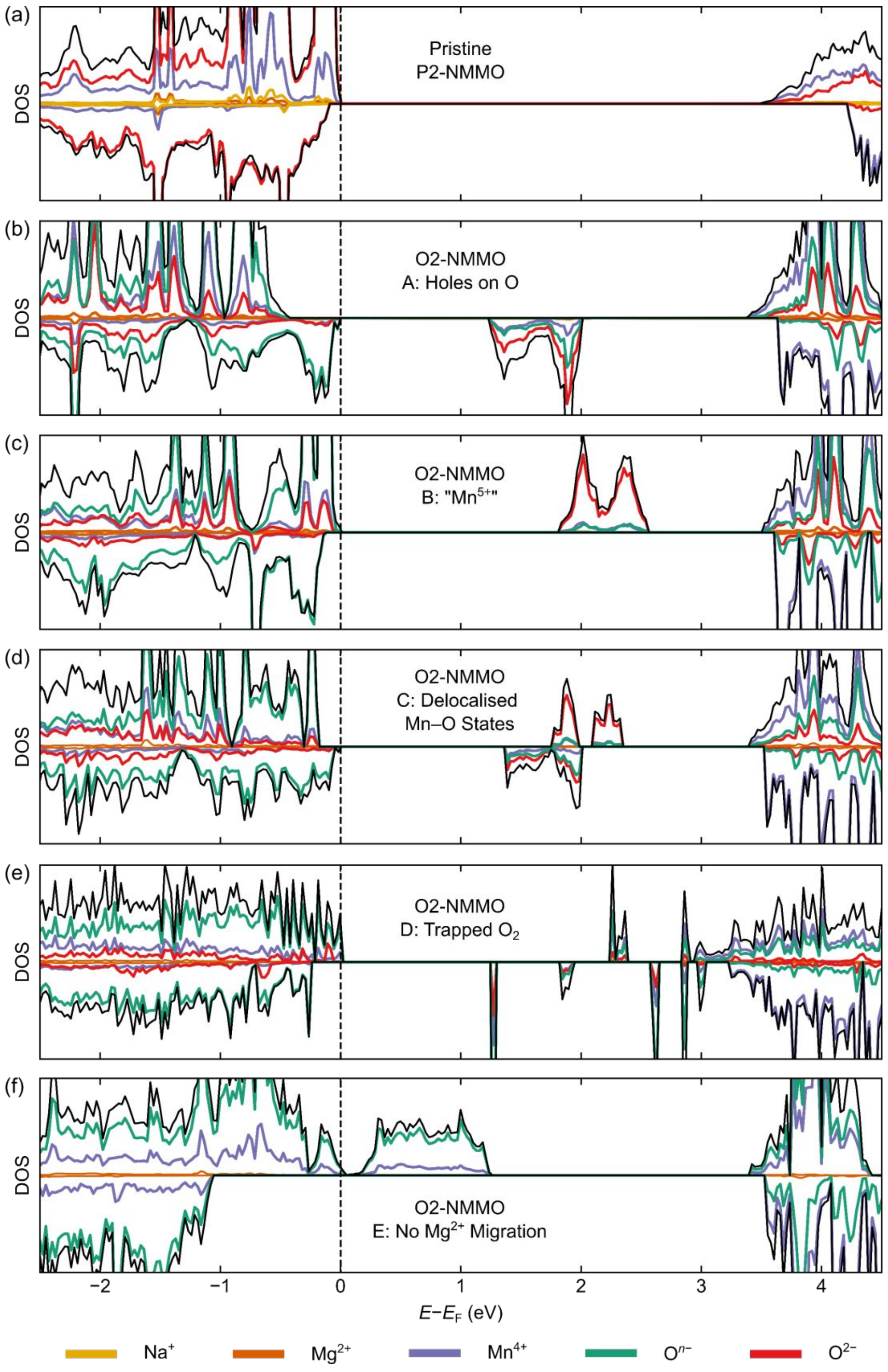

Figure S3: Ab initio density of states calculations for each of the O2-NMMO charge compensation models: (a) shows the pristine DOS, whilst (b) to (f) show the DOSs for schemes A to E, respectively. DOSs were calculated using a hybrid DFT functional with 20% Hartree-Fock. The upward- and downward-facing DOSs correspond to the spin up and down bands.

Magnetic Cluster Expansion Results

To examine the magnetic properties (exchange constants) of NMMO under different charge compensation schemes, we performed a magnetic cluster expansion of NMMO in charge compensation schemes B and D. The energies were fit against a simple Ising Hamiltonian:

$$\hat{H} = \sum_{i,j} J_{ij} S_i S_j \, ,$$

where J_{ij} is the exchange constant between the ith and j^{th} spins, S_i and S_j. Note that negative J_{ij} correspond to antiferromagnetic interactions, while positive values are ferromagnetic. The Lasso linear regression model was used to fit the energies, with an optimized *α*-value of 1 μeV; the fits are shown in Figure S4. The reference state for B was Mn^{4+} with antiferromagnetically aligned O^{-} (the ground state for this system), while the reference for D was Mn^{4+} and O_2 ferromagnetically aligned (an excited state; the ground state was when the spin on O_2 was anti-parallel to Mn^{4+}). We note that these calculations employed the r^2SCAN functional; for clarity, we have plotted the energies of states B and D using four different functionals, Hyb35 and Hyb20 (implemented in CRYSTAL), and PBE+*U* and r^2SCAN (implemented in VASP) in Figure S5.

Only the nearest-neighbour exchange constants were included in the fits, as these were the minimum required to fit the data; adding additional clusters did not improve the fit. The exchange coefficients are tabulated in Table S8.

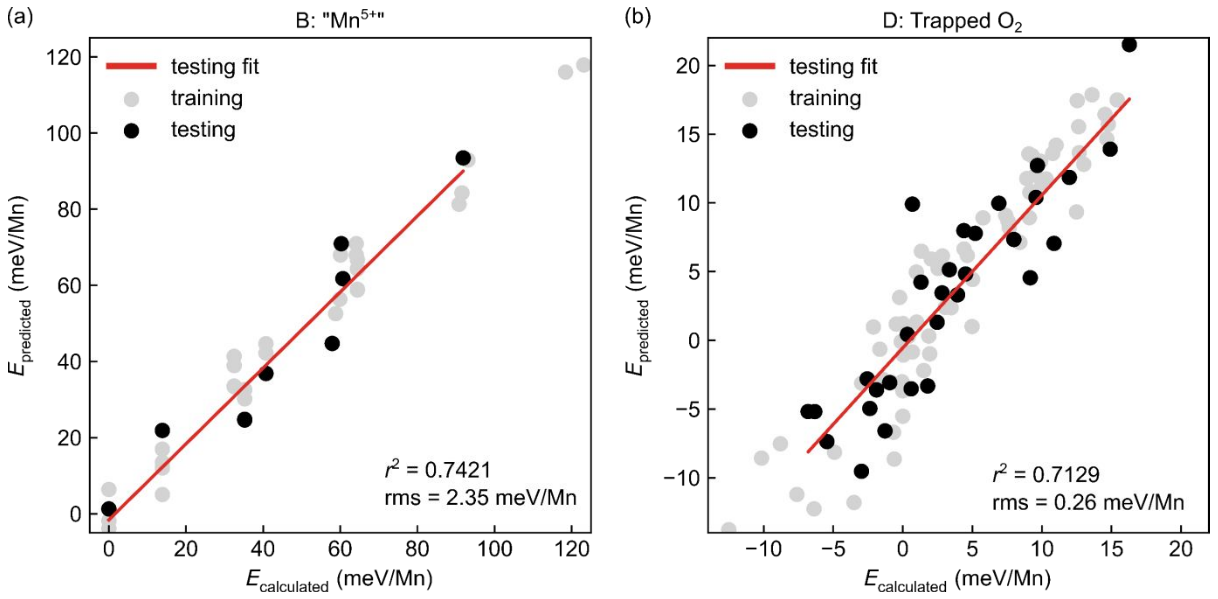

Figure S4: Magnetic cluster expansion fits for NMMO under charge compensation states **(a)** B and **(b)** D.

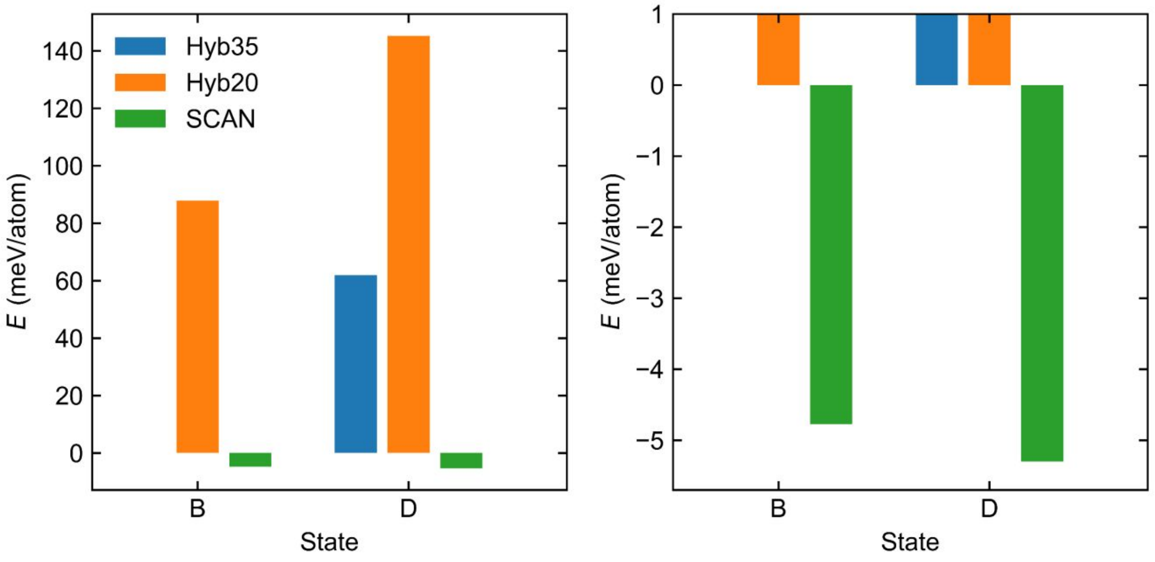

Figure S5: Comparison of the energies of states B and D using Hybrid 20 and 35 (from CRYSTAL calculations), as well as PBE+*U* and r^2SCAN (implemented in VASP). On the right, an zoom-in on the low-energy region is shown. Zero on these energy scales corresponds to B computed with the Hyb35 functional.

Table S8: Nearest-neighbour magnetic exchange constants, J, obtained from the magnetic cluster expansions of charge compensation states B and D. Note that J_{Mn-O} represents the exchange constant between Mn^{4+} and a nearby O^{n-} ($n \approx 1$ for B, $1.0 < n < 2.0$ for D), while J_{Mn-O_2} is the exchange constant between Mn^{4+} and O_2 molecules.

J (K)	B	D
J_{Mn-Mn}	+5.2	+1.0
J_{Mn-O}	−196.9	−5.6
J_{O-O}	+1.9	+7.3
J_{Mn-O_2}	n/a	−2.7

3. Additional ^{25}Mg NMR Results

Probe Background

In addition to the resonances observed from the sample, additional background features were seen in the ^{25}Mg NMR spectra, due to the Macor (Mg-containing ceramic) contained in the probe. This background is seen in Figure S6.

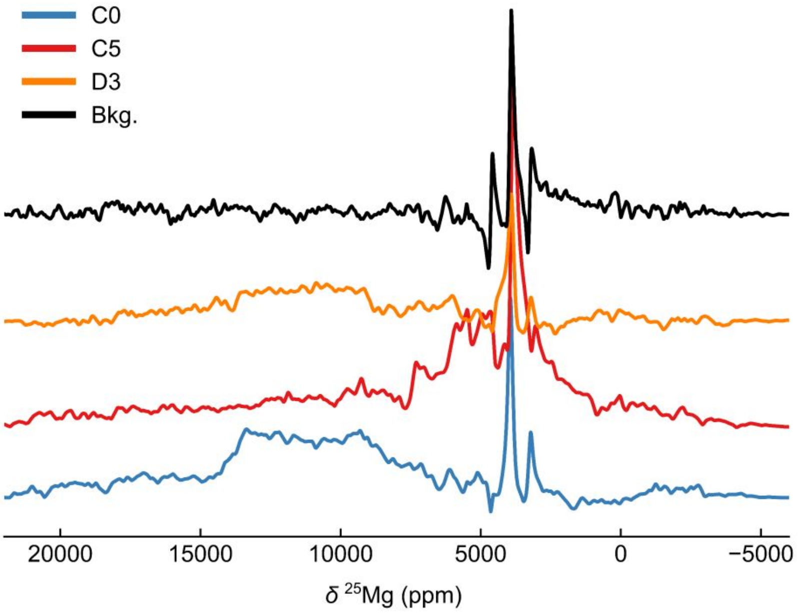

Figure S6: Ex situ ^{25}Mg NMR spectra of NMMO at different states of charge, with the probe background (empty stator and rotor).

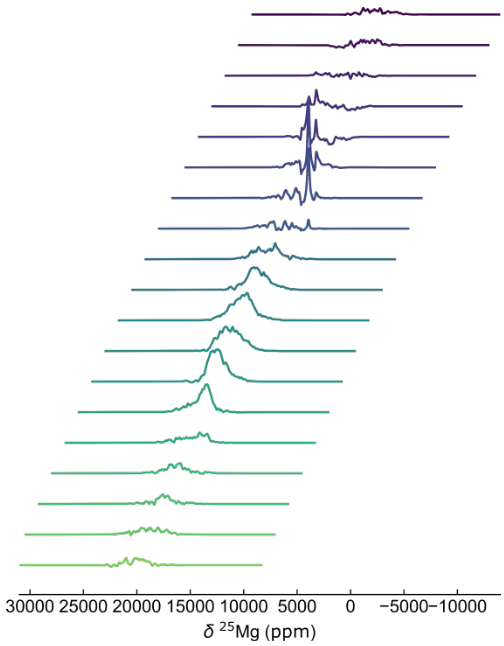

Figure S7: VOCS ^{25}Mg NMR spectrum of pristine NMMO (C0), recorded at 16.4 T, static (T = 300 K). The lowest receiver offset was −2500 ppm and the highest 20000 ppm, in steps of 1250 ppm.

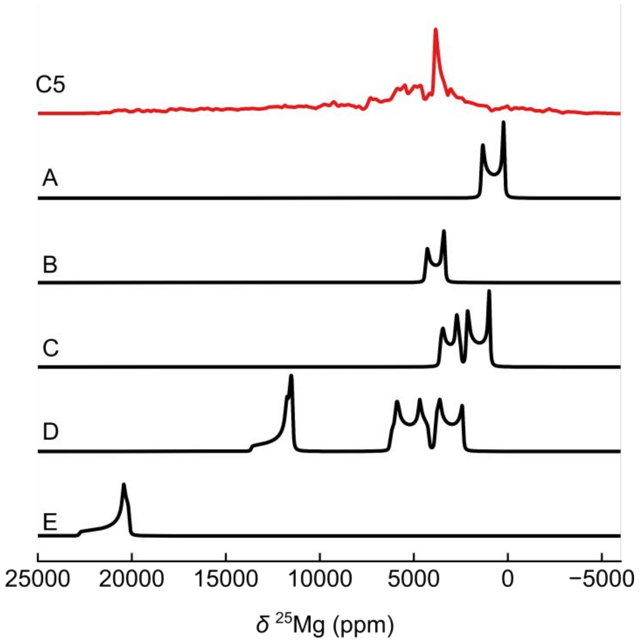

Figure S8: Comparison of ^{25}Mg NMR spectrum of NMMO at the end of first charge (C5), with simulated spectra corresponding to each of the five possible charge compensation states, A to E.

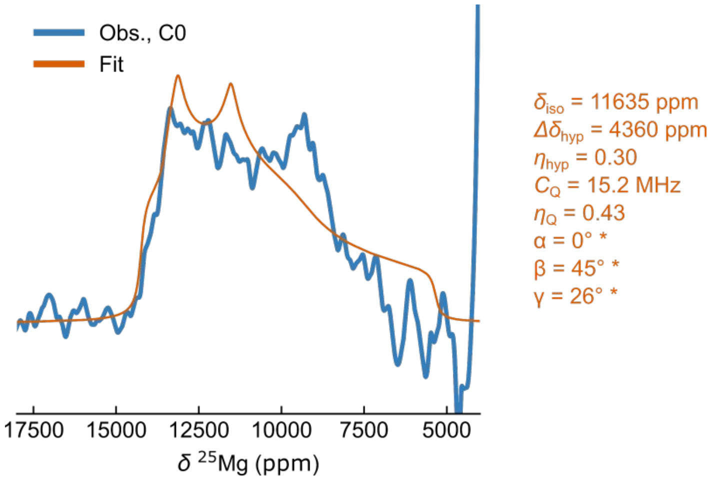

Figure S9: Fit to the ^{25}Mg NMR spectrum of pristine NMMO (C0) using a quadrupolar and chemical shift anisotropy model. Fitted values are highlighted to the right. Asterisks denote values which were frozen during the fit.

Additional fits to the ^{25}Mg NMR spectrum of pristine NMMO using only a single site yield a fit of similar quality [Figure S9], but with fitted parameters which are much less reasonable compared to that shown in the main text. We also argue that, given that there are approximately 0.11 equivalents of Mn^{3+} in the pristine material, we expect at least two environments: one which is Mn^{4+}-rich and one which is less Mn^{4+}-rich and more Mn^{3+}-rich. This is also in accordance with our previous work, where we identified two EPR resonances

in pristine NMMO: one from a Mn^{4+}-only environment and one with a mixed Mn^{3+} and Mn^{4+} environment.

4. Additional ^{17}O NMR Results

pjMATPASS Experiments

For all samples, a ^{17}O NMR pjMATPASS experiment was run to isolate the isotropic resonance from the spinning sideband manifold. Where a distinct set of resonances was observed in the *ex situ* Hahn-Echo VOCS data for NMMO [Figure S10], the pjMATPASS successfully identified the isotropic resonance; in all other cases, the isotropic resonance could not be identified, suggesting that these spectra either comprise several, broad, overlapping resonances (which are so broad that they become lost to the baseline) or that the T_2 nuclear and/or T_{1e} electronic relaxation times are severely shortened, giving a broad feature.

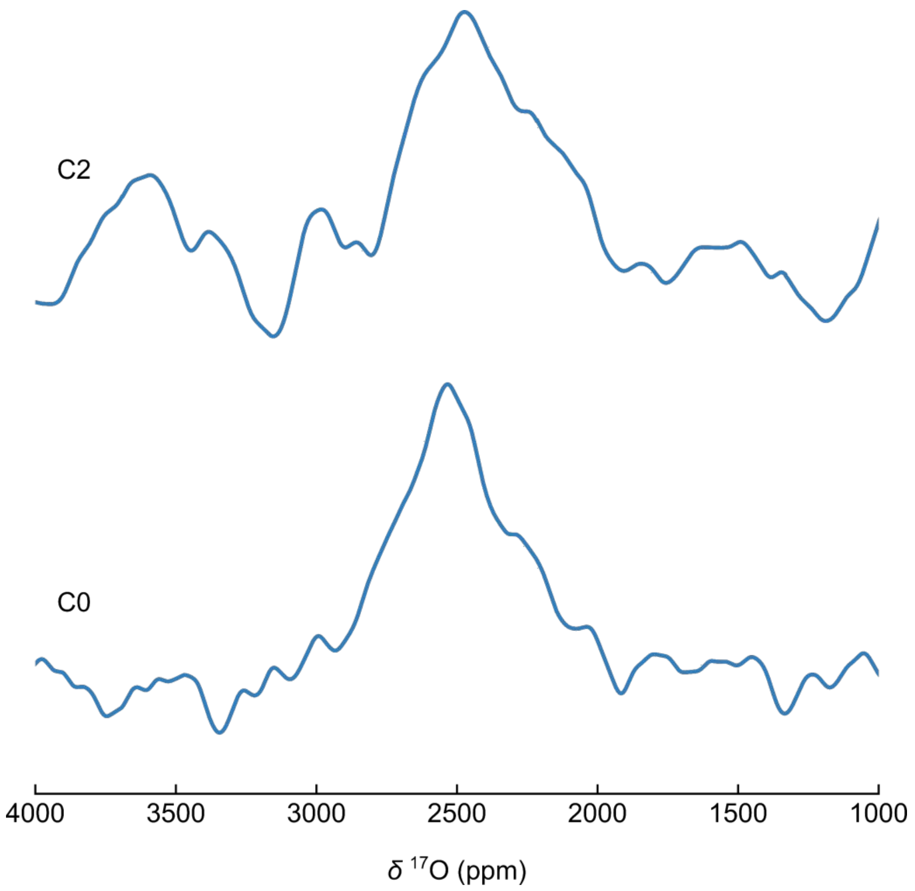

Figure S10: Isotropic slice of the pjMATPASS experiments for *ex situ* samples C0 and C2 of NMMO, acquired at 11.7 T under 60 kHz MAS rate.

Fits of Pristine ^{17}O NMR Spectrum at 16.4 T

To determine the effect of the quadrupolar interaction on the ^{17}O NMR spectrum of pristine NMMO, Hahn-echo VOCS data was acquired at two fields and fit; these fits are shown in Figure S11. The isotropic shifts of each of the sharp peaks undergo little change; the broad resonance, however, changes shift substantially. This change in shift is ascribed to the change in the difference frequencies of the Mn^{3+}–^{17}O environments: the dynamic JT distortion of Mn^{3+} results in O changing shift from an environment bound to Mn^{3+} *via* a short bond to one with a long bond. On increasing the field strength, the absolute frequency difference increases, such that ^{17}O is less effectively dynamically averaged and the peak shifts. It is also possible that only a small proportion of the O-$(Mn^{3+})_x$ sites are observed, due to faster relaxation times at higher field and/or because some of these sites were in the intermediate site exchange regime where no distinct resonances are seen. The fitted hyperfine and quadrupolar parameters for the fits are given in Table S9.

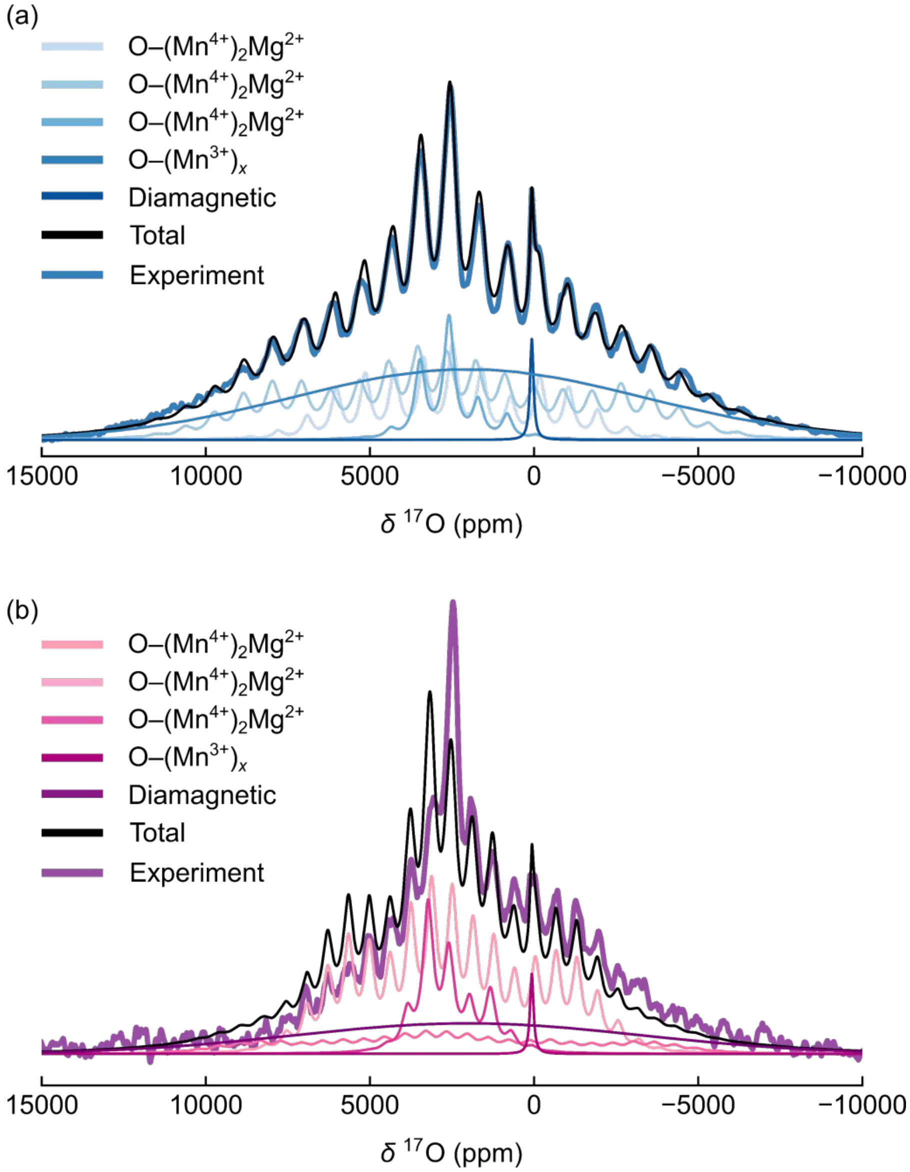

Figure S11: Fits to the ^{17}O NMR spectra acquired under 60 kHz MAS and at **(a)** 11.7 T and **(b)** 16.4 T.

Table S9: Fitted ^{17}O hyperfine and quadrupolar parameters from the fits at 11.7 T and 16.4 T. Note that all sites except the O–$(Mn^{3+})_x$ site were fit using combined chemical shift anisotropy and quadrupolar models; the O–$(Mn^{3+})_x$ site was fit to a Gaussian/Lorentzian peakshape. Values listed include: the isotropic hyperfine shift, δ_{iso}, the dipolar hyperfine shift anisotropy, $\Delta\delta_{hyp}$, the asymmetry of the dipolar hyperfine tensor, η_{hyp}, the quadrupolar coupling constant, C_Q, and the asymmetry of the electric field gradient tensor, η_Q.

11.7 T

Site	δ_{iso} (ppm)	$\Delta\delta_{hyp.}$ (ppm)	$\eta_{hyp.}$	C_Q (MHz)	η_Q
O–$(Mn^{4+})_2Mg^{2+}$	2490(30)	−5210(50)	0.85(2)	3.57(3)	0.48(2)
O–$(Mn^{4+})_2Mg^{2+}$	2660(20)	−8140(20)	0.91(2)	3.65(2)	0.30(3)
O–$(Mn^{4+})_2Mg^{2+}$	2590(20)	−2030(25)	0.07(2)	3.48(3)	0.60(2)
O–$(Mn^{3+})_x$	2020(350)	-	-	-	-

16.4 T

Site	δ_{iso} (ppm)	$\Delta\delta_{hyp.}$ (ppm)	$\eta_{hyp.}$	C_Q (MHz)	η_Q
O–$(Mn^{4+})_2Mg^{2+}$	2490(70)	−5210(50)	0.85(2)	3.57(3)	0.48(2)
O–$(Mn^{4+})_2Mg^{2+}$	2660(60)	−8140(50)	0.91(2)	3.75(2)	0.45(2)
O–$(Mn^{4+})_2Mg^{2+}$	2380(40)	−2030(60)	0.07(2)	3.48(3)	0.60(2)
O–$(Mn^{3+})_x$	2020(190)	-	-	-	-

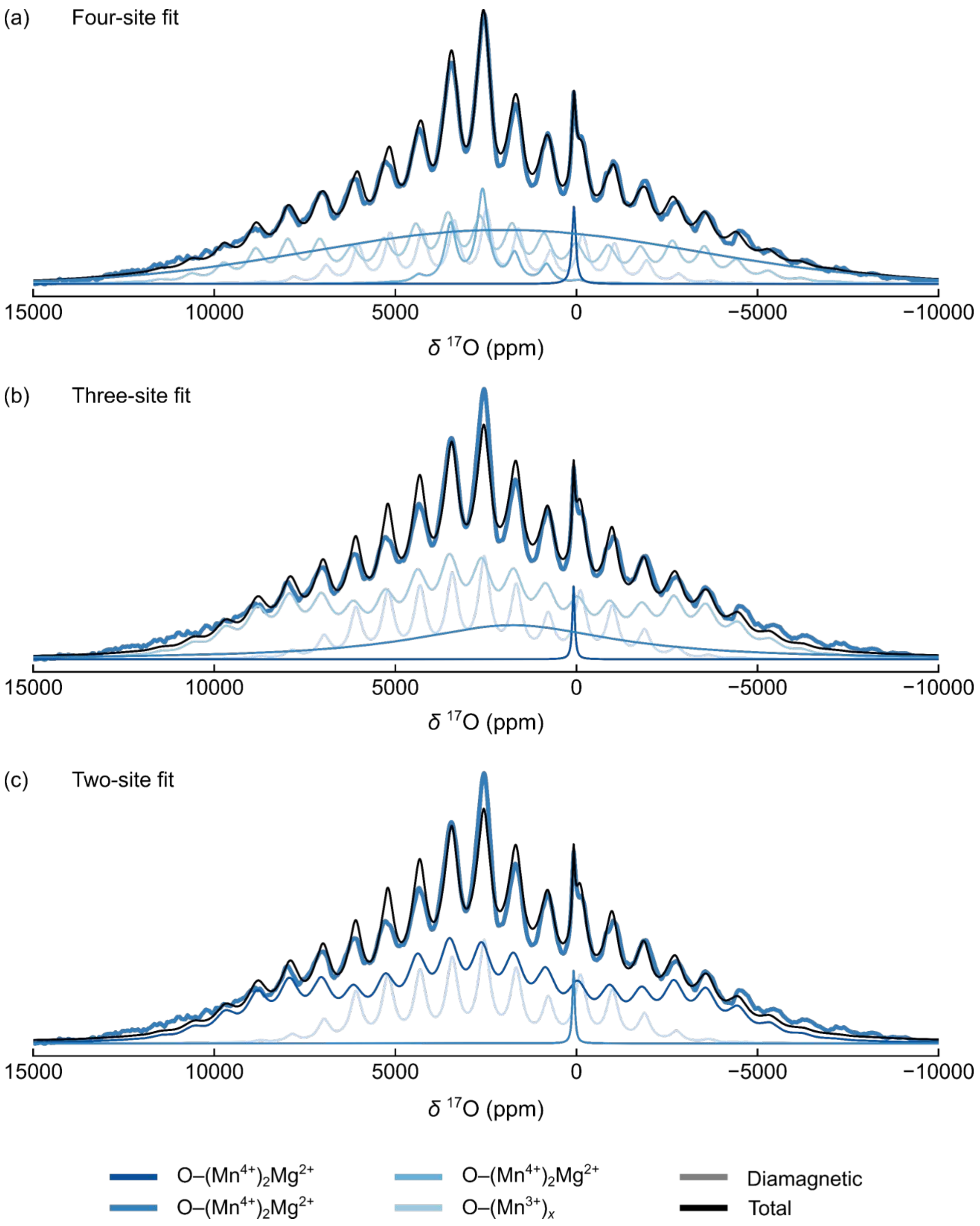

Figure S12: Fits to the ^{17}O NMR spectra acquired under 60 kHz MAS and at 11.7 with **(a)** four sites, **(b)** three sites and **(c)** two sites.

The fit to the ^{17}O NMR spectrum of pristine NMMO was fit using a model containing four paramagnetic O sites; fits with fewer components yielded a poorer match to the data. The four-site model may also be rationalised based on the structure of NMMO: we anticipate there to be four crystallographically unique sites for O^{2-} in the honeycomb-ordered supercell and one in the parent (disordered) cell identified in our previous work.[2] These correspond to subtle distortions in the bond angles and lengths around O. On this basis, we anticipate that our four-site model with three sites of similar shifts (2490, 2590 and 2660 ppm) to correspond to subtle changes in the bond angles and lengths. The broad resonance, assigned to O bound to any number of Mn^{3+}, is justified by the presence of 0.11 equivalents of Mn^{3+} on the transition metal sublattice in pristine NMMO.

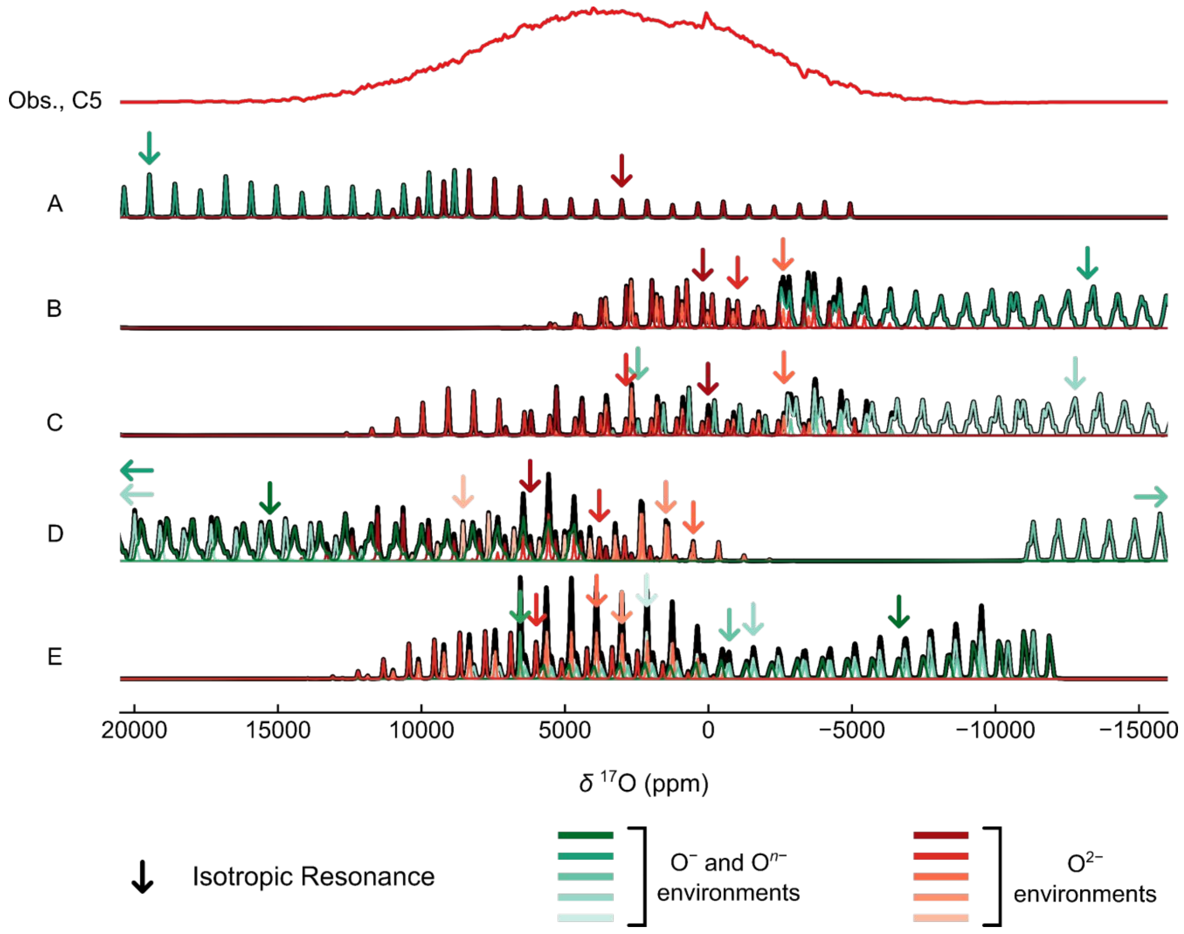

Figure S13: Comparison of the *ex situ* ^{17}O NMR spectrum acquired at point C5 of ^{17}O-enriched NMMO (at 11.7 T and 60 kHz MAS) with simulated spectra for each of the charge compensation mechanisms considered in this work. Arrows which face one side or the other in spectrum D indicate isotropic resonances beyond the plot window shown here.

5. Solution-State NMR Results

^{19}F NMR spectra were used to assess the decomposition of $NaPF_6$ and they showed that HF is not formed (it would show as a peak at -156 ppm in the ^{19}F spectrum).

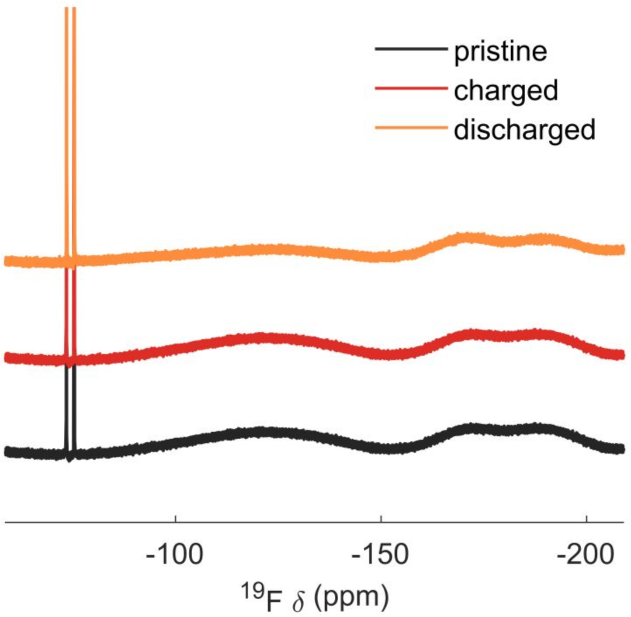

Figure S14: Solution-state ^{19}F NMR spectra for the electrolyte used in NMMO half cells. Pristine corresponds to fresh electrolyte (which had been soaked in a coin cell but not been electrochemically cycled); "charged" corresponds to point C5, while discharged is point D3. The oscillatory features between approximately −120 ppm and −200 ppm correspond to background from the probe.

The ^1H NMR spectra for all samples show the PC peaks at 1.4 ppm (d, J = 6.2Hz, $-CH_3$), 4.0 ppm (dd, 8.5 Hz, 7.0 Hz), 4.53 ppm (dd, 8.5 Hz, 7.8 Hz) and 4.83 ppm (dqd, 8.5 Hz, 6.2 Hz). Water is usually expected as a singlet at 2.46 ppm,[48] but it is absent here and consistent with the absence of HF. Acetone is one of the major decomposition products deriving from oxidation of PC and is present as a singlet at 2.07 ppm.[49] Peaks below 0.2 ppm are likely resulting from reaction of the electrolyte with the glass fiber separator (Si-containing species).

Additional resonances that do not appear in the pristine electrolyte spectrum: 3.90 ppm (charge), 3.87 ppm (dis), 3.69 ppm (m, both), likely ascribed to some form of cyclic ether;[49] 3.27 ppm and 3.37 ppm (m, both), likely from the propylene oxide intermediate reported by Arakawa and coworkers;[50] a shift in the singlet resonance from 2.91 ppm in the pristine spectrum to 2.93 ppm (s, charge), 3.00 ppm (s, disch) could be assigned tentatively to labile -OH groups in decomposition products. Decomposition of PC to propanal is not observed here as the characteristic aldehyde resonance at 10 ppm is absent.

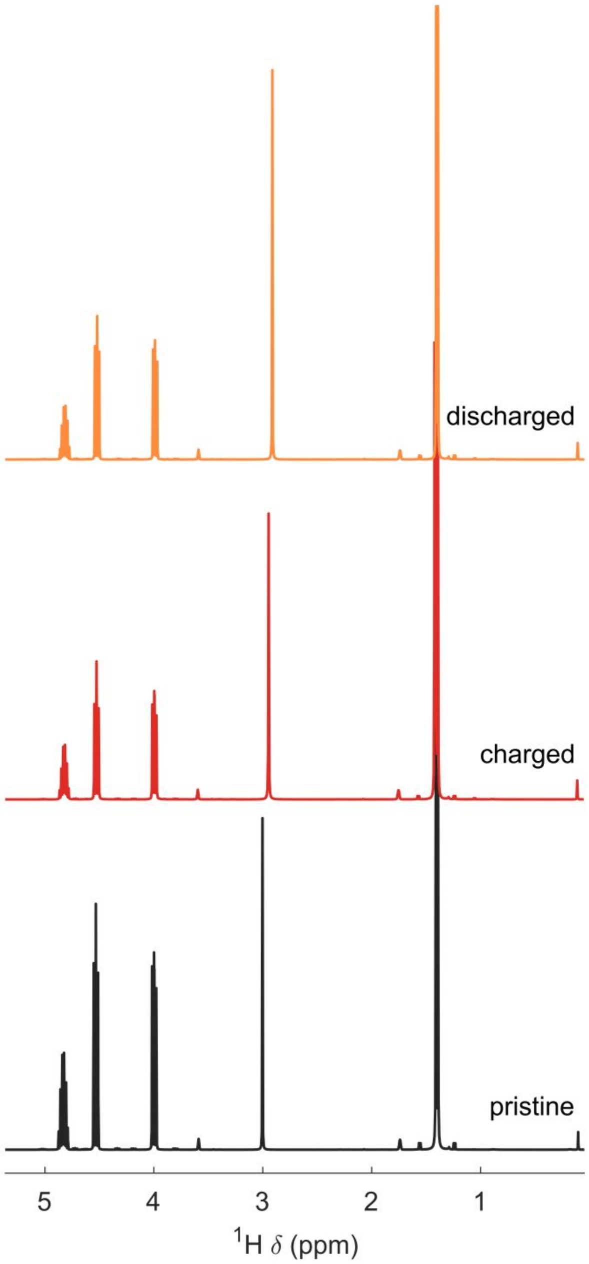

Figure S15: Solution-state ^{1}H NMR spectra for the electrolyte used in NMMO half cells. Pristine corresponds to fresh electrolyte (which had been soaked in a coin cell but not been electrochemically cycled); "charged" corresponds to point C5, while discharged is point D3.

The ^{17}O spectra showed more intense and better resolved signals for the electrolyte extracted from the enriched cathode on discharge compared to the unenriched cell, suggesting diffusion of ^{17}O from the enriched electrode into the electrolyte during cycling. The resonance at 19.00 ppm is due to the THF solvent; the resonance at 225.1 ppm is due to the carbonyl group in PC, while the signal at 115.10 ppm corresponds to the -**O**CH_2 environment and the signal at 139.10 ppm is assigned to the -**O**CCH_3 environment. A weaker signal at 241.60 ppm is likely some form of carbonate, while the weak signal at -15 ppm is likely caused by the propylene

oxide intermediate possibly seen in the ^1H spectra. The weak signal at 81 ppm could be tentatively assigned to solvated CO_2 which is released upon oxidation of PC, following the assignment by Rinkel and coworkers.[51]

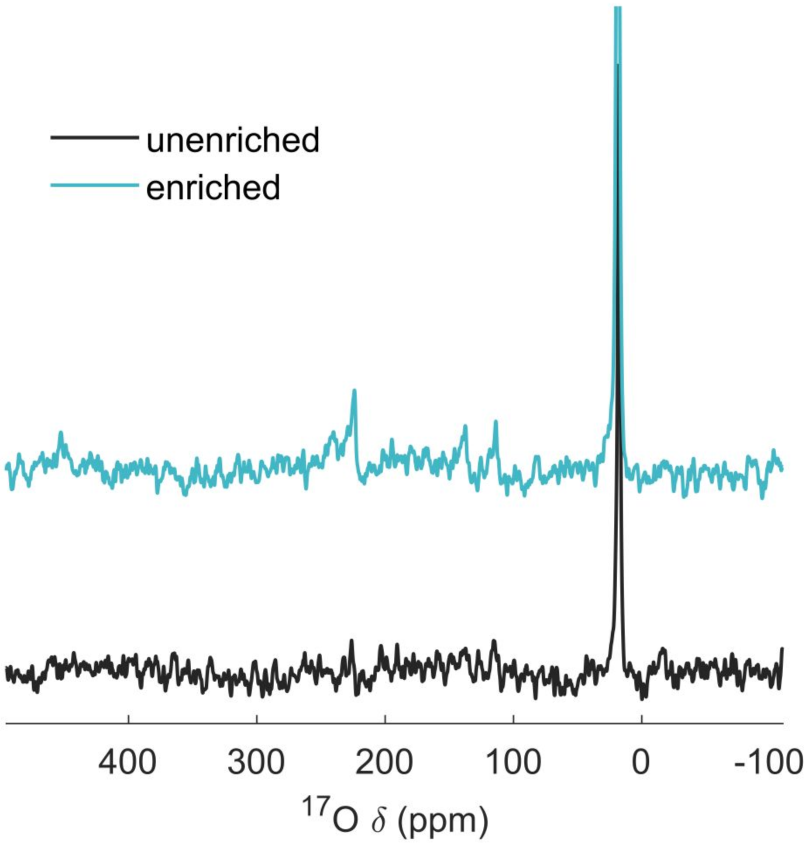

Figure S16: Solution-state ^{17}O NMR spectra for the electrolyte used in NMMO half cells; the electrolyte was harvested from the same state of charge (end of discharge, D4). The "enriched" sample corresponds to electrolyte extracted from a cell where the cathode was ^{17}O-enriched (but the electrolyte was not), while the "unenriched" sample corresponds to electrolyte from an unenriched cathode (this electrolyte was also not enriched).

6. Additional Bulk Magnetic Susceptibility Measurements

***M*(*H*) Curves**

To check whether the low-field approximation for susceptibility held for each of the *ex situ* samples examined and to identify any field-induced magnetic phase transitions, isothermal field-dependent magnetisation curves ($M(H)$ curves) for each sample were recorded [Figure S17 and Figure S18]. In all cases, no hysteresis was observed, no magnetic phase transitions and a linear variation in M with H was seen in the region around H = 0.1 T (the field at which field cooled and zero-field cooled magnetic susceptibility data was recorded), indicating that the small-field approximation was valid.

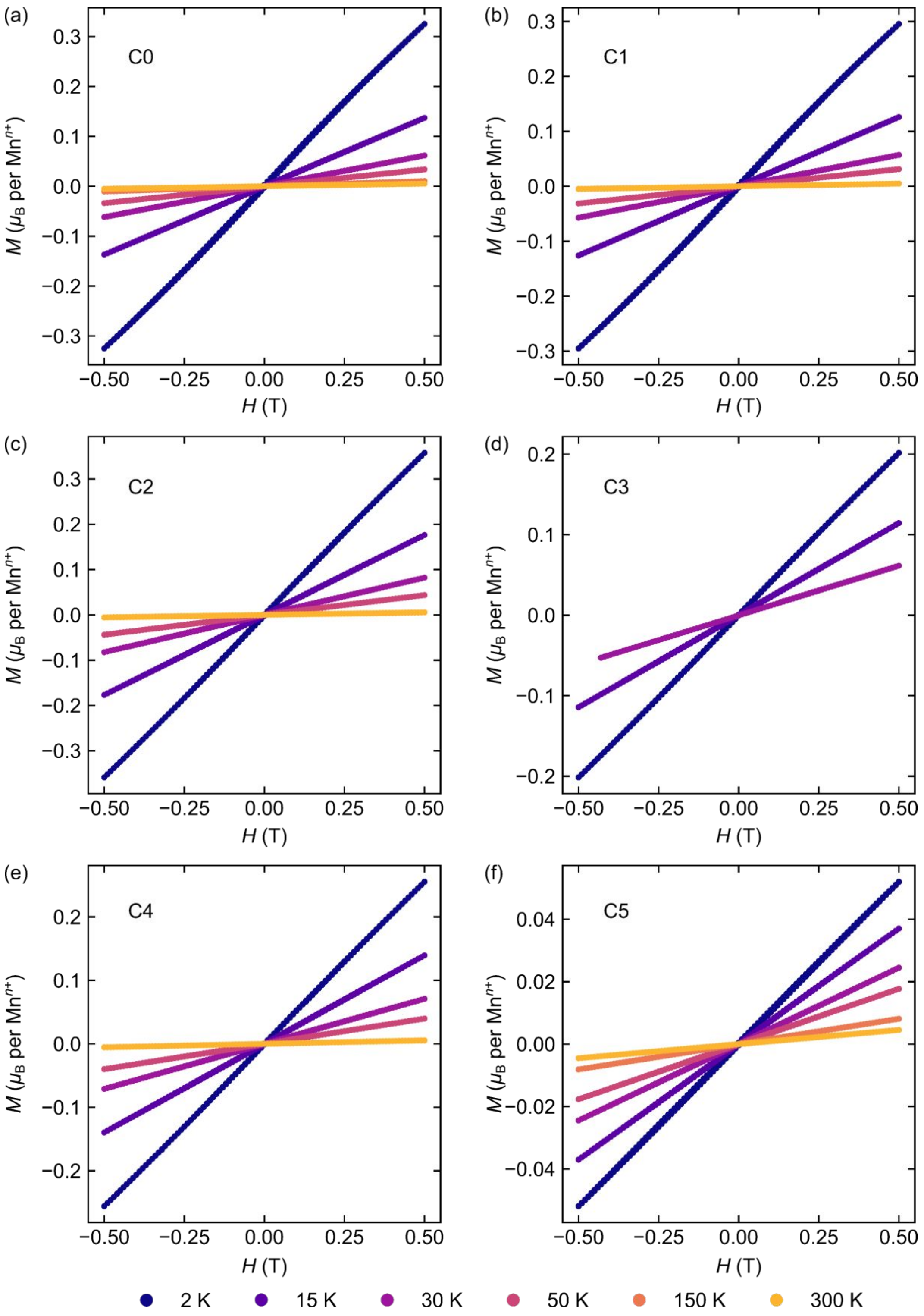

Figure S17: M(H) curves for ex situ samples of NMMO between 2 and 300 K over a field range −0.5 T to +0.5 T: **(a)** C0, **(b)** C1, **(c)** C2, **(d)** C3, **(e)** C4 and **(f)** C5.

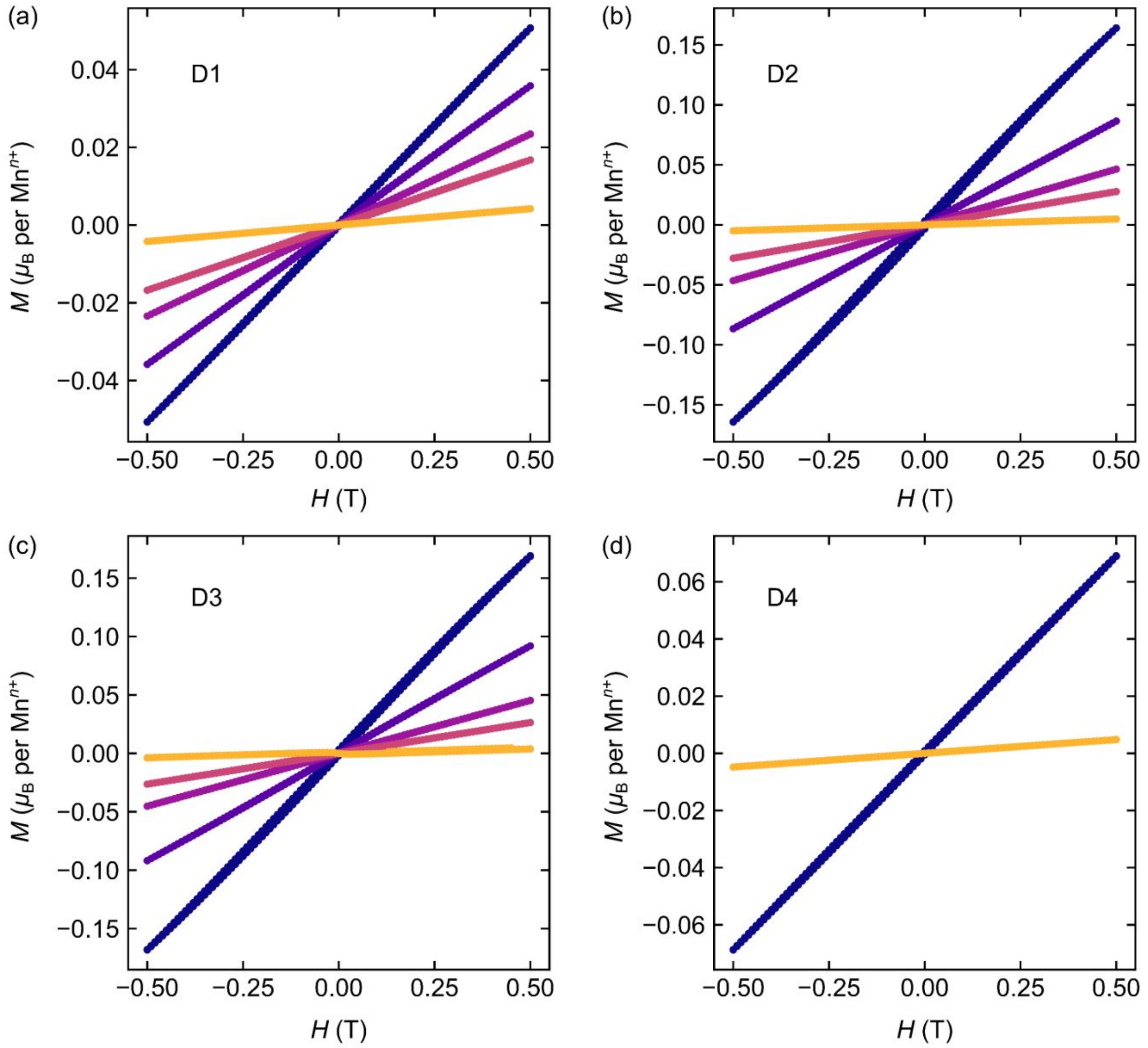

Figure S18: M(H) curves for ex situ samples of NMMO between 2 and 300 K over a field range −0.5 T to +0.5 T: **(a)** D1, **(b)** D2, **(c)** D3 and **(d)** D4.

AC Susceptibility

AC susceptibility data for pristine NMMO and NMMO at the end of charge (i.e., point C5) was collected to investigate their spin-glass-like behaviour. Figure S19 shows the AC susceptibility data acquired for these materials; peaks in the imaginary component (indicative of spin reorientation and precession and hence of spin-glass behaviour) are seen at 6 K for pristine NMMO and at 11 K for NMMO at the end of charge. The increase in the temperature at which spin freezing is seen suggests stronger net exchange interactions experienced by the spins.

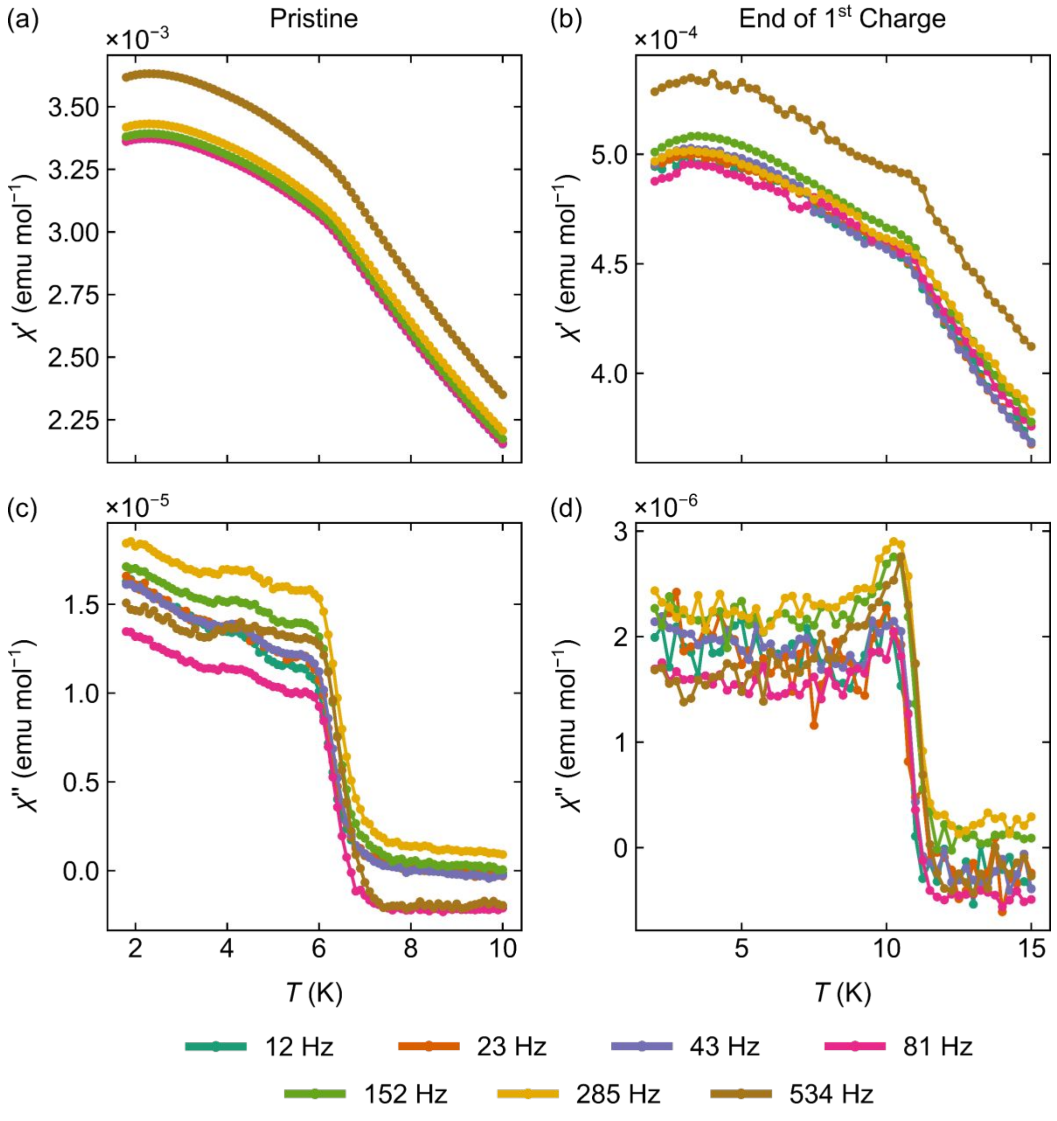

Figure S19: AC Magnetic Susceptibility data for pristine NMMO. **(a)** and **(c)** show the real (χ') and imaginary (χ'') components of the AC susceptibility for pristine NMMO, respectively, whilst **(b)** and **(d)** show the real and imaginary components of the AC susceptibility for NMMO at the end of charge, respectively.

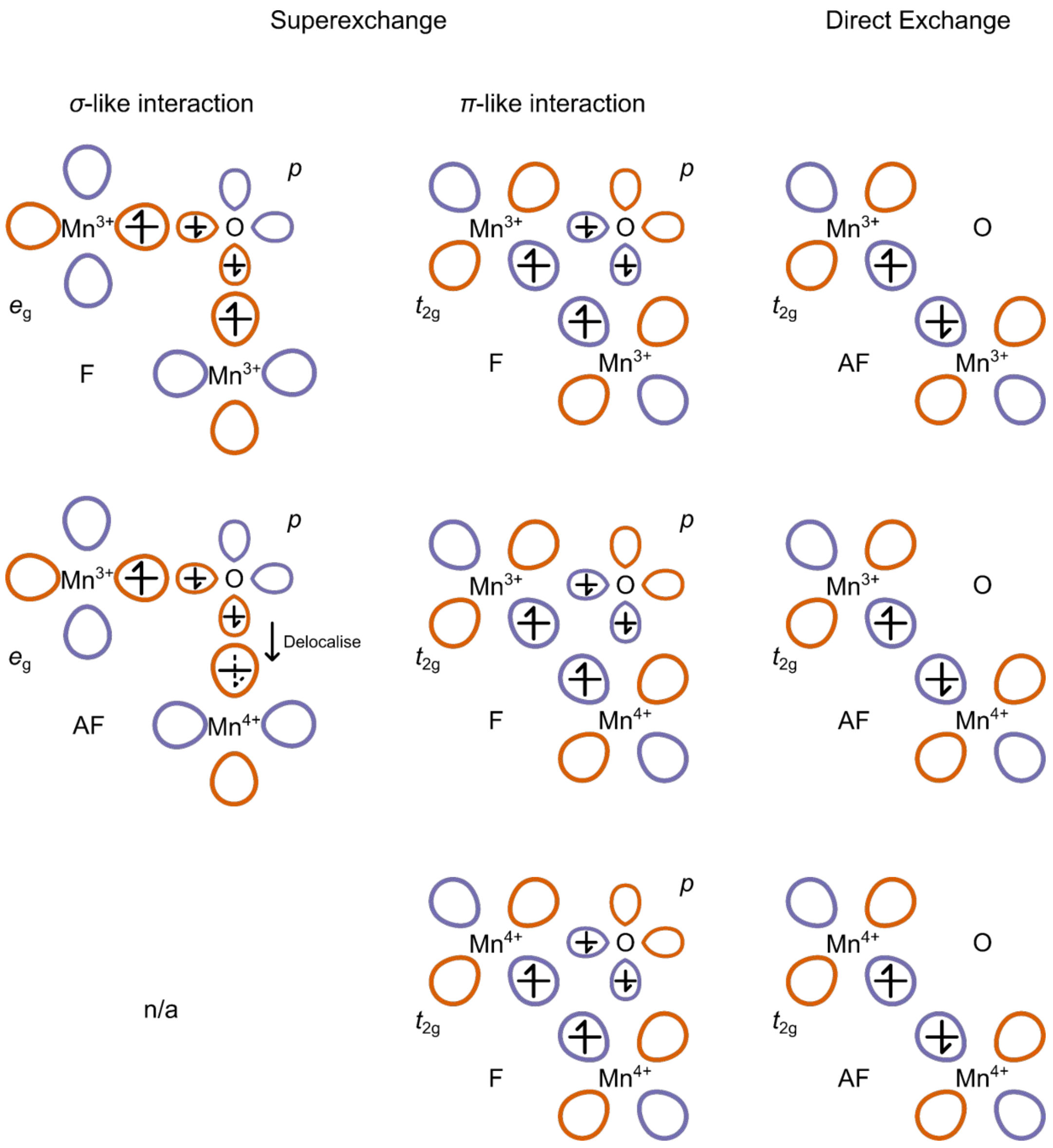

Figure S20: Schematics of the exchange interactions in NMMO. Note that all interactions in NMMO are 90°-like and occur between Mn^{3+} and Mn^{3+}, Mn^{3+} and Mn^{4+} or Mn^{4+} and Mn^{4+}. The interactions are labelled as Ferromagnetic, F, or Antiferromagnetic, AF. Dashed lines indicate spin density which is transferred to a centre, whilst solid lines indicate spins which are already present and have been polarised.

7. Additional *Operando* EPR Electrochemical Data

Here, an electrolyte of $NaPF_6$ in EC:EMC:DEC (1:1:1 by volume) was chosen, as it was found that the standard PC electrolyte strongly attenuates the signal, owing to its high dielectric constant. To assess the effect of changing electrolyte (for *operando* EPR experiments) on the electrochemical performance of NMMO, a Swagelok half-cell of NMMO against Na metal was constructed using the same electrolyte as in the *operando* EPR cell (i.e., 1 M $NaPF_6$ in a 1:1:1 by volume mixture of ethylene carbonate (EC), dimethyl carbonate (DMC) and diethyl carbonate (DEC)). The voltage profile for the first charge/discharge cycle is plotted in Figure S21. These *operando* results are therefore anticipated to be consistent with the *ex situ* data collected.

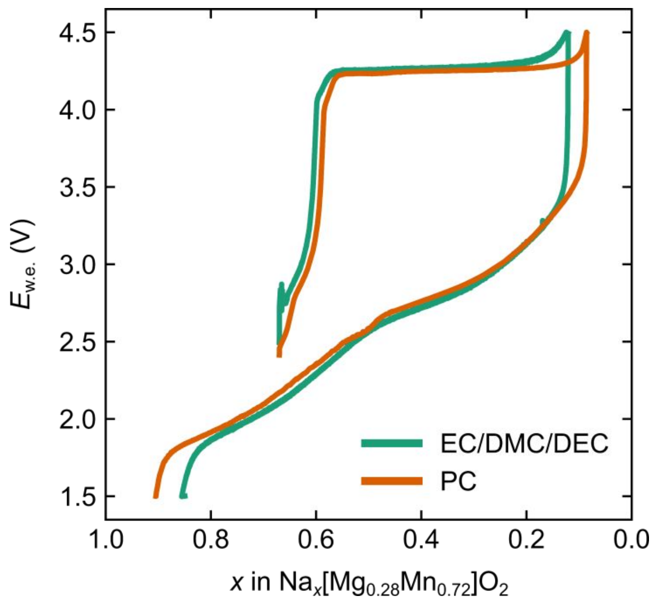

Figure S21: Voltage profiles for an NMMO vs Na metal half cell in two different electrolytes: 1 M $NaPF_6$ in a 1:1:1 (by volume) mixture of ethylene carbonate (EC), dimethyl carbonate (DMC) and diethyl carbonate (DEC) and 1 M $NaPF_6$ in propylene carbonate (PC).

8. *Operando* EPR Background

The *operando* EPR cell exhibits a severe background, due to the metallic components in the cell (Al and Cu foils and wires), in addition to the severe attenuation of the signal by the electrolyte present: this background is shown in Figure

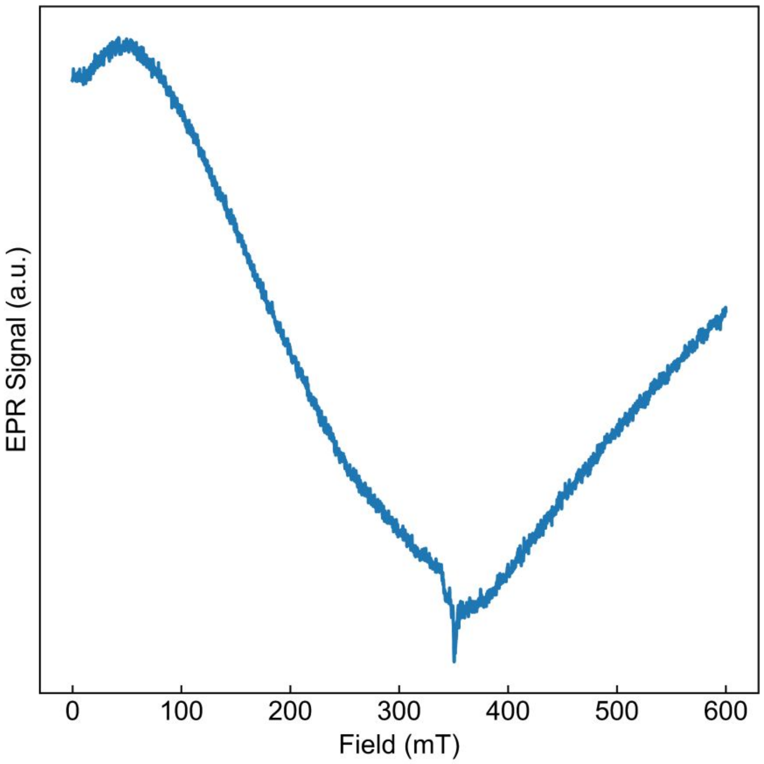

Figure S22: Background EPR spectrum of the *operando* EPR cell, recorded under the same conditions as the *operando* run. Note that the cathode and ruby were removed, but all other components were present.

9. Additional High-Frequency EPR Results

A Note on *g*-factor differences at different frequencies

The fitted *g*-values obtained from high-frequency EPR are much higher than those seen at X-band. This stems from the large inductance built up when sweeping the field over the large field ranges seen here, meaning that the applied field is not the true field at the sample.[52] Correcting this is, again, challenging, due to the large field range covered: whilst initial field corrections can be applied using a reference, this did not work well for these samples because of the large difference in field sweep rates. Moreover, using the faster field sweep rate applied to our samples for the reference leads to loss of the sharp reference signal, whilst using slower sweep rates for the samples lead to prohibitively long acquisition times.

EasySpin Fitted Field Axis Modification.

The spectra acquired at high frequencies were acquired with a non-linear spacing in magnetic field strength. In EasySpin, spectra are fit such that each data point is assumed to be linear in the field—i.e., even if a non-linear data set is supplied, EasySpin handles the data as if it were

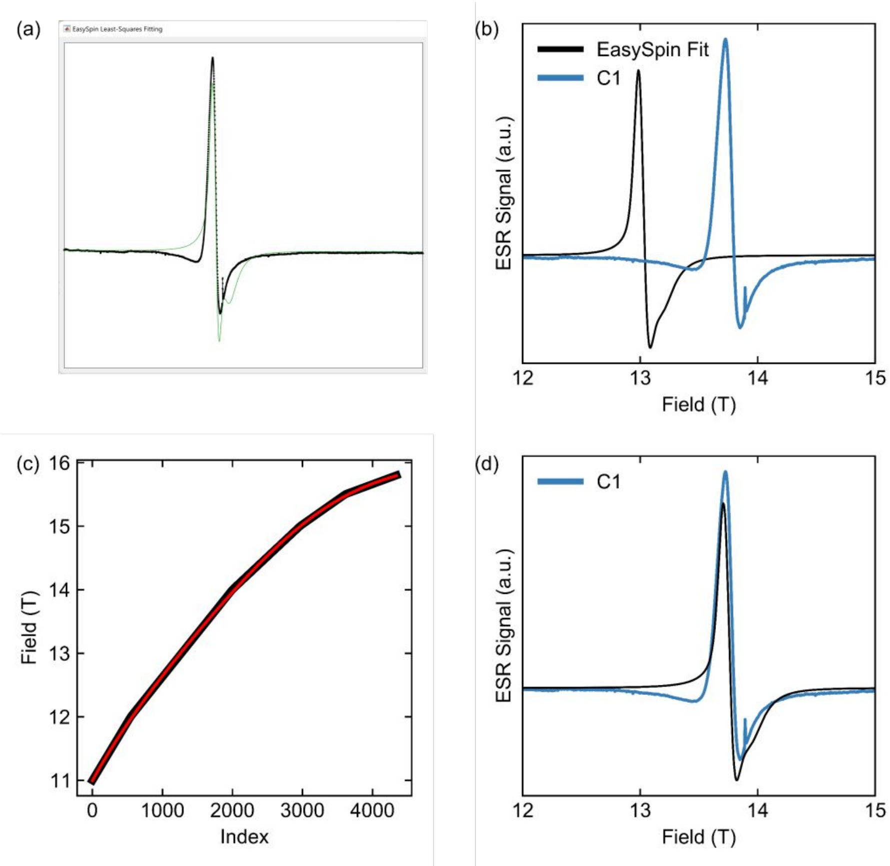

Figure S23: Modification of the EasySpin fitted spectra field axis. In **(a)**, a screenshot of a fitted spectrum from EasySpin; **(b)** shows the same spectrum exported directly and plotted against the observed spectrum. In **(c)**, the data index is plotted against the field value to show its non-linearity (black) and the fit to a fifth-order polynomial function (red); translating the EasySpin field using this curve yields the true fit in **(d)**.

linear by fitting spectra with an *x*-axis of data index number, rather than the absolute field. After the fit is complete and exported, EasySpin computes the field at each point using the input microwave frequency, but assuming the data is linearly spaced. In most cases, this method works, as the data is collected linearly. Here, however, the fitted spectrum is non-linearly offset from the observed spectrum (unlike in EasySpin) [Figure S23(a) and (b)]. To correct for this, a plot of the field from the observed data *vs* it data index was fit to a fifth order polynomial [Figure S23(c)]. From this, the EasySpin-generated spectrum can be translated into the non-linear field to yield the true fit to the spectrum [Figure S23(d)]. The fitted values are therefore not inaccurate and the fit has not been changed: just the way in which it has been plotted changes.

High-Frequency EPR of ^{17}O-Enriched NMMO Cathodes

To further investigate the hyperfine interaction between the unpaired electrons and O centres in NMMO (other than *via* ^{17}O NMR, Figures 8 and 9 in the main text; Figure S11), additional, *ex situ* high-frequency EPR spectra of NMMO were acquired to identify any hyperfine couplings present [Figure S24]. Unfortunately, no coupling and no observable difference in the unenriched and enriched spectra were seen; this is likely because a shift of 3000 ppm at 11.7 T—as seen in the ^{17}O NMR spectra. Our calculations of the Fermi contact interaction give the isotropic hyperfine coupling constant for ^{17}O in the pristine material as approximately −12 to −4 MHz (or -4.0×10^{-4} to -1.3×10^{-4} cm^{-1}). This corresponds to a splitting of between −0.43 and −0.14 mT. At the end of charge, the hyperfine coupling becomes −5 to +45 MHz (-1.6×10^{-4} to $+15 \times 10^{-4}$ cm^{-1}) for the antiferromagnetically coupled Mn^{4+}–O^{-} state, or −150 to +90 MHz (-50×10^{-4} to $+30 \times 10^{-4}$ cm^{-1}) for the trapped O_2 state. This corresponds to a splitting of between −0.18 to +1.6 mT for the antiferromagnetically coupled state and −5.4 to +3.2 mT for the trapped O_2 state. Whilst we can add splittings due to these couplings, these are much narrower than the observed signal and as such we believe it would be too narrow to reasonably fit. No additional broadening was observed, indicating that any unpaired spins on O either relax too quickly to be seen (short T_{2e}, giving a signal which is so broad it is lost to the baseline) or have hyperfine couplings too weak to be seen.

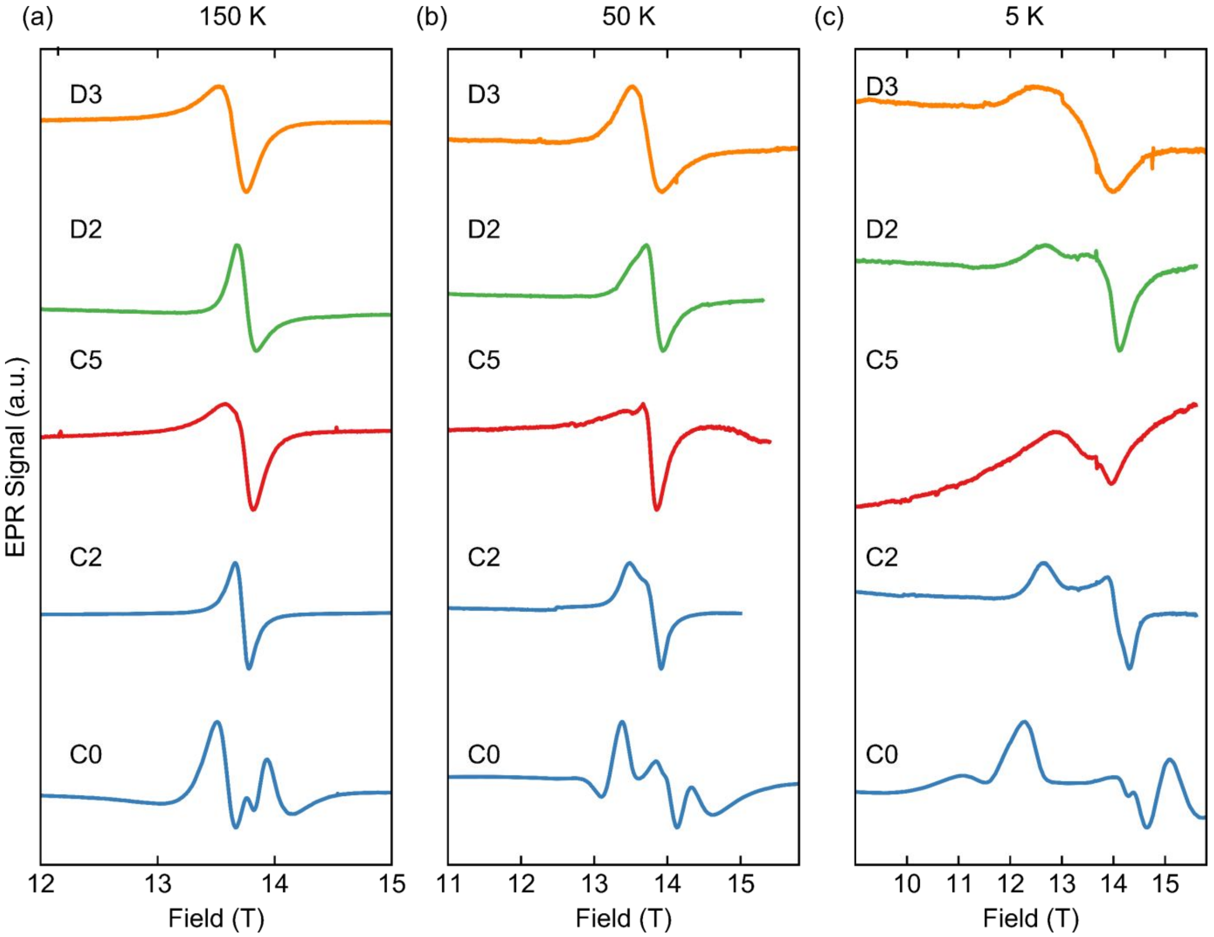

Figure S24: Ex situ continuous wave high-frequency (383.04 GHz) EPR on ^{17}O-enriched NMMO cathodes recorded at **(a)** 150 K, **(b)** 50 K and **(c)** 5 K.

10. X-ray Absorption Spectroscopy Results: Mn *K*-edge XANES and O *K*-edge XAS

Ex Situ Mn *K*-Edge XANES.

To further investigate the redox processes taking place in NMMO over the first charge-discharge cycle, *ex situ* X-ray absorption spectroscopy (XAS) measurements at the Mn and O *K*-edges were carried out [Figure S25 and Figure S26].

During stage 1, the *ex situ* Mn *K*-edge XANES data show only minor changes: the position of the edge moves to higher energies, consistent with oxidation of Mn^{3+} to Mn^{4+}, whilst the pre-edge peaks become slightly more intense [Figure S25(a) and (b)]. The pre-edge features in Mn *K*-edge XANES arise from formally forbidden transitions, which become weakly allowed when 3*d*-4*p* mixing occurs due to a distortion away from an octahedral coordination

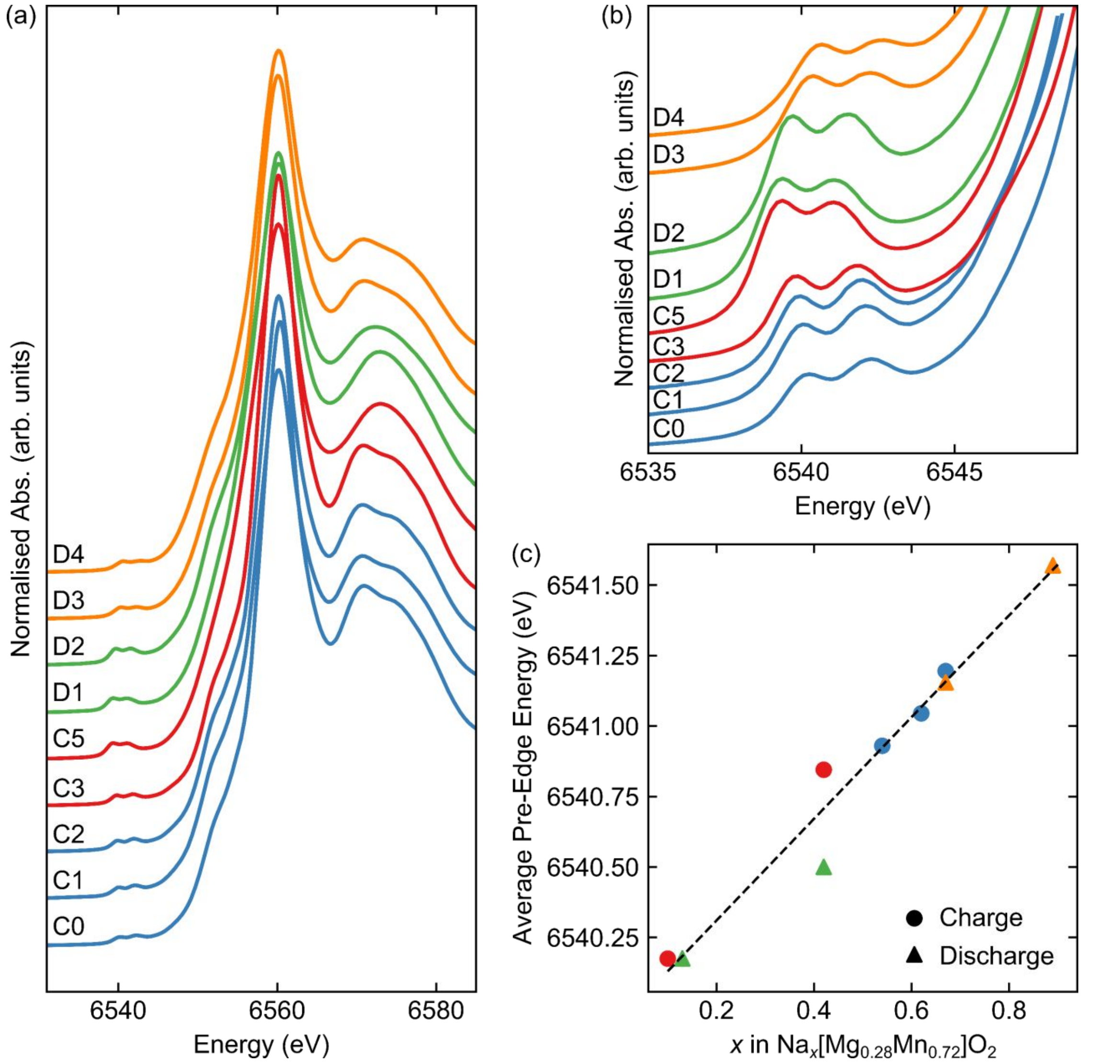

Figure S25: *Ex situ* Mn *K*-edge XANES data for NMMO. **(a)** shows the region around the edge, with the regions which change most—at the pre-edge, edge and post-edge—highlighted in grey. For clarity, the pre-edge region is shown in **(b)**, with the fitted pre-edge energies shown in **(c)**. Fits were achieved using two Pseudo-Voigt peaks and a smooth step function background. A trendline has been added as a guide to the eye.

environment.[53–56] The increase in the intensity of these features therefore suggests stronger 3*d*-4*p* mixing during charge, which likely stems from the decrease in 4*p* energy (due to Mn oxidation). The pre-edge features also move to lower energies on charging, indicating a smaller energy gap between the 1*s* and *d*-*p* hybridised orbitals, again a consequence of oxidation lowering the valence orbital energies whilst leaving core electrons at almost the same energies.

The edge position remains essentially unchanged during stage 2, but three changes take place at the end of charge [Figure S25(a) and (b)]. Firstly, the pre-edge moves to even lower energies and becomes more intense, again suggesting a further lowering of the *d*-*p* valence orbital energies and a greater deviation from octahedral symmetry. Secondly, the shoulder seen near the edge (for example in C3 at around 6545 eV) disappears or is reduced in intensity and merges with the main edge. This shoulder is invariably assigned to the 1*s* to 4*p* transition;[55] the decrease in intensity likely stems from the greater mixing of 3*d* and 4*p* states, leading to a less well-defined 4*p* energy. Finally, the shoulder after the edge (at approximately 6573 eV) becomes a single feature, rather than two overlapping features. This likely reflects the change in local environment of Mn, consistent with our earlier work,[1] where EXAFS (obtained in the post-edge region) indicated that Mg^{2+} migration takes place.

During stages 3 and 4 (on discharge), the edge position again remains essentially unchanged [Figure S25(a)]. The pre-edge feature becomes less intense and moves back to higher energies, consistent with an increase in the energy of the 3*d*-4*p* hybridised orbitals (due to Mn reduction). In addition, the 1*s* to 4*p* shoulder near the edge returns at point D2, suggesting that the electronic states become more distinct, and less *d*-*p* mixing takes place.

Fitting the pre-edge peaks reveals the trend in the pre-edge peak positions: as the Na^+ content decreases, the pre-edge peak energy decreases approximately linearly [Figure S25(c)], consistent with the formation of increasingly hybridised Mn–O orbitals on charge, alongside greater distortion of the local Mn coordination environment.

***Ex Situ* O *K*-Edge XAS.**

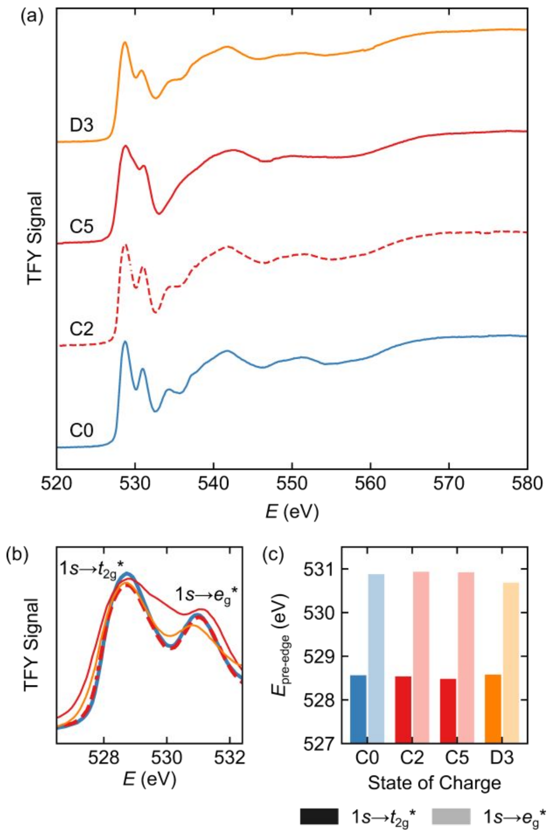

Figure S26: Ex situ O *K*-edge XAS total fluorescence yield (TFY) data for NMMO. **(a)** shows the spectra collected at each state of charge, whilst **(b)** shows the pre-edge region of the spectrum and **(c)** shows the fitted energies of the pre-edge peaks (fit using pseudo-Voigt peaks and a quadratic background).

The O *K*-edge XAS data collected on NMMO at C0, C2, C5 and D3 is shown in Figure S26. In all spectra, a large pre-edge feature is seen between *ca*. 527 eV and 533 eV, which is ascribed to a transition from the 1*s* state to the bound, hybridised Mn(3*d*)–O(2*p*) states. The lower-energy peak corresponds to the 1*s* to Mn(3*d*, t_{2g}*)–O(2*p*) transition, whilst the higher-energy peak corresponds to the 1*s* to Mn(3*d*, e_g*)–O(2*p*) transition. Those are referred to as the t_{2g}* peak and e_g* peak hereafter.[57,58] Oxidising Mn (during charge) is anticipated to decrease the energy of the Mn 3*d* states, improve overlap with the O orbitals and therefore increase the energy of the partially occupied Mn–O anti-bonding hybridised state, giving a lower O pre-edge position. The intensity of the pre-edge peak depends on the extent of mixing between the O 1*s* state and the Mn(3*d*)–O(2*p*) state and therefore depends on the energy difference of the core and hybridised state, as well as the symmetries of these states. The edge, meanwhile, represents the energy required to promote the electron from the O 1*s* state to the vacuum. Higher edge energies correspond to lower energy 1*s* states and higher oxidation states.

In NMMO, the pre-edge peaks are most intense at C0, suggesting, from the perspective of O, the strongest degree of mixing between the Mn 3*d* states and O 2*p* states [Figure S26(b)]. On charging to C2, the pre-edge intensities decrease and the energy of the t_{2g}* decreases slightly and the energy of the e_g* peak increases [Figure S26(c)]. The changes in energy can be ascribed to the oxidation of Mn^{3+} to Mn^{4+}: removing electrons from the e_g* band raises the

energy of these unoccupied states whilst lowering the energy of the occupied t_{2g}* states. The decrease in intensity indicates less mixing of the Mn 3*d* and O 2*p* orbitals.

At C5, the t_{2g}* and e_g* peaks both move to higher energies; the intensity of the t_{2g}* peak lies between that seen at C0 and C2, whilst the intensity of the e_g* peak increases (beyond the intensity seen at C0). In addition, a shoulder peak is seen between the t_{2g}* and e_g* peaks, lying slightly closer in energy to the t_{2g}* peak. The increase in energy suggests that the hybridised Mn(3*d*)–O(2*p*) states become higher in energy, perhaps suggesting oxidation of O (due to a loss of full-shell stability), whilst the increased intensity compared to C2 indicates stronger mixing of the Mn(3*d*) and O(2*p*) orbitals, suggesting more covalent interactions (consistent with the large C_Q obtained at C5 in ^{17}O NMR). The origin of these increases in energy and the appearance of the shoulder peak are further discussed in the next section.

Discharging to D3 results in the disappearance of the shoulder peak and the decrease in energy of both the t_{2g}* and e_g* peaks (compared to the peaks at C5). The intensities of both peaks decreases compared to C5, with the t_{2g}* having a slightly lower intensity than C5 and e_g* being less intense than at any other *ex situ* points. The changes in intensity suggest that the mixing between the Mn(3*d*)–O(2*p*) states decreases compared to C5 (and also C0); the energy changes indicate a decrease in the energy of the Mn(3*d*)–O(2*p*) states compared to C5 and C0.

11. References

(1) Bassey, E. N.; Reeves, P. J.; Jones, M. A.; Lee, J.; Seymour, I. D.; Cibin, G.; Grey, C. P. Structural Origins of Voltage Hysteresis in the Na-Ion Cathode P2–Na0.67[Mg0.28Mn0.72]O2: A Combined Spectroscopic and Density Functional Theory Study. *Chem. Mater.* **2021**, *33* (13), 4890–4906. https://doi.org/10.1021/acs.chemmater.1c00248.

(2) Bassey, E. N.; Seymour, I. D.; Bocarsly, J. D.; Keen, D. A.; Pintacuda, G.; Grey, C. P. Superstructure and Correlated Na+ Hopping in a Layered Mg-Substituted Sodium Manganate Battery Cathode Are Driven by Local Electroneutrality. *Chem. Mater.* **2023**, *35* (24), 10564–10583. https://doi.org/10.1021/acs.chemmater.3c02180.

(3) Wasylishen,R.; Ashbrook, S.; Wimperis, S. Vega, A. J., Quadrupolar Nuclei in Solids. In *NMR of Quadrupolar Nuclei in Solid Materials*; Wiley, 2012; pp 17–44.

(4) Hung, I.; Zhou, L.; Pourpoint, F.; Grey, C. P.; Gan, Z. Isotropic High Field NMR Spectra of Li-Ion Battery Materials with Anisotropy >1 MHz. *J. Am. Chem. Soc.* **2012**, *134* (4), 1898–1901. https://doi.org/10.1021/ja209600m.

(5) Antzutkin, O. N.; Shekar, S. C.; Levitt, M. H. Two-Dimensional Sideband Separation in Magic-Angle-Spinning NMR. *J. Magn. Reson. A* **1995**, *115* (1), 7–19. https://doi.org/10.1006/JMRA.1995.1142.

(6) Antzutkin, O. N. Sideband Manipulation in Magic-Angle-Spinning Nuclear Magnetic Resonance. *Prog. Nucl. Magn. Reson. Spectrosc.* **1999**, *35* (3), 203–266. https://doi.org/10.1016/S0079-6565(99)00010-2.

(7) Tartoni, N.; Thompson, S. P.; Tang, C. C.; Willis, B. L.; Derbyshire, G. E.; Wright, A. G.; Jaye, S. C.; Homer, J. M.; Pizzey, J. D.; Bell, A. M. T.; IUCr. High-Performance X-Ray Detectors for the New Powder Diffraction Beamline I11 at Diamond. *J. Synchrotron Radiat.* **2008**, *15* (1), 43–49. https://doi.org/10.1107/S0909049507046250.

(8) Thompson, S. P.; Parker, J. E.; Potter, J.; Hill, T. P.; Birt, A.; Cobb, T. M.; Yuan, F.; Tang, C. C. Beamline I11 at Diamond: A New Instrument for High Resolution Powder Diffraction. *Rev. Sci. Instrum.* **2009**, *80* (7), 075107. https://doi.org/10.1063/1.3167217.

(9) Coelho, A. A. *TOPAS* and *TOPAS-Academic* : An Optimization Program Integrating Computer Algebra and Crystallographic Objects Written in C++. *J. Appl. Crystallogr.* **2018**, *51* (1), 210–218. https://doi.org/10.1107/S1600576718000183.

(10) Kresse, G.; Hafner, J. Ab Initio Molecular Dynamics for Liquid Metals. *Phys. Rev. B* **1993**, *47* (1), 558–561. https://doi.org/10.1103/PhysRevB.47.558.

(11) Kresse, G.; Hafner, J. Ab Initio Molecular-Dynamics Simulation of the Liquid-Metalamorphous- Semiconductor Transition in Germanium. *Phys. Rev. B* **1994**, *49* (20), 14251–14269. https://doi.org/10.1103/PhysRevB.49.14251.

(12) Dovesi, R.; Erba, A.; Orlando, R.; Zicovich-Wilson, C. M.; Civalleri, B.; Maschio, L.; Rérat, M.; Casassa, S.; Baima, J.; Salustro, S.; Kirtman, B. Quantum-Mechanical Condensed Matter Simulations with CRYSTAL. *Wiley Interdiscip. Rev. Comput. Mol. Sci.* **2018**, *8* (4), e1360. https://doi.org/10.1002/wcms.1360.

(13) Erba, A.; Desmarais, J. K.; Casassa, S.; Civalleri, B.; Donà, L.; Bush, I. J.; Searle, B.; Maschio, L.; Edith-Daga, L.; Cossard, A.; Ribaldone, C.; Ascrizzi, E.; Marana, N. L.; Flament, J.-P.; Kirtman, B. CRYSTAL23: A Program for Computational Solid State Physics and Chemistry. *J. Chem. Theory Comput.* **2022**. https://doi.org/10.1021/acs.jctc.2c00958.

(14) Blöchl, P. E. Projector Augmented-Wave Method. *Phys. Rev. B* **1994**, *50* (24), 17953–17979. https://doi.org/10.1103/PhysRevB.50.17953.

(15) Kresse, G.; Joubert, D. From Ultrasoft Pseudopotentials to the Projector Augmented-Wave Method. *Phys. Rev. B* **1999**, *59* (3), 1758–1775. https://doi.org/10.1103/PhysRevB.59.1758.

(16) Anisimov, V. I.; Zaanen, J.; Andersen, O. K. Band Theory and Mott Insulators: Hubbard U Instead of Stoner I. *Phys. Rev. B* **1991**, *44* (3), 943–954. https://doi.org/10.1103/PhysRevB.44.943.

(17) Anisimov, V. I.; Solovyev, I. V.; Korotin, M. A.; Czyyk, M. T.; Sawatzky, G. A. Density-Functional Theory and NiO Photoemission Spectra. *Phys. Rev. B* **1993**, *48* (23), 16929–16934. https://doi.org/10.1103/PhysRevB.48.16929.
(18) Liechtenstein, A. I.; Anisimov, V. I.; Zaanen, J. Density-Functional Theory and Strong Interactions: Orbital Ordering in Mott-Hubbard Insulators. *Phys. Rev. B* **1995**, *52* (8), R5467. https://doi.org/10.1103/PhysRevB.52.R5467.
(19) Zhou, F.; Cococcioni, M.; Marianetti, C. A.; Morgan, D.; Ceder, G. First-Principles Prediction of Redox Potentials in Transition-Metal Compounds with LDA + U. *Phys. Rev. B* **2004**, *70* (23), 235121-1-235121–235128. https://doi.org/10.1103/PhysRevB.70.235121.
(20) Clément, R. J.; Middlemiss, D. S.; Seymour, I. D.; Ilott, A. J.; Grey, C. P. Insights into the Nature and Evolution upon Electrochemical Cycling of Planar Defects in the β-NaMnO 2 Na-Ion Battery Cathode: An NMR and First-Principles Density Functional Theory Approach. *Chem. Mater.* **2016**, *28* (22), 8228–8239. https://doi.org/10.1021/acs.chemmater.6b03074.
(21) Monkhorst, H. J.; Pack, J. D. Special Points for Brillouin-Zone Integrations. *Phys. Rev. B* **1976**, *13* (12), 5188–5192. https://doi.org/10.1103/PhysRevB.13.5188.
(22) Lee, C.; Yang, W.; Parr, R. G. Development of the Colle-Salvetti Correlation-Energy Formula into a Functional of the Electron Density. *Phys. Rev. B* **1988**, *37* (2), 785–789. https://doi.org/10.1103/PhysRevB.37.785.
(23) Kim, J.; Middlemiss, D. S.; Chernova, N. A.; Zhu, B. Y. X.; Masquelier, C.; Grey, C. P. Linking Local Environments and Hyperfine Shifts: A Combined Experimental and Theoretical 31 P and 7 Li Solid-State NMR Study of Paramagnetic Fe(III) Phosphates. *J. Am. Chem. Soc.* **2010**, *132* (47), 16825–16840. https://doi.org/10.1021/ja102678r.
(24) Clément, R. J.; Pell, A. J.; Middlemiss, D. S.; Strobridge, F. C.; Miller, J. K.; Whittingham, M. S.; Emsley, L.; Grey, C. P.; Pintacuda, G. Spin-Transfer Pathways in Paramagnetic Lithium Transition-Metal Phosphates from Combined Broadband Isotropic Solid-State MAS NMR Spectroscopy and DFT Calculations. *J. Am. Chem. Soc.* **2012**, *134* (41), 17178–17185. https://doi.org/10.1021/ja306876u.
(25) Catti, M.; Valerio, G.; Dovesi, R.; Causà, M. Quantum-Mechanical Calculation of the Solid-State Equilibrium MgO+α-Al2O3 ⇄ MgAl2O4 (Spinel) versus Pressure. *Phys. Rev. B* **1994**, *49* (20), 14179–14187. https://doi.org/10.1103/PhysRevB.49.14179.
(26) Catti, M.; Sandrone, G.; Valerio, G.; Dovesi, R. Electronic, Magnetic and Crystal Structure of Cr2O3 by Theoretical Methods. *J. Phys. Chem. Solids* **1996**, *57* (11), 1735–1741. https://doi.org/10.1016/0022-3697(96)00034-0.
(27) Catti, M.; Sandrone, G.; Dovesi, R. Periodic Unrestricted Hartree-Fock Study of Corundumlike Ti2O3 and V2O3. *Phys. Rev. B* **1997**, *55* (24), 16122–16131. https://doi.org/10.1103/PhysRevB.55.16122.
(28) Schäfer, A.; Horn, H.; Ahlrichs, R. Fully Optimized Contracted Gaussian Basis Sets for Atoms Li to Kr. *J. Chem. Phys.* **1992**, *97* (4), 2571–2577. https://doi.org/10.1063/1.463096.
(29) Kutzelnigg, W.; Fleischer, U.; Schindler, M. The IGLO-Method: Ab-Initio Calculation and Interpretation of NMR Chemical Shifts and Magnetic Susceptibilities; Springer, Berlin, Heidelberg, 1990; pp 165–262. https://doi.org/10.1007/978-3-642-75932-1_3.
(30) Furness, J. W.; Kaplan, A. D.; Ning, J.; Perdew, J. P.; Sun, J. Accurate and Numerically Efficient r2SCAN Meta-Generalized Gradient Approximation. *J. Phys. Chem. Lett.* **2020**, *11* (19), 8208–8215. https://doi.org/10.1021/acs.jpclett.0c02405.
(31) Yang, J. H.; Kitchaev, D. A.; Ceder, G. Rationalizing Accurate Structure Prediction in the Meta-GGA SCAN Functional. *Phys. Rev. B* **2019**, *100* (3), 035132. https://doi.org/10.1103/PhysRevB.100.035132.
(32) Kingsbury, R.; Gupta, A. S.; Bartel, C. J.; Munro, J. M.; Dwaraknath, S.; Horton, M.; Persson, K. A. Performance Comparison of ${r}^{2}\mathrm{SCAN}$ and SCAN metaGGA Density Functionals for Solid Materials via an Automated, High-Throughput Computational Workflow. *Phys. Rev. Mater.* **2022**, *6* (1), 013801. https://doi.org/10.1103/PhysRevMaterials.6.013801.

(33) Puchala, B.; Thomas, J. C.; Natarajan, A. R.; Goiri, J. G.; Behara, S. S.; Kaufman, J. L.; Van der Ven, A. CASM — A Software Package for First-Principles Based Study of Multicomponent Crystalline Solids. *Comput. Mater. Sci.* **2023**, *217*, 111897. https://doi.org/10.1016/j.commatsci.2022.111897.
(34) Bain, G. A.; Berry, J. F. Diamagnetic Corrections and Pascal's Constants. *J Chem Educ* **2008**, *85* (4), 532–536.
(35) Stoll, S.; Schweiger, A. EasySpin, a Comprehensive Software Package for Spectral Simulation and Analysis in EPR. *J. Magn. Reson.* **2006**, *178* (1), 42–55. https://doi.org/10.1016/J.JMR.2005.08.013.
(36) Barra, A. L.; Hassan, A. K.; Janoschka, A.; Schmidt, C. L.; Schünemann, V. Broad-Band Quasi-Optical HF-EPR Spectroscopy: Application to the Study of the Ferrous Iron Center from a Rubredoxin Mutant. *Appl. Magn. Reson.* **2006**, *30* (3), 385–397. https://doi.org/10.1007/BF03166208.
(37) Ravel, B.; Newville, M. ATHENA, ARTEMIS, HEPHAESTUS: Data Analysis for X-Ray Absorption Spectroscopy Using IFEFFIT. *J. Synchrotron Radiat.* **2005**, *12* (4), 537–541. https://doi.org/10.1107/S0909049505012719.
(38) Newville, M. IFEFFIT: Interactive XAFS Analysis and FEFF Fitting. *J. Synchrotron Radiat.* **2001**, *8* (2), 325–327.
(39) Solé, V. A.; Papillon, E.; Cotte, M.; Walter, P.; Susini, J. A Multiplatform Code for the Analysis of Energy-Dispersive X-Ray Fluorescence Spectra. *Spectrochim. Acta Part B At. Spectrosc.* **2007**, *62* (1), 63–68. https://doi.org/10.1016/J.SAB.2006.12.002.
(40) Grimme, S.; Ehrlich, S.; Goerigk, L. Effect of the Damping Function in Dispersion Corrected Density Functional Theory. *J. Comput. Chem.* **2011**, *32* (7), 1456–1465. https://doi.org/10.1002/JCC.21759.
(41) Grimme, S.; Antony, J.; Ehrlich, S.; Krieg, H. A Consistent and Accurate Ab Initio Parametrization of Density Functional Dispersion Correction (DFT-D) for the 94 Elements H-Pu. *J. Chem. Phys.* **2010**, *132* (15), 154104. https://doi.org/10.1063/1.3382344.
(42) Kresse, G.; Furthmüller, J. Efficiency of Ab-Initio Total Energy Calculations for Metals and Semiconductors Using a Plane-Wave Basis Set. *Comput. Mater. Sci.* **1996**, *6* (1), 15–50. https://doi.org/10.1016/0927-0256(96)00008-0.
(43) Himmetoglu, B.; Floris, A.; de Gironcoli, S.; Cococcioni, M. Hubbard-Corrected DFT Energy Functionals: The LDA+U Description of Correlated Systems. *Int. J. Quantum Chem.* **2014**, *114* (1), 14–49. https://doi.org/10.1002/qua.24521.
(44) Wetmore, S. D.; Eriksson, L. A.; Boyd, R. J. The Calculation of Accurate 17O Hyperfine Coupling Constants in the Hydroxyl Radical: A Difficult Problem for Current Quantum Chemical Methods. *J. Chem. Phys.* **1998**, *109* (21), 9451. https://doi.org/10.1063/1.477607.
(45) Miller, S. L.; Townes, C. H.; Kotani, M. The Electronic Structure of O2*. *Phys. Rev.* **1953**, *90* (4), 542. https://doi.org/10.1103/PhysRev.90.542.
(46) Massaro, A.; Muñoz-Garciá, A. B.; Prosini, P. P.; Gerbaldi, C.; Pavone, M. Unveiling Oxygen Redox Activity in P2-Type NaxNi0.25Mn0.68O2High-Energy Cathode for Na-Ion Batteries. *ACS Energy Lett.* **2021**, *6* (7), 2470–2480. https://doi.org/10.1021/ACSENERGYLETT.1C01020/SUPPL_FILE/NZ1C01020_SI_001.PDF.
(47) Mortemard de Boisse, B.; Nishimura, S.; Watanabe, E.; Lander, L.; Tsuchimoto, A.; Kikkawa, J.; Kobayashi, E.; Asakura, D.; Okubo, M.; Yamada, A. Highly Reversible Oxygen-Redox Chemistry at 4.1 V in Na4/7−x[□1/7Mn6/7]O2 (□: Mn Vacancy). *Adv. Energy Mater.* **2018**, *8* (20), 1800409.
(48) Fulmer, G. R.; Miller, A. J. M.; Sherden, N. H.; Gottlieb, H. E.; Nudelman, A.; Stoltz, B. M.; Bercaw, J. E.; Goldberg, K. I. NMR Chemical Shifts of Trace Impurities: Common Laboratory Solvents, Organics, and Gases in Deuterated Solvents Relevant to the Organometallic Chemist. *Organometallics* **2010**, *29* (9), 2176–2179. https://doi.org/10.1021/om100106e.

(49) Xing, L.; Wang, C.; Li, W.; Xu, M.; Meng, X.; Zhao, S. Theoretical Insight into Oxidative Decomposition of Propylene Carbonate in the Lithium Ion Battery. *J. Phys. Chem. B* **2009**, *113* (15), 5181–5187. https://doi.org/10.1021/jp810279h.
(50) Arakawa, M.; Yamaki, J. Anodic Oxidation of Propylene Carbonate and Ethylene Carbonate on Graphite Electrodes. *J. Power Sources* **1995**, *54* (2), 250–254. https://doi.org/10.1016/0378-7753(94)02078-H.
(51) Rinkel, B. L. D.; Vivek, J. P.; Garcia-Araez, N.; Grey, C. P. Two Electrolyte Decomposition Pathways at Nickel-Rich Cathode Surfaces in Lithium-Ion Batteries. *Energy Environ. Sci.* **2022**, *15* (8), 3416–3438. https://doi.org/10.1039/D1EE04053G.
(52) Barra, A.-L.; Brunel, L.-C.; Baumann, F.; Schwach, M.; Moscherosch, M.; Kaim, W. High-Frequency (245 GHz) and X-Band EPR Study of Stable Dicopper Radical Complexes. *J Chem Soc* **1999**, 3855–3857.
(53) Farges, F.; Brown, G. E. Ti-Edge XANES Studies of Ti Coordination and Disorder in Oxide Compounds: Comparison between Theory and Experiment. *Phys. Rev. B - Condens. Matter Mater. Phys.* **1997**, *56* (4), 1809–1819. https://doi.org/10.1103/PhysRevB.56.1809.
(54) Farges, F. Ab Initio and Experimental Pre-Edge Investigations of the Mn K -Edge XANES in Oxide-Type Materials. *Phys. Rev. B* **2005**, *71* (15), 155109. https://doi.org/10.1103/PhysRevB.71.155109.
(55) Smart, L. E.; Moore, E. A. *Solid State Chemistry: An Introduction*; CRC Press, 2012.
(56) Chalmin, E.; Farges, F.; Brown, G. E. A Pre-Edge Analysis of Mn K-Edge XANES Spectra to Help Determine the Speciation of Manganese in Minerals and Glasses. *Contrib. Mineral. Petrol.* **2009**, *157* (1), 111–126. https://doi.org/10.1007/s00410-008-0323-z.
(57) Roychoudhury, S.; Qiao, R.; Zhuo, Z.; Li, Q.; Lyu, Y.; Kim, J. H.; Liu, J.; Lee, E.; Polzin, B. J.; Guo, J.; Yan, S.; Hu, Y.; Li, H.; Prendergast, D.; Yang, W. Deciphering the Oxygen Absorption Pre-Edge: A Caveat on Its Application for Probing Oxygen Redox Reactions in Batteries. *Energy Environ. Mater.* **2021**, *4* (2), 246–254. https://doi.org/10.1002/EEM2.12119.
(58) Frati, F.; Hunault, M. O. J. Y.; De Groot, F. M. F. Oxygen K-Edge X-Ray Absorption Spectra. *Chem. Rev.* **2020**, *120* (9), 4056–4110. https://doi.org/10.1021/acs.chemrev.9b00439.
